# Rapid *de novo* assembly of animal-microbe biofilter to mitigate seabed methane leakage

**DOI:** 10.1093/nsr/nwag266

**Published:** 2026-05-14

**Authors:** Qianyong Liang, Longhui Deng, Ruize Xie, Xinyue Liu, Xi Xiao, Jialin Hou, Jing Wang, Weikang Sui, Ningyuan Lu, Zian Tong, Danyue Huang, Yanwei Wang, Yingchun Han, Jing Zhao, Binbin Guo, Wei Zhang, Minghui Geng, Tianxin Ren, Wenqi Ye, Zheng Xiong, Liang Dong, S Emil Ruff, Christof Meile, Jun Tao, Xiyang Dong, Fengping Wang

**Affiliations:** Guangzhou Marine Geological Survey, China Geological Survey, Guangzhou 511457, China; National Engineering Research Center of Gas Hydrate Exploration and Development, Guangzhou 511457, China; State Key Laboratory of Submarine Geoscience, Key Laboratory of Polar Ecosystem and Climate Change, Ministry of Education, Shanghai Key Laboratory of Polar Life and Environment Sciences, and School of Oceanography, Shanghai Jiao Tong University, Shanghai 200030, China; State Key Laboratory of Submarine Geoscience, Key Laboratory of Polar Ecosystem and Climate Change, Ministry of Education, Shanghai Key Laboratory of Polar Life and Environment Sciences, and School of Oceanography, Shanghai Jiao Tong University, Shanghai 200030, China; Key Laboratory of Marine Biogenetic Resources, Third Institute of Oceanography, Ministry of Natural Resources, Xiamen 361005, China; Guangzhou Marine Geological Survey, China Geological Survey, Guangzhou 511457, China; National Engineering Research Center of Gas Hydrate Exploration and Development, Guangzhou 511457, China; State Key Laboratory of Submarine Geoscience, Key Laboratory of Polar Ecosystem and Climate Change, Ministry of Education, Shanghai Key Laboratory of Polar Life and Environment Sciences, and School of Oceanography, Shanghai Jiao Tong University, Shanghai 200030, China; State Key Laboratory of Submarine Geoscience, Key Laboratory of Polar Ecosystem and Climate Change, Ministry of Education, Shanghai Key Laboratory of Polar Life and Environment Sciences, and School of Oceanography, Shanghai Jiao Tong University, Shanghai 200030, China; State Key Laboratory of Submarine Geoscience, Key Laboratory of Polar Ecosystem and Climate Change, Ministry of Education, Shanghai Key Laboratory of Polar Life and Environment Sciences, and School of Oceanography, Shanghai Jiao Tong University, Shanghai 200030, China; State Key Laboratory of Submarine Geoscience, Key Laboratory of Polar Ecosystem and Climate Change, Ministry of Education, Shanghai Key Laboratory of Polar Life and Environment Sciences, and School of Oceanography, Shanghai Jiao Tong University, Shanghai 200030, China; State Key Laboratory of Submarine Geoscience, Key Laboratory of Polar Ecosystem and Climate Change, Ministry of Education, Shanghai Key Laboratory of Polar Life and Environment Sciences, and School of Oceanography, Shanghai Jiao Tong University, Shanghai 200030, China; State Key Laboratory of Submarine Geoscience, Key Laboratory of Polar Ecosystem and Climate Change, Ministry of Education, Shanghai Key Laboratory of Polar Life and Environment Sciences, and School of Oceanography, Shanghai Jiao Tong University, Shanghai 200030, China; State Key Laboratory of Submarine Geoscience, Key Laboratory of Polar Ecosystem and Climate Change, Ministry of Education, Shanghai Key Laboratory of Polar Life and Environment Sciences, and School of Oceanography, Shanghai Jiao Tong University, Shanghai 200030, China; Key Laboratory of Marine Biogenetic Resources, Third Institute of Oceanography, Ministry of Natural Resources, Xiamen 361005, China; Guangzhou Marine Geological Survey, China Geological Survey, Guangzhou 511457, China; Guangzhou Marine Geological Survey, China Geological Survey, Guangzhou 511457, China; National Engineering Research Center of Gas Hydrate Exploration and Development, Guangzhou 511457, China; Guangzhou Marine Geological Survey, China Geological Survey, Guangzhou 511457, China; National Engineering Research Center of Gas Hydrate Exploration and Development, Guangzhou 511457, China; Guangzhou Marine Geological Survey, China Geological Survey, Guangzhou 511457, China; National Engineering Research Center of Gas Hydrate Exploration and Development, Guangzhou 511457, China; State Key Laboratory of Submarine Geoscience, Key Laboratory of Polar Ecosystem and Climate Change, Ministry of Education, Shanghai Key Laboratory of Polar Life and Environment Sciences, and School of Oceanography, Shanghai Jiao Tong University, Shanghai 200030, China; State Key Laboratory of Submarine Geoscience, Key Laboratory of Polar Ecosystem and Climate Change, Ministry of Education, Shanghai Key Laboratory of Polar Life and Environment Sciences, and School of Oceanography, Shanghai Jiao Tong University, Shanghai 200030, China; State Key Laboratory of Submarine Geoscience, Key Laboratory of Polar Ecosystem and Climate Change, Ministry of Education, Shanghai Key Laboratory of Polar Life and Environment Sciences, and School of Oceanography, Shanghai Jiao Tong University, Shanghai 200030, China; State Key Laboratory of Submarine Geoscience, Key Laboratory of Polar Ecosystem and Climate Change, Ministry of Education, Shanghai Key Laboratory of Polar Life and Environment Sciences, and School of Oceanography, Shanghai Jiao Tong University, Shanghai 200030, China; Marine Biological Laboratory, Woods Hole, MA 02543, USA; University of Bremen, Bremen 28359, Germany; Department of Marine Sciences, University of Georgia, Athens, GA 30602, USA; Guangzhou Marine Geological Survey, China Geological Survey, Guangzhou 511457, China; Key Laboratory of Marine Biogenetic Resources, Third Institute of Oceanography, Ministry of Natural Resources, Xiamen 361005, China; State Key Laboratory of Submarine Geoscience, Key Laboratory of Polar Ecosystem and Climate Change, Ministry of Education, Shanghai Key Laboratory of Polar Life and Environment Sciences, and School of Oceanography, Shanghai Jiao Tong University, Shanghai 200030, China

**Keywords:** gas hydrate destabilization, seabed methane leakage, rapid deep-sea responses, methane biofilter assembly, natural mitigation capacity

## Abstract

Anthropogenic and climatic perturbations threaten to destabilize gas hydrates and release methane from its vast subseafloor reservoir. Yet the deep-sea ecosystem responses remain poorly understood. Our multi-year, *in situ* monitoring of a human-induced methane seep unveiled an exceptionally fast, *de novo* establishment of a methane-consuming ecosystem within 1–2 years, followed by a rapid succession toward natural mature seeps. Integrated biogeochemical and molecular analyses revealed an unexpectedly parallel proliferation of aerobic methanotrophs (*Methyloprofundus*), anaerobic methanotrophs (ANME-2e, ANME-3), and opportunistic fauna bioturbating the seabed to >50 cm depths. Within this intensively mixed zone, active animal-microbe interactions sustained rapid methane removal (30–60 mmol/m^2^/day) through intricate carbon, nitrogen, and sulfur redox coupling. Our work demonstrates that abrupt methane leakage can form an effective animal-microbe ‘methane biofilter’ far quicker than previously estimated, producing new insights into benthic natural mitigation capacity and limit that are critical for risk assessments and climate projections under increasing seabed methane efflux.

## Teaser

Multi-year, in-situ monitoring of a deep-sea methane leak from its very start shows that an effective ``methane biofilter'' driven by an intricate coordination between benthic methane-oxidizing microbes and opportunistic animals can develop within just 1-2 years, consuming the greenhouse gas much faster than previously thought.

## INTRODUCTION

Marine sediments hold Earth’s largest reservoir of methane, a clean fossil energy resource and potent greenhouse gas [1]. Methane is stored in sediment in its dissolved and gaseous forms, or as solid gas hydrate, a crystalline compound of water and hydrocarbons that harbors 500–2000 gigatons of carbon (Gt C) worldwide [1]. Mounting evidence indicates that methane efflux from the seabed is greater than previously recognized [2–5] and likely to be exacerbated by global change [6–8]. Climate-driven increases in bottom-water temperature [7,9,10], coupled with expanding oceanic resource exploration and mining activities [11,12], threaten to destabilize hydrate reservoirs and enhance the advective efflux of methane. This may lead to increasing, yet unconstrained amounts of methane escape to the upper hydrosphere or even atmosphere [13–15]. Hydrate dissociation-driven emission of methane was estimated to add 0.4–0.5°C to the warming initially caused by anthropogenic activities over millennial timescales [16]. Aerobic oxidation of methane released from seabed consumes oxygen in the seawater, and together with the warming-induced reductions in oxygen solubility and ocean ventilation, might lead to expanding ocean hypoxia [17,18].

Marine benthic ecosystems form a critical biological barrier—the ‘methane biofilter’ that effectively mitigates methane emission from the seabed [19,20]. The efficiency of this filter hinges on complex microbial and faunal communities sustained by methane seepage [21,22]. Along global continental slopes, deeply sourced methane-rich fluids discharge at seafloors, forming ‘methane seeps’ that sustain some of the most prolific chemosynthetic ecosystems on Earth [19]. Although only covering less than 0.05% of the slope area, seeps account for 10%–20% of the total methane oxidation along continental slopes, representing a globally relevant gatekeeper that restricts seabed methane emission [22,23].

A fundamental knowledge gap, however, is the timescale and mechanism of *de novo* biofilter development following a sudden methane efflux. Classical models, constrained by the slow growth of anaerobic methanotrophic archaea (ANME-1, ANME-2; doubling times of months to years [24–26]) and the decadal to centennial succession of macrofaunal communities [27–30], suggest a potential ‘window’ of 60–100 years for substantial methane escape to the upper ocean before an efficient biofilter forms [14,23,26]. The discovery of active methane-oxidizing communities in freshly erupted muds surrounding the Haakon Mosby mud volcano suggested the potential of rapid methanotrophic response [31,32]. Current constraints, however, rely on indirect evidence from space-for-time substitution [31,32] or monitoring from an arbitrary, post‑initiation timepoint of established natural seeps [32,33]. Consequently, the pace and mechanism of *de novo* biofilter development, whether following natural or anthropogenic methane leakage, remain uncertain [29]. Direct *in situ* tracking of full ecological succession from a defined ‘time zero’ of methane release is essential to close this knowledge gap. Such research is currently lacking, representing an urgent research priority as industrial activity in the ocean and the risk of human- or climate-induced methane leakage continue to increase [34–37].

**Figure 1.**
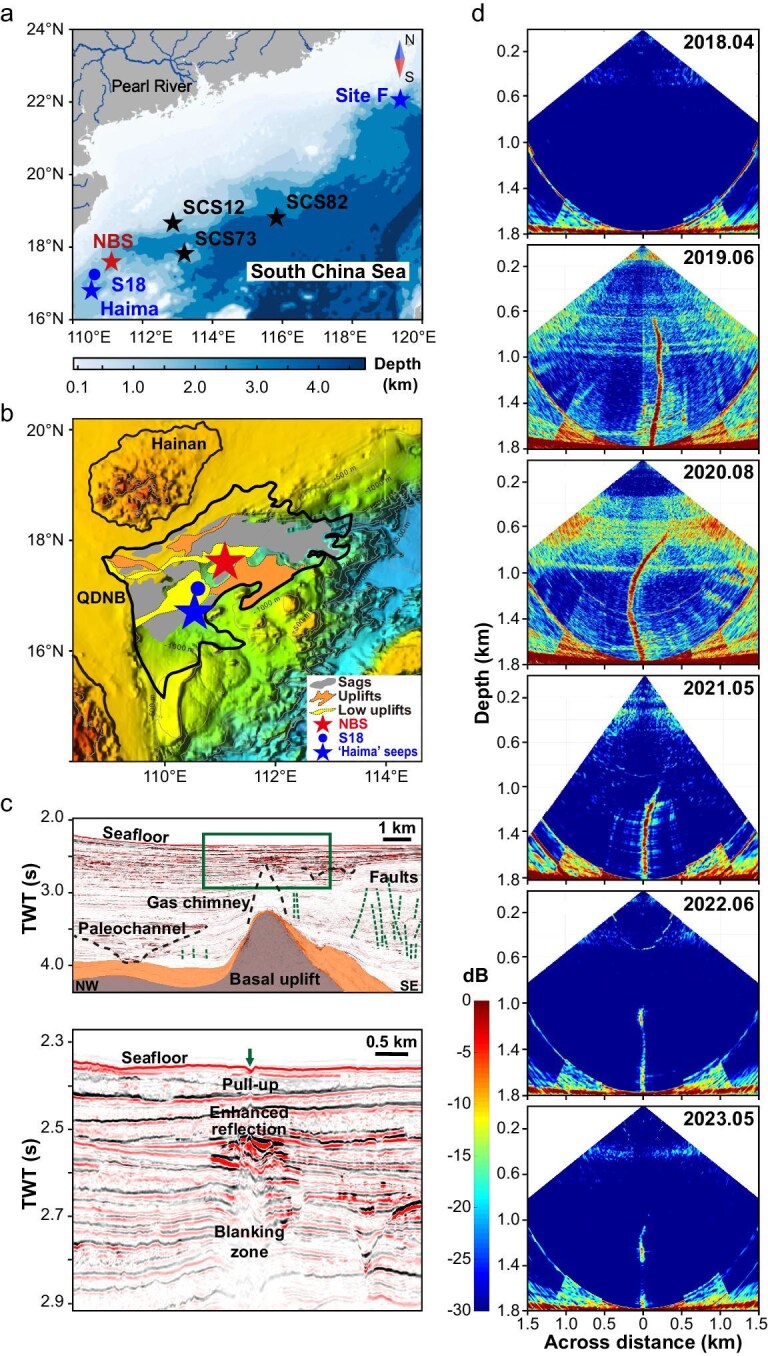
Tracking methane leakage triggered by gas hydrate drilling. (a) Map showing bathymetric features and sampling sites in the South China Sea (SCS), including the ‘Newborn Seep (NBS),’ mature cold seeps (S18, ‘Haima’ and ‘Site F’ cold seeps), and non-seep continental slope sites (SCS12, SCS73, SCS82). Note that two non-seep background sites 19-NS-BG1 and 19-NS-BG2 were sampled ∼200 m away from the discharge center of NBS in 2019, before the spreading of leakage impact on seafloor (Fig. S1). (b) Geological setting of the study region within Qiongdongnan Basin (QDNB) of the SCS. (c) Seismic reflection profiles showing shallow subsurface structures, fluid pathways, and enhanced reflections. TWT: two-way traveltime. (d) Water-column acoustic images from April 2018 to May 2023, revealing recurring acoustic flares (high-backscatter zones) indicative of ongoing gas bubble release from the hydrate-bearing seabed.

Here, we report a six-year *in situ* study (2018–2023) that directly tracks the response of a seabed ecosystem to an abrupt methane leakage event triggered by gas hydrate exploration in the South China Sea (SCS, Fig. 1a). We tracked the spatiotemporal changes of seabed communities and sediment geochemistry in the leakage-impacted areas annually (Fig. S1), using advanced underwater cameras, acoustic instruments, chemical sensors, and sampling facilities, combined with biological analyses, geochemical modeling, and isotopic rate measurements in the laboratories. We further compared the newly formed chemosynthetic ecosystem, hereafter called ‘Newborn Seep’(NBS), with both adjacent and distant non-seep and mature seep sites in the SCS (Fig. S2, Table S1). Through this high-resolution capture of ecosystem succession following a precisely timed, human-induced methane leakage, we reveal the *de novo* assembly of a methane biofilter within 1–2 years, catalyzed by the unexpectedly parallel proliferation of anaerobic methanotrophs (ANME-2e, ANME-3), aerobic methanotrophs, and opportunistic macrofauna.

## RESULTS AND DISCUSSION

### Abrupt methane leakage triggered by gas hydrate drilling

The study site is located in the Qiongdongnan Basin of the SCS at a water depth of 1766 m, where gas hydrate inventory was estimated to reach ∼6.5 × 10^9^ tons of carbon over an area of ∼6 × 10^4^ km^2^ (Fig. 1a and b) [38]. High-resolution 3D seismic data reveal that this site is connected to a subseafloor gas chimney that originates from a basal uplift, appearing as a seismic blanking zone with base width of ∼1 km and narrowing upwards to less than 0.2 km (Fig. 1c). Above the gas chimney, the pull-up enhanced reflections indicate the presence of highly saturated gas hydrates within the leakage pathway. In 2018, a drilling expedition was conducted to assess the gas hydrate resource [39]. Before drilling, the seafloor at the drill site appeared as a ‘bare’ sedimentary floor with scarce benthic fauna and no gas plume (Fig. S2). A borehole in diameter of ∼22 cm was drilled using the logging tools from Schlumberger and subsequently sealed with heavy active mud [39]. While no gas discharge was observed after sealing the borehole in 2018, a distinct gas plume was detected by multibeam echosounders since 2019, suggesting the initiation of methane leakage during 2018–2019 (Fig. 1d). The plume reached ∼1.2 km and ∼0.8 km above the seafloor during 2019–2020 and 2021–2023, respectively. Elevated methane concentrations (up to 10 μmol L^−1^) were detected by underwater sensors in the water column above the discharge center, indicating that those gas bubbles were rich in methane (Fig. S3).

### Rapid and drastic seabed ecosystem transformation

**Figure 2.**
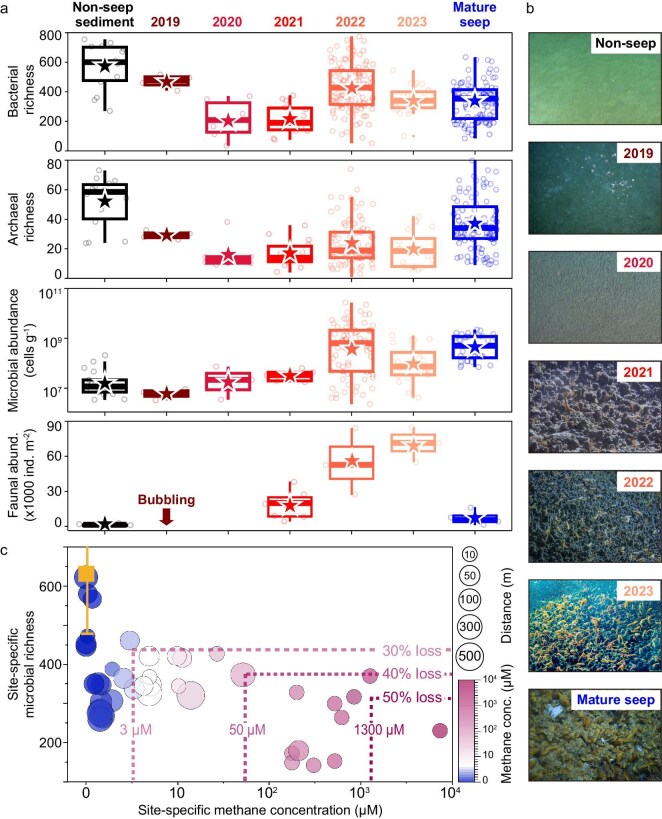
Ecological changes of seabed ecosystem following methane leakage. (a) Variations of bacterial and archaeal richness, microbial and macrofaunal abundances. Bacterial and archaeal richness were calculated as the quantities of amplicon sequence variants (ASVs) based on 16S rRNA gene sequencing. Microbial abundance was quantified based on qPCR of the bacterial and archaeal 16S rRNA genes. Macrofaunal abundance was determined by combining the sieving-based method and image analysis. The ‘non-seep sediment’ group includes only data from the adjacent non-seep background sites, while the ‘mature seep’ group includes data from the ‘Haima’ and ‘Site F’ cold seeps in the South China Sea. Boxplots show means (asterisks), medians, quartiles, and data ranges (same below). (b) Representative seafloor images of the pre- and post-drilling seafloors, as well as the typical mature seep ecosystem (mussel bed) at ‘Haima’ cold seeps. (c) Negative correlation between the site-specific microbial richness and methane concentration. The site-specific values were calculated as the mean values across different sediment depths at each site. The size of meter symbol refers distance from the drilling site at the center of ‘Newborn Seep’, while symbol color indicates methane concentration. The yellow symbol and error bar indicate the microbial richness in the adjacent non-seep background sites.

The seabed ecosystem at the leakage site underwent a rapid and drastic transformation following methane leakage. Within 2 years of the leakage onset, bacterial and archaeal richness in surface sediments (0–100 cm) within 20 m of the discharge center declined by >50% (Fig. 2a). Concurrent declines in other microbial diversity indices (Chao1, Shannon, and Simpson) were also observed (Fig. S4). Following the initial decline, microbial richness appeared to rebound since 2020, indicating a rapid seabed ecosystem restructuring. In contrast, microbial abundance remained stable or even increased following the methane leakage, suggesting an immediate, active response of seabed microbial community to abrupt methane input. Concurrently, the seafloor morphology was radically altered. Within 1 year (2020–2021), the barren sediment transformed into an extensive ‘worm bed’ (Fig. 2b), with the proliferation of Spionid polychaetes, Acrocirrid polychaetes, and Metridinid copepods (Fig. S5A), demonstrating the remarkable capability of deep-sea opportunistic invertebrates to rapidly colonize a newly formed methane seep. Bayesian δ¹³C mixing model calculations further show the substantial contributions of methane-derived carbon (20%–70%) to the faunal diets (Fig. S5B). The observed suspension- and/or deposit-feeding behaviors of these dominant fauna suggest that prey-predator interactions, instead of symbiosis that is prevalent in mature cold seeps, drove the highly efficient methane-based trophic transfer. Dense populations of opportunistic polychaetes observed in natural seeps worldwide had long been proposed as an indicator of early-stage seep ecosystems [28,40–43], a notion now directly corroborated by our post-leakage *in situ* monitoring. Our findings further demonstrate a rapid, coordinated response by prokaryotic and eukaryotic communities, which functionally integrated within 2–3 years to establish an intricate biofilter following methane release. These quantitative and mechanistic insights are crucial to refine both the timeline and trajectory of previous seep biota succession models [27,28].

The impact of methane leakage expanded rapidly, affecting the wider seabed ecosystem within 2–3 years. Extensive sampling at >40 sites surrounding the discharge center revealed the occurrence of elevated methane concentrations (>10 fold of non-seep background values) in surface sediments even at 500-m distance from the discharge center (Fig. 2c). This was accompanied by a >30% reduction in microbial richness, aligned with the previously reported pattern of reduced diversity in natural seeps [44,45], demonstrating a rapid lateral propagation of the leakage impact (Fig. 2c). Synthesis of data from all sites reveals the negative correlations of microbial richness with methane and hydrogen sulfide concentration (Fig. 2c; linear regression model, both F_1,40_ > 10, *P* < 0.01). These correlations suggest that even micro-molar increases of sediment methane and hydrogen sulfide concentrations, likely indicative of anoxic conditions, are linked to significant microbial diversity decreases.

### Swift *de novo* assembly of a methane-consuming microbiome

**Figure 3.**
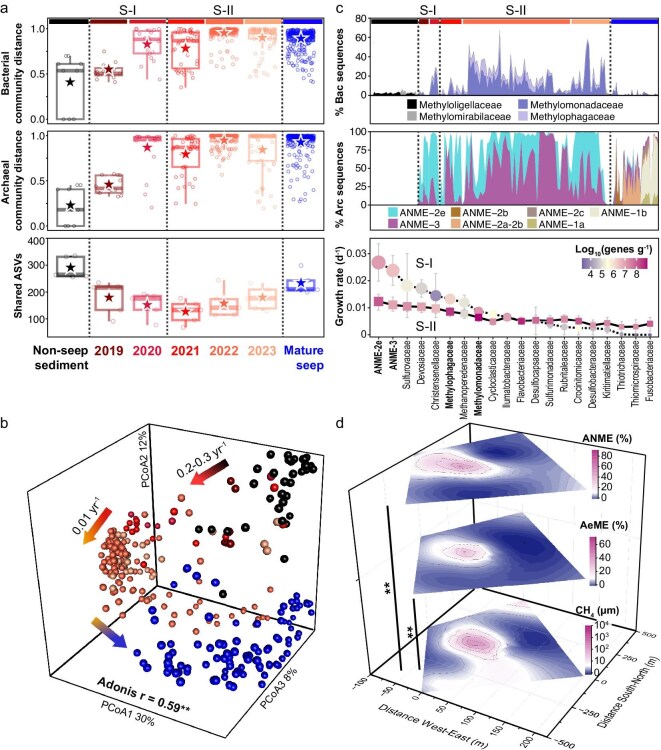
Spatiotemporal variations of sediment microbial communities in response to methane leakage. (a) Boxplots showing shifts in bacterial and archaeal Bray-Curtis community distance (dissimilarity) and shared amplicon sequence variants (ASVs) in the Newborn Seep and mature seep sediments, compared with the non-seep background sediments. Stage-I (S-I) and Stage-II (S-II) are defined based on the distinctly different community succession rates. (b) Principal coordinates analysis (PCoA) plots based on bacterial 16S rRNA gene sequences, showing the distinct community clusters across different seep development stages and years. Each point represents an individual sample, with the same color code shown in Fig. 3a. Adonis R and *P*-values indicate the proportion of variation explained by seep development stage (**P < 0.01). Similar trends for archaeal communities are shown in Fig. S8. (c) Variations in relative sequence abundances of dominant aerobic and anaerobic methanotrophic taxa across different seep development stages and years (upper and middle panel). The growth rates of different microbial lineages during Stage I and Stage II, respectively, were estimated as the log2-fold changes of 16S rRNA gene copies over time (lower panel). The symbol size is proportional to the growth rate, while symbol color indicates the average 16S rRNA gene abundances during Stage I or Stage II. (d) Integration of measurements on >40 sediment cores sampled around the discharge center over 2019–2023, revealing the spatial gradient of methane concentrations and relative abundances of ANME (mainly ANME-3 and ANME-2e) and aerobic methanotrophs (AeME, mainly *Methylomonadaceae* and *Methylophagaceae*). Methane concentrations were log_10_(x + 1) transformed to better visualize the low-range values. Spearman correlations were performed to examine correlations of methane concentrations with the relative abundances of ANME-3 and AeME (**P < 0.01).

Microbial community analysis revealed a rapid, directed succession towards a methanotroph-dominated ecosystem after the onset of methane seepage (Fig. 3). Pronounced shifts in dominant microbial taxa were observed even at class or phylum level: from bacterial *Alphaproteobacteria, Dehalococcoidia*, and *Anaerolineae*, and archaeal *Nitrososphaeria, Nanoarchaeia*, and *Bathyarchaeia* that often predominate in marine non-seep sediments [46–48], to seep-associated lineages such as bacterial *Gammaproteobacteria, Desulfobacteria, Bacteroidia*, and archaeal *Methanosarcinia* in the NBS sediments (Figs S6 and S7) [31,49]. Microbial community succession rates, quantified as the temporal changes of microbial community dissimilarity, were markedly high during 2019–2020 (Stage I: 0.2–0.3 yr^−1^, equivalent to a community turnover time of 3–5 years), but declined substantially during 2021–2023 (Stage II: <0.01 yr^−1^; Fig. 3a and b; Fig. S8). Matching this trend, the number of shared amplicon sequence variants (ASVs) between the non-seep and NBS communities decreased sharply during Stage I but rebounded slightly during Stage II. These trends indicate that microbial succession mostly occurred within the first 2–3 years after methane leakage. Although microbial community structures in the NBS sediments remained distinct from those in mature seeps (PERMANOVA, *P* < 0.01), they rapidly progressed toward the mature seep community type (Fig. 3b). Community structures of some samples from Stage II already resembled those from mature seeps (Fig. 3b; Fig. S8), indicating that the assembly of a typical mature seep microbiome can occur quickly within a few years.

Remarkably, the presumably ‘fast-growing’ aerobic methanotrophs and ‘slow-growing’ anaerobic methanotrophs proliferated synchronously since 2019, both reaching high abundances in 2022–2023 (Fig. 3c). The changes of absolute 16S rRNA gene abundances over time suggest that the dominant aerobic methanotrophic bacteria (*Methylomonadaceae*, up to 60% of bacterial sequences) and anaerobic methanotrophic archaea (up to 99% of archaeal sequences) both reached quasi-stationary phases of growth within 2–3 years (Fig. S9). The estimated *in situ* doubling time of *Methylomonadaceae* (mainly *Methyloprofundus*) was 80–107 days (Fig. 3c), matching the previous estimates of 60–120 days for aerobic methanotrophs in mud volcano sediments [32]. Unexpectedly, ANME-3 exhibited a notably shorter doubling time (24–38 days) during Stage I, suggesting an even faster initial response of anaerobic methanotrophs than aerobic methanotrophs to methane leakage. In addition, ANME-2e, a clade previously detected only sporadically by 16S rRNA or *mcrA* gene [50,51], responded even faster (doubling time: 21–36 days) than ANME-3, further demonstrating the pioneer-responding capacity of ANME to methane release (Fig. 3c, Fig. S10). These timescales are significantly shorter than the previously estimated doubling time of 100–200 days for ANME-3 in the newly erupted mud volcano sediments [32], as well as the model-based prediction of 60–100 years for establishing a steady-state, anaerobic methane-oxidizing community in response to new methane input [14,26,52], challenging the paradigm of anaerobic methanotroph’s delayed response to sudden methane input [14,26,53]. On the broader spatial scale surrounding the discharge center, methane concentration gradients correlated positively with the relative sequence abundances of aerobic and anaerobic methanotrophs (Spearman *P* < 0.01, Fig. 3d). It further suggests that except for the eruption center where gas bubbles rapidly escape into seawater, enhanced methane supply and transport through sediments directly amplify *in situ* responses of both aerobic and anaerobic methanotrophs.

Intriguingly, although ANME-2e and ANME-3 both rapidly proliferated from near-undetectable backgrounds following methane leakage, their abundances ultimately constituted only a minor fraction in mature seep sediments of the SCS where ANME-1 and ANME-2a/b predominated (Fig. 3c). This transient rise of specific ANME subgroups underscores the overlooked ecological plasticity of ANMEs and their potentially significant, stage-specific roles in modulating deep-sea methane cycling [31,49], offering a new successional framework for linking the different physiological traits of ANME subgroups to seep ecosystem development.

### Effective methane removal by animal-microbe synergy

**Figure 4.**
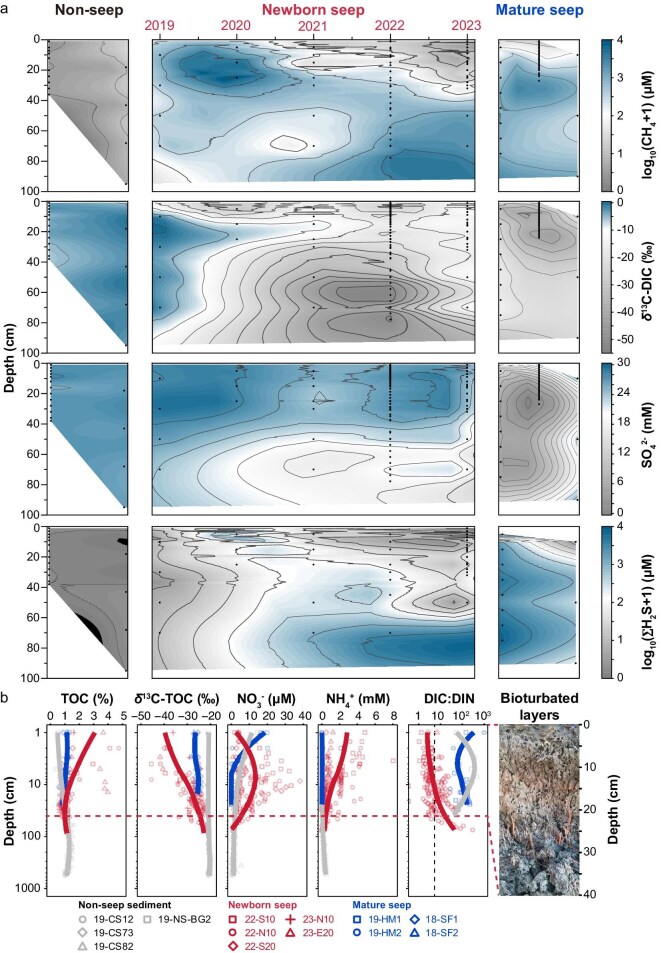
Time-series geochemical profiling reveals rapid methane removal and co-occurred active carbon, nitrogen, and sulfur cycling in the Newborn Seep sediments. (a) Contour plots of methane concentration (CH_4_), δ^13^C of dissolved inorganic carbon (δ^13^C-DIC), sulfate concentration (SO_4_^2−^), and total concentrations of hydrogen sulfide (∑H_2_S) in 0–100 cm sediments. Concentrations of CH_4_ and ∑H_2_S are log_10_(x + 1) transformed, to better visualize the wide ranges of concentration gradients. Black dots indicate the data points. (b) Depth profiles of total organic carbon (TOC), δ^13^C-TOC, nitrate (NO_3_^−^), ammonium (NH_4_^+^), ratios of dissolved inorganic carbon to nitrogen (DIC: DIN), and dissolved Fe^2+^ (log(x + 1) transformed) at the Newborn Seep, measured in 2022 and 2023 when the macrofauna bioturbation activities maximized. The symbols and lines represent the measured data and smooth fitting using the ‘loess’ function, respectively. The photograph on the right shows the sediment profile of a box core with dense faunal burrows.

Time-series geochemical profiling reveals an intricate biofilter formed jointly by the macro- and microbiota, which effectively consumed methane in both surface and subsurface sediments of the NBS (Fig. 4, Fig. S11). In 2019, elevated methane concentrations (10^2^–10^3^ μM) were detected within the 0–100 cm sediments, while isotopic signatures of dissolved inorganic carbon (δ^13^C-DIC), sulfate and ∑H_2_S concentrations remained at non-seep background levels, indicating the absence of methane-oxidizing activity in 2019 (Fig. 4a). Yet starting from 2021, in parallel with the increasing faunal and microbial population density, methane oxidation resulted in a sharp concentration decrease to 1–10 μM and a negative shift of δ^13^C-DIC (−10‰ to −30‰) in the upper 40–50 cm sediments (Fig. 4a). Likewise, rapid sulfide accumulation in 2020 and subsequent removal since 2021 strongly indicate the turnover of sulfide, particularly in the 0–30 cm sediments where dense faunal burrows were observed (Fig. 4a and b).

Within the strongly bioturbated layers, methane and sulfide oxidation apparently co-occurred with a network of other redox reactions. Elevated total organic carbon (TOC) with highly negative δ^13^C-TOC co-occurred with net consumption of oxygen, sulfate, and nitrate as quantified by the reaction-transport modeling, pointing to the potential coupling of methane oxidation to multiple electron acceptors (Fig. 4b, Table S2). The deep penetration of nitrate and notable accumulation of ammonium indicated highly active nitrate reduction. Isotopic labelling experiments confirmed this activity, further revealing rates of 4.55 ± 2.97 and 2.74 ± 1.78 mmol/m^2^/day for the coupling of nitrate reduction to methane and sulfide oxidation, respectively (Table S3). Notably, dissimilatory nitrate reduction to ammonium (DNRA) proceeded at markedly high rates (24.86 ± 1.06 mmol/m^2^/day), thereby supplying a large quantity of bioavailable nitrogen that likely promoted the seep ecosystem development. This finding sheds light on the long-standing enigma of how highly productive seep ecosystems emerge from nitrogen-poor subsurface geofluids [21]. Collectively, our results demonstrate that diverse redox reactions involving carbon, nitrogen, and sulfur co-occur within an expanded, intensely bioturbated depth horizon, defining a distinct and effective biofilter sustained by intricate animal-microbe interactions.

In contrast, in the deeper non-bioturbated layers (50–100 cm), a pronounced δ^13^C-DIC depletion (−55% to −20%) coincided with reduced sulfate concentrations (16–20 mM) and ∑H_2_S accumulation (10^2^–10^4^ μM) since 2021. Together the geochemical monitoring reveals an effective methane consumption throughout the top 100 cm of the NBS sediments across two distinct geochemical zones: (1) methane oxidation co-occurred with diverse redox reactions in the 0–50 cm sediments, likely promoted by bioturbation-induced redox reactions, and (2) anaerobic methane oxidation coupled with sulfate reduction in the 50–100 cm sediments. The integrated methane oxidation fluxes throughout 0–100 cm sediments of the NBS, based on the time-dependent reaction-transport modeling, were 38.2–48.0 mmol/m^2^/day, matching fluxes estimated using the Monod biomass-explicit model (31.5–62.2 mmol/m^2^/day, Table S2). This budget accounts for 60%–100% of the methane oxidation flux (51.5–53.3 mmol/m^2^/day) under steady-state mature seep settings with similar seepage rates, further supporting the rapid establishment of an efficient methane biofilter following methane leakage.

### Methane and sulfur oxidations fueled by multiple electron acceptors

**Figure 5.**
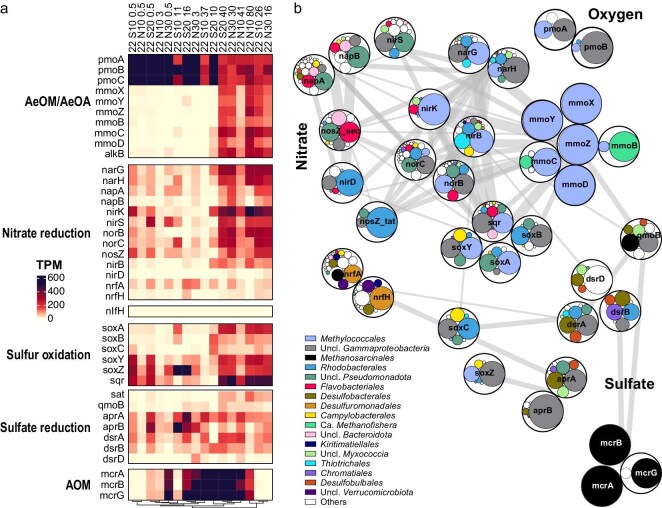
Metatranscriptomic analyses reveal methane and sulfur oxidations fueled by multiple electron acceptors at the Newborn Seep. (a) Heatmaps showing the transcript abundances of key functional genes involved in aerobic methane/alkane oxidation (AeOM/AeOA), nitrate reduction, sulfur oxidation, sulfate reduction, and anaerobic methane oxidation (AOM). The color scale indicates transcripts per million (TPM), with darker colors reflecting higher transcription levels. Each column represents a sample, while each row corresponds to a specific gene or gene set. Nomenclature of sample names: sampling year_sampling site_sediment depth. (b) Gene co-transcription network linking major metabolic functions and microbial taxa. Each node (outer circle) represents a gene and each edge denotes significant correlations (Spearman *P* < 0.05). Within each outer circle, the inner circles indicate the taxonomic composition associated with each gene. The network underscores the potential coupling of methane oxidation with the reductions of dioxygen, nitrate, and sulfate, as well as the coupling of sulfur oxidation with nitrate reduction at the Newborn Seep. Effects of different types of microplastics on the growth.

Targeted metatranscriptomic analyses performed during the period with intense bioturbation (2022–2023) confirm that methane and sulfur oxidations were supported by a diverse suite of electron acceptors, highlighting the ecological plasticity of seabed communities to rapidly respond and adapt to chemical perturbations (Fig. 5). Transcript abundances of aerobic methane oxidation genes (*pmoABC*) were high in all of the NBS samples examined, particularly in the 0–20 cm layers (transcripts per million (TPM) > 600, Fig. 5a). Notably, high transcript abundances of *pmoABC* and the AOM genes *mcrABG* co-occurred in 9 out of the 16 transcriptomes analyzed, even in the 0–20 cm sediments, matching the co-existence of aerobic and anaerobic methanotrophic clades in >85% of the NBS samples analyzed. This is most likely related to the dense faunal populations, which created spatiotemporal mosaics of oxic-anoxic microenvironments that facilitated the co-occurrence of various aerobic and anaerobic processes [47,54,55]. Intriguingly, another set of aerobic methane oxidation genes, *mmoXYZBCD*, was transcribed concurrently with nitrate reduction genes (*narGH, nirBK, norBC*) and sulfur oxidation genes (*sqr, sox*) mainly in the 20–50 cm sediment layers (TPM 70–300)—these genes thus were strongly correlated (all with Spearman *r* > 0.8, *P* < 0.01; Fig. 5b). Taxonomic annotation on transcripts of these genes attributed them to *Methylococcales* (mainly *Methylomonadaceae*), indicating the potential coupling of methane, nitrogen, and sulfur cycling mediated by *Methylomonadaceae*. Collectively, it appears that the reductions of diverse electron acceptors (O_2_, NO_3_^−^, SO_4_^2−^) generally co-occurred and jointly fueled the high rates of methane oxidation in surface sediments of the NBS.

### Implications

Our findings demonstrate that abrupt methane leakage triggers a rapid transformation of the seabed ecosystem, simultaneously causing a reduction in seabed microbial diversity and the swift *de novo* assembly of an effective ‘methane biofilter.’ The established correlations between seabed microbial diversity and *in situ* concentrations of methane and hydrogen sulfide suggest that even micromolar-level increases in these compounds are indicative of marked biodiversity decreases, offering a potential benchmark for assessing the ecological tipping point and risks in the hydrate-rich seabed. Contrary to the paradigm of inherently slow deep-sea metabolisms [56,57], we show that certain microbial and faunal guilds can exploit sudden energy inputs with remarkable speed. Most notably, the anaerobic methanotrophs ANME-2e and ANME-3 responded even faster than their aerobic counterparts. The pioneer response and transitory dominance of ANME-2e and ANME-3 underscore the overlooked ecological plasticity of ANME, a trait warranting deeper physiological and metabolic investigations [58]. Continuous monitoring of this model ecosystem will reveal how the dominance of pioneering microbial and macrofaunal responders progressively shifts toward typical mature seep communities while maintaining chemosynthetic ecosystem functioning. These insights are necessary for closing key knowledge gaps regarding the formation, history, and evolution of marine cold seeps [19,27,29].

The rapid formation of this chemosynthetic ecosystem following methane leakage offers new perspectives on deep-sea biogeochemical cycles. The newly formed ecosystem demonstrates considerable methane oxidation capacity, reaching rates among the highest recorded at natural seeps worldwide within just a few years [22,23]. Such methane-filtering capacity is largely sustained by the rapidly established animal-microbe synergies, in which opportunistic polychaetes maintain redox gradients and fluctuations that facilitate the coupling of microbial methane oxidation with various redox reactions. It suggests that deep-sea organisms can rapidly coordinate to establish an integrated network of highly accelerated carbon, nitrogen, and sulfur cycling. These findings necessitate the incorporation of animal-microbe synergy into the prevailing microbial-centric models, which predicted a ‘window’ of 60–100 years for substantial methane release before the establishment of an efficient, steady-state microbial biofilter [14,23,26,53]. The new perspectives on the timescale and nature of seabed responses to methane leakage are imperative as thousands of offshore gas and oil wells worldwide show high rates (10%–43%) of integrity failures that pose methane leakage risks [36,37], and as subsurface methane reservoirs are increasingly destabilized under climate- and human-induced perturbations [6–9]. These insights are crucial for refining current models and predictions of how seabed ecosystems, and their vital role in mitigating methane and other hydrocarbon releases, will respond to escalating stressors from climate change and expanding industrial activity in the deep ocean [8,34,35].

## MATERIALS AND METHODS

Detailed methods are described in the Supplementary data Text S1.

### 3D seismic survey

High-resolution 3D seismic survey was conducted in the Songnan Low uplift of Qiongdongnan Basin, SCS, to identify the seismic response characteristics associated with subseafloor geological structure, fluid migration, and gas hydrate accumulation. The data were acquired using 12 parallel streamers with 100 m spacing, with inline (NW-SE) and crossline (SW-NE) spacings of 12.5 m and 12.5 m, respectively, and a 1.0 ms sampling interval.

### Multibeam echosounder survey

Annual hydroacoustic surveys were conducted to track the activity of methane discharge from seafloor in the study region. Acoustic backscatter data from mid-water reflectors were collected using a shipborne Kongsberg EM302 multibeam echosounder, operating at a nominal frequency of 30 kHz with a swath width of 120°.

### Sediment and porewater sampling

Sediment samples were collected from three major types of habitats in the SCS: the ‘Newborn Seep (NBS),’ mature cold seeps (S18, ‘Haima,’ and ‘Site F’), and non-seep continental slope sites (SCS12, SCS73, SCS82) (Fig. 1, Table S1). Specifically, sediments were sampled from >40 sites around the discharge center of the NBS during 2019–2023 (Fig. S1). Two non-seep background sites 19-NS-BG1 and 19-NS-BG2 were sampled ∼200 m away from the discharge center in 2019, before the spreading of leakage impact on seafloor. Sediments were sampled primarily using push-core samplers of the remotely operated vessel ‘Haima,’ compensated by piston core and box core sampling for subsurface geochemical profiling and macrofauna analysis, respectively. Porewater was extracted from sediment cores at different depths using the Rhizon sampler (0.15 μm pore size, Rhizosphere, Netherlands). Sediment cores were subsequently sliced into layers every 2–5 cm (Table S1). All samples were stored in proper temperatures and conditions for different analytical purposes (Table S4).

### Macrofaunal analyses

Macrofaunal identification and quantification were performed using a sediment sieving-based method (0.5-mm mesh size). Only few copepods can be retrieved by the sieving method despite their relatively high abundances recorded by underwater camera. The abundance of these faunal species therefore was estimated by analyses on images/videos that clearly visualized the individuals of copepods.

### Geochemical analyses

Methane concentrations were measured using a gas chromatograph (Shimadzu) equipped with a Barrier Discharge Ionization Detector. DIC concentration and its carbon isotopic ratio (δ^13^C_DIC_) were determined using Gas Bench II coupled with a Stable Isotope Ratio Mass Spectrometer (Delta 253plus, Thermo Scientific). Hydrogen sulfide was measured by spectrophotometry (DR5000, Hach), while sulfate was quantified by ion chromatography (Dionex ICS-5000+, Thermo Scientific). NO_3_^−^, NO_2_^−^ and NH_4_^+^ were measured using a continuous flow Auto Analyzer (AA3, SEAL). TOC, TN, and δ^13^C-TOC were determined after removing inorganic carbon through acidification, using an elemental analyzer (Vario EL III) coupled to an isotope ratio mass spectrometer (Isoprime, Elementar).

### Time-dependent reaction-transport model

To model the reaction rates and fluxes of dissolved chemical species in sediments, the evolution of porewater profiles over time was simulated using a 1D time-dependent reaction transport model:


\begin{eqnarray*}
\frac{{\partial \phi C}}{{\partial t}} &=& - \frac{\partial }{{\partial x}}\left( {\phi D\frac{{\partial C}}{{\partial x}} - \phi uC} \right)\\
&&+\, \alpha \phi \left( {{C}_0 - C} \right) + \Sigma R,
\end{eqnarray*}

where *C* is the solute concentration (M L^−3^_fluid_), *t* is time, $\phi $ is porosity, *D* is the tortuosity-corrected diffusion coefficient, *u* is the advection velocity, *α* is a nonlocal transport coefficient, *C*_0_ is the concentration in the overlying water, and *ΣR* is the net rate of production or consumption of chemical species *C* (M L^−3^_total_ T^−1^). See the supplementary texts for detailed settings of the reaction networks and boundary conditions.

### Monod biomass-explicit model

Methane oxidation rates were also quantified using the Monod biomass-explicit model, assuming the constant coupling between cell growth rate and specific substrate oxidation rate (i.e. there is no temporary uncoupling or ‘unproductive’ oxidation) [24]:


\begin{eqnarray*}
V = u \cdot {Y}_{mol}^{ - 1},
\end{eqnarray*}

where *V* is the specific rate of substrate oxidation (mol substrate oxidized per cell dry weight per time). *u* is the net cell growth rate, estimated based on the temporal changes in abundances of specific methanotrophic lineages. ${Y}_{mol}\ $is the molar growth yield, was set to 0.6 g cell dry weight per mol CH_4_ oxidized according to the experiment data of typical methane seep sediments [24].

### Isotopic labeling experiments

Potential rates of denitrification and DNRA were measured using ^15^N-labeling slurry incubations. The rates of denitrification and DNRA were calculated from ^30^N_2_ and ^15^NH_4_^+^ generations within the vials, respectively, which were measured by the membrane inlet mass spectrometry and by a micro-diffusion approach. The coupled rates of nitrate reduction to methane and sulfide oxidation were calculated by comparing the treatments of NO_3_^−^+CH_4_ and NO_3_^−^+S^2−^ with control (NO_3_^−^), respectively.

### Nucleic acids extraction and 16S rRNA gene sequencing

Genomic DNA was extracted from 0.5–2.0 g of sediments using a modified SDS-based extraction method [45]. After library preparation and 16S rRNA gene sequencing on the Illumina Miseq platform, sequence reads were processed following the procedures of trimming, quality filtering, denoising, paired-end sequence merging, chimera filtering, ASVs production, and taxonomic annotation. Multiple sequence alignment and phylogenetic tree construction were performed using the QIIME 2 plugin q2phylogeny (align-to-tree-mafft-iqtree). Unassigned sequences, singletons and sequences affiliated with eukaryotes were discarded.

### Metatranscriptomic analysis

RNA was extracted from ∼5 g of sediments using the RNeasy PowerSoil Total RNA Kit (QIAGEN, Germany). After removing ribosomal RNA and DNA, libraries were prepared and sequenced on the Illumina NovaSeq-PE150 platform. After quality filtration of raw metatranscriptomic reads, mRNA sequences were obtained by removing rRNA sequences. Sam files were generated by mapping mRNA sequences to metagenome-assembled genomes. Gene transcription was calculated by counting the number of unambiguously mapped reads for each gene. To compare transcription levels between genes, read counts were converted to TPM. A metabolic network at the transcriptional level was constructed based on the pairwise Spearman correlations between gene transcripts. Only correlations with *P* < 0.01 were shown in the network. The taxonomic information of each gene was further annotated with the NR database using diamond blastp.

### Quantitative PCR

Abundances of bacterial and archaeal 16S rRNA genes were quantified using the SYBR-Green I-based quantitative PCR (qPCR) assay. Details of the used primers, reaction mixture recipe, thermal cycling program, and prokaryotic standards are listed in supplementary texts. Microbial cell abundance was subsequently estimated using the average 16S gene copies per cell (Bacteria: 4.12 ± 2.75 copies cell^−1^; Archaea: 1.61 ± 0.88 copies cell^−1^) based on the rrn database (https://rrndb.umms.med.umich.edu/).

### *In situ* cell growth

The net cell doubling time (DT) was estimated as the quotient of time and log_2_-fold changes in 16S rRNA gene copies of specific lineages:


\begin{eqnarray*}
{\mathrm{DT}} = \frac{{{\mathrm{t}}_1 - {\mathrm{t}}_0}}{{{{\log }}_2\frac{{{{\mathrm{N}}}_{{\mathrm{t}}1} \times {{\mathrm{f}}}_{{\mathrm{t}}1}}}{{{{\mathrm{N}}}_{{\mathrm{t}}0} \times {{\mathrm{f}}}_{{\mathrm{t}}0}}}}},
\end{eqnarray*}

where t_1_ is the specific time point after the onset of methane seepage at t_0_. N is the total 16S rRNA gene copy number based on qPCR, f is the fraction (i.e. relative abundance) of specific lineage based on the 16S rRNA gene sequencing data. For N and f at t_0_, data retrieved from the non-seep background sites were used to represent the community status before methane leakage. The net cell growth rates (R) were further calculated as:${\mathrm{R}} = \frac{{\ln 2}}{{{\mathrm{DT}}}}.$

### Statistics

All statistical analyses were performed in R (https://www.r-project.org). Microbial alpha-diversity indices and NMDS coordinates were calculated using the ‘phyloseq’ package. Using the ‘vegan’ package, we performed: (1) PERMANOVA analysis to examine the community (dis)similarity between different groups of samples, (2) two-sided Welch’s *t* test with Bonferroni-Holm correction to compared means between two independent groups. The succession rates of microbial communities were determined by calculating the temporal variations of the community distances measured by both Bray-Curtis and Sorensen metrics with time. The potential contribution of methane-derived carbon to the faunal diet was calculated by the ‘MixSIAR’ package, using the default uninformative prior and Markov chain Monte Carlo settings.

## Supplementary Material

nwag266_Supplemental_Files

## Data Availability

Sequencing data are available online through the NCBI database under the project number PRJNA1314185 (16S rRNA gene sequences) and PRJNA1313825 (metatranscriptomes). All other data needed to evaluate the conclusions of this paper and the custom codes for numerical modeling can be found in the main text or Supplementary Data.
