# Supplementary material for: Rapid de novo assembly of animal-microbe biofilter to mitigate seabed methane leakage
Source: Natl Sci Rev. 2026 May 14;13(12):nwag266. doi: 10.1093/nsr/nwag266 (PMC13296563; doi:10.1093/nsr/nwag266)
Supplement: nwag266_Supplemental_Files [file nwag266_supplemental_files.zip › Code S1.pdf]

```
# early diagenesis model including acid-base chemistry
# implementation for Longhui's new cold seep, Fall 2024
# based on the models by Jurjen, Kadir, Maruf

# solves d(eta*C)/dt = - d(A*J)/dx + R
# with
# eta = porosity (solutes) or 1-porosity (solids)
# A: cross sectional area (1)
# J =    −eta*D · dC/dx + eta*u · C [u: (pore scale) velocity; D: diffusion coeff]
# R: net reaction rate of chemical C in M/L3tot/T
# solutes are in M/L3fluid, solids in M./L3solid
# SI Units: Mass =    mol, Space = m, Time = s --> C in mmol/L = mol/m3

library(marelac) # constants
library(ReacTran) # reactive transport framework
library(AquaEnv) # acid-base
library(readxl)
library(patchwork)
library(ggplot2)
library(ggpubr)

rm(list=ls())
graphics.off()
t0=Sys.time() # to calculate time cost

## Working directory
#setwd("/Users/cmeile/Documents/Matlab_apps/longhui")
measured_nonseep <- read_excel("New seep_2024_Christof-2.xlsx", sheet = "NonSeep")
measured_2019 <- read_excel("New seep_2024_Christof-2.xlsx", sheet = "NewSeep2019")
measured_2020 <- read_excel("New seep_2024_Christof-2.xlsx", sheet = "NewSeep2020")
measured_2021 <- read_excel("New seep_2024_Christof-2.xlsx", sheet = "NewSeep2021")
measured_2022 <- read_excel("New seep_2024_Christof-2.xlsx", sheet = "NewSeep2022")
measured_2023 <- read_excel("New seep_2024_Christof-2.xlsx", sheet = "NewSeep2023")
measured_mature1 <- read_excel("New seep_2024_Christof-2.xlsx", sheet = "MatureSeep1")
measured_mature2 <- read_excel("New seep_2024_Christof-2.xlsx", sheet = "MatureSeep2")

# Plot function
plot_compare <- function(s.x, s.y, nsol=1, m.x, m.y, ylab=NULL, title=NULL,
                         legend.title=NULL, sol.lab=seq(nsol)) {
  run <- factor(rep(1:nsol, each=length(s.y)/nsol))
  p <- ggplot() +
    geom_point(aes(x=m.x, y=m.y)) +
    geom_line(aes(x=s.x, y=s.y, col=run, lty=run)) +
    ylim(0, NA) +
```

```
       scale_x_reverse() +
       coord_flip() +
       labs(x="Depth (m)", y=ylab, title = title, col=legend.title,
            lty=legend.title) +
       scale_color_discrete(labels=sol.lab) +
       scale_linetype_discrete(labels=sol.lab) +
       theme(text = element_text(size=20)) +
       theme_bw()
   return(p)
}

# args = commandArgs(trailingOnly=T)
# args = list(RC0 = 200, fe_flux = 1, Dbml_depth = 3, mineral = 'calcite')
# test if there is at least 4 argument: if not, return an error
#if (length(args) < 4) {
#   stop("Give following parameters in order: RC0 (numeric), Fe Flux (mol cm2 yr-1), mixed
layer depth (numeric, cm),
#            mineral type (character)",
#            call.=FALSE)
#}

#### Model domain and grid definition ####
L <- 1.5       # depth of sediment domain [cm] to [m]

N <- 601 # 2*4*125     # number of grid layers
grid <- setup.grid.1D(x.up = 0, L = L, N = N)   # generate constant 1D grid

secperyr <- 365*24*60^2     # seconds per year

# transient simulations
tstart <- 0 # start time
tend <- (5+5)*secperyr # end time (sec). 5 years are to just reach the surface, then another 5
yrs
dtout <- 1*secperyr # timestep for output
times <- seq(tstart, tend, by=dtout)

# A handy function for plotting profiles
plot_profile <- function(..., ylab = ylab) {
   plot(y = grid$x.mid, ylim = c(L,0), type = "l",
         ..., ylab = "depth (m)")
}

##### Environmental parameters -----
S <- 36.3             # salinity
```

```
TC <- 5                  # temperature [deg C]
Pbar <- 1.013       # pressure [bar]
rho_sw <- as.numeric(aquaenv(S = S, t = TC, P = Pbar)$density) # density in kg/m3

por.0 <- 0.67     # porosity at top
por.inf <- 0.55 # porosity at infinite depth
gamma <- 0.05       # decay in 1/cm. e-folding distance (Rooze et al., 2016)
por <- por.inf + (por.0 - por.inf) * exp(-grid$x.mid/gamma) # por as function of depth (Jurjens paper has this as e^ -x/gamma)
tort <-   1 - (2 * log(por))         # tortuosity coefficient (Rooze et al., 2016)
plot_profile(x = por, main = "por", ylab = "depth (m)")   # show porosity profile

##### tortuosity corrected Diffusion coefficient in m2/s ----
# uses routine 'diffcoeff' from 'marelac' package
Dmol.O2   <- diffcoeff(S = S, t = TC, P = Pbar, species = "O2")$O2 / tort
Dmol.SO4 <- diffcoeff(S = S, t = TC, P = Pbar, species = "SO4")$SO4 / tort
Dmol.NO3 <- diffcoeff(S = S, t = TC, P = Pbar, species = "NO3")$NO3 / tort
Dmol.NH4 <- diffcoeff(S = S, t = TC, P = Pbar, species = "NH4")$NH4 / tort
Dmol.TS   <- diffcoeff(S = S, t = TC, P = Pbar, species = "HS")$HS / tort # HS- for TS
Dmol.DIC <- diffcoeff(S = S, t = TC, P = Pbar, species = "HCO3")$HCO3 / tort # HCO3- for DIC
Dmol.TA   <- diffcoeff(S = S, t = TC, P = Pbar, species = "HCO3")$HCO3 / tort # HCO3- for TA
Dmol.CH4 <- diffcoeff(S = S, t = TC, P = Pbar, species = "CH4")$CH4 / tort

##### advection --------------------------------------------
v.inf <- 0.002/secperyr # sedimentation rate solids ~ 2mm/yr [m/s]
v <- v.inf * (1 - por.inf) / (1 - por) # solid burial velocities corrected for compaction
u.inf <- v.inf # sedimentation rates solutes
ucompaction <- u.inf * por.inf / por #solute burial (pore) velocities corrected for compaction
useepage <- -0.3/secperyr # seepage rate ~  -0.3 m/yr [m/s]
iseepage <- 0 # 0: no seepage, 1: seepage, imposing flux, 2: seepage, imposing conc. (overwritten below)
u <- ucompaction


##### bioirrigation --------------------------------------------
alpha0 <- 200/secperyr # 140/yr [1/s]
alphaml_depth = 0.03 # mixed layer depth [m]
alpha_efold <- 0.075 # decay of irrigation coefficient with depth [m]
alpha1 <- setup.prop.1D(func = p.exp,
                        grid = grid,
                        y.0 = alpha0, y.inf = 0, x.L = alphaml_depth, x.att = alpha_efold) #y.0 = 10 (Owings et al., 2021)
```

```
alpha <- alpha1$mid
plot_profile(x = alpha, main = "alpha", ylab = "depth (m)")

##### bioturbation -------------------------------------------
Db0 = 0.0024/secperyr # [m2/s]
Dbml_depth = 0.03 # mixed layer depth [m]
Db1 <- setup.prop.1D(func = p.exp,
                     grid = grid,
                     y.0 = Db0, y.inf = 0, x.L = Dbml_depth, x.att = 0.0075) #y.0 = 10
(Owings et al., 2021)
Db <- Db1$mid
plot_profile(x = Db, main = "Db", ylab = "depth (m)")

##### Boundary conditions (bottom-water) (mol/m3 = mmol/L) ----
# aquaenv: input and output in mol/kg. convert output to mmol/L
TA.ow <- 2.44 # meq/L
pH.ow <- 8.2
AE.ow <- aquaenv(S = S, t = TC, P = Pbar, pH = pH.ow, TA = TA.ow/rho_sw, SumCO2 = NULL)
DIC.ow <- AE.ow$SumCO2 * rho_sw
SO4.ow <- 29
O2.ow <- 0.25
TS.ow <- 0.0
NO3.ow <- 0.04
NH4.ow <- 0.0
CH4.ow <- 0

# composition of seepage fluid @ depth
TA.seep <- 2.35 #4.44 # meq/L
pH.seep <- 7.78 #8.0
AE.seep <- aquaenv(S = S, t = TC, P = Pbar, pH = pH.seep, TA = TA.seep/rho_sw, SumCO2
= NULL)
DIC.seep <- AE.seep$SumCO2 * rho_sw
SO4.seep <- 0*29.86
O2.seep <- 0.
TS.seep <- 0.86/1000.
NO3.seep <- 0.
NH4.seep <- 20.2/1000.
CH4.seep <- 50. #10.

##### Biogeochemical rate parameters ----

###### organic matter mineralization ----
a <- 1              # composition of OM: C/OM
b <- 16/106         # composition of OM: N/C
```

```
c <- 1/106
RC0 <- 60./secperyr/100      # OM mineralization in [molC/m3/s] 60 mol/m3/yr -> 60, 657 120
betaOM <- 0.12              # 1/m
RCml_depth <- 0.03      # m
KmOM.O2 <- 2e-2           # Half-sat constant for O2 in OM mineralization in mM
KmOMdnf.NO3 <- 2e-3     # Half-sat constant for NO3 in OM mineralization via DNF
KmOMdnra.NO3 <- 2e-3   # Half-sat constant for NO3 in OM mineralization via DNRA
KmOM.SO4 <-     1.6        # Half-sat constant for SO4 in OM mineralization in mM
fdnf <- 0.3
RC1 <- setup.prop.1D(func = p.exp,
                     grid = grid,
                     y.0 = RC0, y.inf = 0, x.L = RCml_depth, x.att = betaOM) #y.0 = 10 (Owings et al., 2021)
RC <- RC1$mid
plot_profile(x = RC, main = "RC", ylab = "depth (m)")

###### secondary reactions ----Re-evaluated
ktso2 <- 1.6e2/secperyr     #/(mol/m3)/s - O2 * TS [m3/mol/yr] (Rooze et al., 2016)
knh4o2 <- 5e3/secperyr       #/(mol/m3)/s - NH4 with O2 (Rooze et al., 2016)
kch4o2 <- 1e10/1000/secperyr # m3/mol/s.   #/(mol/m3)/s - CH4 with O2 (Wang and Van Cappellen et al., 1996)
kch4so4 <- 1e7/1000/secperyr # m3/mol/s.            #/(mol/m3)/s - CH4 with SO4 (Iversen & Jorgensen, 1985, From W&V.C 1996)
kch4no3 <- 5e3/secperyr          # m3/mol/s. # F. Lopes et al. / Applied Geochemistry 26 (2011) 1919–1932
kno3ts <- 5e3/secperyr       #/(mol/m3)/s - sulfide with nitrate

vAFDW <- fiadeiro(v = useepage, D = 1e-10, dx.aux =grid$dx.aux)

parms <- c(iseepage = iseepage,
           alpha = alpha,
           Db = Db,
           u = u,
           RC = RC)

#### Model formulation ####

ED.model <- function (t, state, parms) {
  with(as.list(c(state, parms)), {

    # Initialization of state variables
    O2 <- state[1:N]
    SO4 <- state[(N+1):(2*N)]
```

```
NO3 <- state[(2*N+1):(3*N)]
NH4 <- state[(3*N+1):(4*N)]
TS <- state[(4*N+1):(5*N)]
DIC <- state[(5*N+1):(6*N)]
TA <- state[(6*N+1):(7*N)]
CH4 <- state [(7*N+1):(8*N)]

# Transport terms
if(iseepage == 0){
  # no seepage -> no gradient for solutes
  tranO2 <- tran.1D(C = O2, C.up = O2.ow, D = Dmol.O2, v = u, VF = por, dx = grid, AFDW =vAFDW)
  tranSO4 <- tran.1D(C = SO4, C.up = SO4.ow, D = Dmol.SO4, v = u, VF = por, dx = grid, AFDW =vAFDW)
  tranNO3 <- tran.1D(C = NO3, C.up = NO3.ow, D = Dmol.NO3, v = u, VF = por, dx = grid, AFDW =vAFDW)
  tranNH4 <- tran.1D(C = NH4, C.up = NH4.ow, D = Dmol.NH4, v = u, VF = por, dx = grid, AFDW =vAFDW)
  tranTS <- tran.1D(C = TS, C.up = TS.ow, D = Dmol.TS, v = u, VF = por, dx = grid, AFDW =vAFDW)
  tranDIC <- tran.1D(C = DIC, C.up = DIC.ow, D = Dmol.DIC, v = u, VF = por, dx = grid, AFDW =vAFDW)
  tranTA <- tran.1D(C = TA, C.up = TA.ow, D = Dmol.TA, v = u, VF = por, dx = grid, AFDW =vAFDW)
  tranCH4 <- tran.1D(C = CH4, C.up = CH4.ow, D = Dmol.CH4, v = u, VF = por, dx = grid, AFDW =vAFDW) #Newly_added_for_starters
}
if(iseepage == 1) {
  # seepage -> flux from below for solutes
  tranO2 <- tran.1D(C = O2, C.up = O2.ow, flux.down = u[N]*por[N]*O2.seep, D = Dmol.O2, v = u, VF = por, dx = grid, AFDW =vAFDW)
  tranSO4 <- tran.1D(C = SO4, C.up = SO4.ow, flux.down = u[N]*por[N]*SO4.seep, D = Dmol.SO4, v = u, VF = por, dx = grid, AFDW =vAFDW)
  tranNO3 <- tran.1D(C = NO3, C.up = NO3.ow, flux.down = u[N]*por[N]*NO3.seep, D = Dmol.NO3, v = u, VF = por, dx = grid, AFDW =vAFDW)
  tranNH4 <- tran.1D(C = NH4, C.up = NH4.ow, flux.down = u[N]*por[N]*NH4.seep, D = Dmol.NH4, v = u, VF = por, dx = grid, AFDW =vAFDW)
  tranTS <- tran.1D(C = TS, C.up = TS.ow, flux.down = u[N]*por[N]*TS.seep, D = Dmol.TS, v = u, VF = por, dx = grid, AFDW =vAFDW)
  tranDIC <- tran.1D(C = DIC, C.up = DIC.ow, flux.down = u[N]*por[N]*DIC.seep, D = Dmol.DIC, v = u, VF = por, dx = grid, AFDW =vAFDW)
  tranTA <- tran.1D(C = TA, C.up = TA.ow, flux.down = u[N]*por[N]*TA.seep, D = Dmol.TA, v = u, VF = por, dx = grid, AFDW =vAFDW)
  tranCH4 <- tran.1D(C = CH4, C.up = CH4.ow, flux.down = u[N]*por[N]*CH4.seep, D =
```

```
Dmol.CH4, v = u, VF = por, dx = grid, AFDW =vAFDW)
    }
    if(iseepage == 2) {
      # seepage -> concentration of seep fluid at the bottom boundary
      tranO2 <- tran.1D(C = O2, C.up = O2.ow, C.down = O2.seep, D = Dmol.O2, v = u, VF
= por, dx = grid, AFDW =vAFDW)
      tranSO4 <- tran.1D(C = SO4, C.up = SO4.ow, C.down = SO4.seep, D = Dmol.SO4, v =
u, VF = por, dx = grid, AFDW =vAFDW)
      tranNO3 <- tran.1D(C = NO3, C.up = NO3.ow, C.down = NO3.seep, D = Dmol.NO3,
v = u, VF = por, dx = grid, AFDW =vAFDW)
      tranNH4 <- tran.1D(C = NH4, C.up = NH4.ow, C.down = NH4.seep, D = Dmol.NH4, v
= u, VF = por, dx = grid, AFDW =vAFDW)
      tranTS <- tran.1D(C = TS, C.up = TS.ow, C.down = TS.seep, D = Dmol.TS, v = u, VF =
por, dx = grid, AFDW =vAFDW)
      tranDIC <- tran.1D(C = DIC, C.up = DIC.ow, C.down = DIC.seep, D = Dmol.DIC, v = u,
VF = por, dx = grid, AFDW =vAFDW)
      tranTA <- tran.1D(C = TA, C.up = TA.ow, C.down = TA.seep, D = Dmol.TA, v = u, VF
= por, dx = grid, AFDW =vAFDW)
      tranCH4 <- tran.1D(C = CH4, C.up = CH4.ow, C.down = CH4.seep, D = Dmol.CH4, v
= u, VF = por, dx = grid, AFDW =vAFDW)
    }

    # reactions   --------------------------------

    # mineralization in molC/m3_tot/s
    #RC <- RC0 * exp(-betaOM*grid$x.mid)
    # (CH2O)a(NH3)b(H3PO4)c +a O2 + (b -2c)H+ = a CO2 + b NH4+ + c HPO42- + a H2O
#Newly_added_for_starters
    ROM.O2 = RC * pmax(O2,0) / (KmOM.O2 + pmax(O2,0))
    # (CH2O)a(NH3)b(H3PO4)c + a/2 NO3- + a/2 H20 + (+b -2c) H+ = 2a/5 N2 + a HCO3
+ (b) NH4+ + c HPO42- + 2a/5H2O   #Newly_added_for_starters
    ROM.NO3dnf = (RC - ROM.O2) * pmax(NO3,0) / (KmOMdnf.NO3 + pmax(NO3,0))*fdnf
    # (CH2O)a(NH3)b(H3PO4)c + a/2 NO3- + a/2 H20 + (+b -2c) H+ = 2a/5 N2 + a HCO3
+ (b) NH4+ + c HPO42- + 2a/5H2O   #Newly_added_for_starters
    ROM.NO3dnra = (RC - ROM.O2) * pmax(NO3,0) / (KmOMdnra.NO3 + pmax(NO3,0))*(1-
fdnf)
    # (CH2O)a(NH3)b(H3PO4)c + a/2 SO42- + (a/2 +b -2c) H+ = a CO2 + b NH4+ + c
HPO42- + a/2 HS- + a H2O #Newly_added_for_starters
    ROM.SO4 = (RC - ROM.O2 - ROM.NO3dnf - ROM.NO3dnra) * pmax(SO4,0) /
(KmOM.SO4 + pmax(SO4,0))
    # (CH2O)x (NH3)y (H3PO4)z + x/2 H2O +    x/2 H+   →x/2CH4 + x/2HCO3- + yNH4+
+ zHPO42- + (-x+y-2z)H+ #Newly_added_for_starters
    ROM.CH4 = RC - ROM.O2 - ROM.NO3dnf - ROM.NO3dnra - ROM.SO4
```

```r
# reoxidation in molX/m3pw/s
# H2S + 2O2 = SO4 + 2H+
Rtso2 <- ktso2 * pmax(O2,0) * pmax(TS,0)
# NH4+ + 2O2 = NO3- + 2H+ + H2O
Rnh4o2 <- knh4o2 * pmax(O2,0) * pmax(NH4,0)
# CH4 + 2O2      = CO2 + 2H2O
Rch4o2 <- kch4o2 * pmax(CH4,0) * pmax(O2,0)
# CH4 + CO2 + SO4      =    2HCO3 + H2S
Rch4so4 <- kch4so4 * pmax(CH4,0) * pmax(SO4,0)
# NO3- + HS- + H+ + H2O = NH4+ + SO4
Rno3ts <- kno3ts * pmax(NO3,0) * pmax(TS,0)
# CH4 + 1.6NO3- + 0.6H+ = HCO3 + 0.8N2 + 1.8H2O
Rch4no3 <- kch4no3 * pmax(NO3,0) * pmax(CH4,0)

# assemble net rates in mol/m3_tot/yr
R.O2 <- -a*ROM.O2 + (-2*Rtso2 -2*Rnh4o2 -2*Rch4o2)*por
R.SO4 <- -a/2*ROM.SO4 + (Rtso2 -Rch4so4 +Rno3ts)*por
R.NO3 <- -4*a/5*ROM.NO3dnf -a/2*ROM.NO3dnra + (Rnh4o2 -Rno3ts)*por - 1.6*Rch4no3*por
R.NH4 <- b*ROM.O2 + b*ROM.NO3dnf + (a/2+b)*ROM.NO3dnra + b*ROM.SO4 + b*ROM.CH4 + (-Rnh4o2 +Rno3ts)*por
R.TS <- a/2*ROM.SO4 + (-Rtso2 -Rno3ts +Rch4so4)*por
R.DIC <- a*ROM.O2 + a*ROM.NO3dnf + a*ROM.NO3dnra + a*ROM.SO4 + a/2*ROM.CH4 + (Rch4o2 + Rch4so4)*por + Rch4no3*por
R.TA <- (b-c)*ROM.O2 + (4*a/5 +b-c)*ROM.NO3dnf +(a/2+b-c)*ROM.NO3dnra +(a+b-c)*ROM.SO4 + (b-c)*ROM.CH4 +(-2*Rnh4o2 -2*Rtso2 +2*Rch4so4)*por + 1.6*Rch4no3*por
R.CH4 <- a/2*ROM.CH4 + (-Rch4o2 -Rch4so4)*por - Rch4no3*por

Rgas <- 0.*Rno3ts
if(iseepage == 0) {
  k13 <- 0
} else {
  k13 <- 1.4/1e9 # s−1
  Ceq <- CH4.seep
  Rgas <- k13 * pmax(Ceq - CH4,0)*por
}

# bioirrigation
RirrO2 <- alpha*por*(O2.ow - pmax(O2,0))
RirrSO4 <- alpha*por*(SO4.ow - pmax(SO4,0))
RirrNO3 <- alpha*por*(NO3.ow - pmax(NO3,0))
RirrNH4 <- alpha*por*(NH4.ow - pmax(NH4,0))
RirrTS <- alpha*por*(TS.ow - pmax(TS,0))
```

```
    RirrDIC <- alpha*por*(DIC.ow - pmax(DIC,0))
    RirrCH4 <- alpha*por*(CH4.ow - pmax(CH4,0))
    RirrTA <- alpha*por*(TA.ow - pmax(TA,0))

    # adding bioirrigation to the solutes
    R.O2 <- R.O2 + RirrO2
    R.SO4 <- R.SO4 + RirrSO4
    R.NO3 <- R.NO3 + RirrNO3
    R.NH4 <- R.NH4 + RirrNH4
    R.TS <- R.TS + RirrTS
    R.DIC <- R.DIC + RirrDIC
    R.CH4 <- R.CH4 + RirrCH4 + Rgas
    R.TA <- R.TA + RirrTA

    # combine reactions and transport
    dO2dt <- tranO2$dC + R.O2
    dSO4dt <- tranSO4$dC + R.SO4
    dNO3dt <- tranNO3$dC + R.NO3
    dNH4dt <- tranNH4$dC + R.NH4
    dTSdt <- tranTS$dC + R.TS
    dDICdt <- tranDIC$dC + R.DIC
    dTAdt <- tranTA$dC + R.TA
    dCH4dt <- tranCH4$dC + R.CH4

     print(paste("yr:", t/365/24/3600,iseepage))

    # Assemble the total rate of change, and return fluxes and reaction rates
    return(list(c(dO2dt = dO2dt, dSO4dt = dSO4dt, dNO3dt = dNO3dt, dNH4dt = dNH4dt,
                  dTSdt = dTSdt, dDICdt = dDICdt, dTAdt = dTAdt, dCH4dt = dCH4dt),
                ROM.O2 = ROM.O2, ROM.NO3dnf = ROM.NO3dnf, ROM.NO3dnra =
ROM.NO3dnra,
                ROM.SO4 = ROM.SO4, ROM.CH4 = ROM.CH4,
                Rtso2 = Rtso2, Rnh4o2 = Rnh4o2,
                Rch4o2 = Rch4o2, Rch4so4 = Rch4so4, Rno3ts = Rno3ts,
                Rgas = Rgas,
                RirrO2 = RirrO2, RirrSO4 = RirrSO4, RirrNO3 = RirrNO3, RirrNH4 = RirrNH4,
                RirrTS = RirrTS, RirrDIC = RirrDIC, RirrTA = RirrTA, RirrCH4 = RirrCH4,
Rch4no3=Rch4no3))
  })}


####################################                    SIMULATION
########################################
# Initialize state variables:
```

```
O2.in <- rep(0, length.out = N)
SO4.in <- rep(SO4.ow, length.out = N)
NO3.in <- rep(0, length.out = N)
NH4.in <- rep(NH4.ow, length.out = N)
TS.in <- rep(0, length.out = N)
DIC.in <- rep(AE.ow$SumCO2 * rho_sw, length.out = N)
TA.in <- rep(AE.ow$TA * rho_sw, length.out = N)
CH4.in <- rep(0, length.out = N)
state <- c(O2.in, SO4.in, NO3.in, NH4.in, TS.in, DIC.in, TA.in, CH4.in)
ic0 <- c(state) # save the initial conditions
chem.spec <- c("O2", "SO4", "NO3", "NH4", "TS", "DIC", "TA", "CH4")


# ======================== first run to steady state without seepage
========================#
print("start stst")
u <- ucompaction
iseepage <- 0

# bioirrigation ---------------------------------------------
alpha0 <- 200/secperyr # 140/yr [1/s]
alphaml_depth = 0.03 # mixed layer depth [m]
alpha_efold <- 0.01 # decay of irrigation coefficient with depth [m]
alpha1 <- setup.prop.1D(func = p.exp,
                        grid = grid,
                        y.0 = alpha0, y.inf = 0, x.L = alphaml_depth, x.att = alpha_efold)
#y.0 = 10 (Owings et al., 2021)
alpha <- alpha1$mid
plot_profile(x = alpha, main = "malpha", ylab = "depth (m)")

# bioturbation ---------------------------------------------
Db0 = 0.0024/secperyr # [m2/s]
Dbml_depth = 0.03 # mixed layer depth [m]
Db1 <- setup.prop.1D(func = p.exp,
                     grid = grid,
                     y.0 = Db0, y.inf = 0, x.L = Dbml_depth, x.att = 0.01) #y.0 = 10 (Owings
et al., 2021)
Db <- Db1$mid
plot_profile(x = Db, main = "mDb", ylab = "depth (m)")

# make RC(z) follow Db profile
RC1 <- setup.prop.1D(func = p.exp,
                     grid = grid,
                     y.0 = RC0, y.inf = 0, x.L = Dbml_depth, x.att = betaOM) #y.0 = 10
```

(Owings et al., 2021)

```
RC <- RC1$mid
plot_profile(x = RC, main = "RCd", ylab = "depth (m)")


parms <- c(iseepage = iseepage,
           alpha = alpha,
           Db = Db,
           u = u,
           RC = RC)

std <- steady.1D(y = state, func = ED.model, parms = parms,
                 names = chem.spec, nspec = length(chem.spec), method = "stode")
ic1 <- std   # steady state results
# use results as new initial conditions
O2s.out   <- ic1$y[, "O2"]
SO4s.out <- ic1$y[, "SO4"]
NO3s.out <- ic1$y[, "NO3"]
NH4s.out <- ic1$y[, "NH4"]
TSs.out   <- ic1$y[, "TS"]
DICs.out <- ic1$y[, "DIC"]
TAs.out   <- ic1$y[, "TA"]
CH4s.out <- ic1$y[, "CH4"]
ROM.O2s.out   <- ic1$ROM.O2
ROM.NO3dnfs.out <- ic1$ROM.NO3dnf
ROM.NO3dnras.out <- ic1$ROM.NO3dnra
ROM.SO4s.out <- ic1$ROM.SO4
ROM.CH4s.out <- ic1$ROM.CH4
Rtso2s.out <- ic1$Rtso2
Rnh4o2s.out   <- ic1$Rnh4o2
Rch4o2s.out <- ic1$Rch4o2
Rch4so4s.out   <- ic1$Rch4so4
Rno3tss.out   <- ic1$Rno3ts
Rgas.out   <- ic1$Rgas
RirrO2.out   <- ic1$RirrO2
RirrSO4.out   <- ic1$RirrSO4
RirrNO3.out   <- ic1$RirrNO3
RirrNH4.out   <- ic1$RirrNH4
RirrTS.out   <- ic1$RirrTS
RirrDIC.out   <- ic1$RirrDIC
RirrTA.out   <- ic1$RirrTA
RirrCH4.out   <- ic1$RirrCH4
Rch4no3.out <- ic1$Rch4no3
```

```
state <- c(pmax(O2s.out,0), pmax(SO4s.out,0), pmax(NO3s.out,0), pmax(NH4s.out,0), pmax(TSs.out,0), pmax(DICs.out,0), pmax(TAs.out,0), pmax(CH4s.out,0))
ic1stst1 <- ic1

# ============================ # transient to stst (steady state Non Seep) # ============================ #
solv_tol <- 1e-7 #1e-3
tstart <- 0 # start time
tend <- 10*secperyr # end time (sec). 5 years are to just reach the surface, then another 5 yrs
dtout <- 1*secperyr # timestep for output
times <- seq(tstart, tend, by=dtout)

strans0 <- ode.1D(y = state, func = ED.model, parms = parms, rtol = solv_tol, atol=solv_tol,
                  names = chem.spec, nspec = length(chem.spec), times = times, method="lsode")
ic1t <- strans0[length(times),]   # final time results
# C(x,t)
outtime <- strans0[,1]
outO2 <- strans0[,2:(N+1)]
outSO4 <- strans0[,(N+1+1):(1+2*N)]
outNO3   <- strans0[,(2+2*N):(1+3*N)]
outNH4   <- strans0[,(2+3*N):(1+4*N)]
outTS   <- strans0[,(2+4*N):(1+5*N)]
outDIC <- strans0[,(2+5*N):(1+6*N)]
outTA   <- strans0[,(2+6*N):(1+7*N)]
outCH4   <- strans0[,(2+7*N):(1+8*N)]
outROM.O2 <- strans0[,(2+8*N):(1+9*N)]
outROM.NO3dnf <- strans0[,(2+9*N):(1+10*N)]
outROM.NO3dnra <- strans0[,(2+10*N):(1+11*N)]
outROM.SO4 <- strans0[,(2+11*N):(1+12*N)]
outROM.CH4 <- strans0[,(2+12*N):(1+13*N)]
outRtso2 <- strans0[,(2+13*N):(1+14*N)]
outRnh4o2 <- strans0[,(2+14*N):(1+15*N)]
outRch4o2 <- strans0[,(2+15*N):(1+16*N)]
outRch4so4 <- strans0[,(2+16*N):(1+17*N)]
outRno3ts <- strans0[,(2+17*N):(1+18*N)]
outRgas <- strans0[,(2+18*N):(1+19*N)]
outRirrO2 <- strans0[,(2+19*N):(1+20*N)]
outRirrSO4 <- strans0[,(2+20*N):(1+21*N)]
outRirrNO3 <- strans0[,(2+21*N):(1+22*N)]
outRirrNH4 <- strans0[,(2+22*N):(1+23*N)]
outRirrTS <- strans0[,(2+23*N):(1+24*N)]
outRirrDIC <- strans0[,(2+24*N):(1+25*N)]
outRirrTA <- strans0[,(2+25*N):(1+26*N)]
```

```
outRirrCH4 <- strans0[,(2+26*N):(1+27*N)]
outRch4no3 <- strans0[,(2+27*N):(1+28*N)]
# C(x,final t)
O2s.out    <- ic1t[2:(N+1)]
SO4s.out <- ic1t[(N+1+1):(1+2*N)]
NO3s.out    <- ic1t[(2+2*N):(1+3*N)]
NH4s.out <- ic1t[(2+3*N):(1+4*N)]
TSs.out    <- ic1t[(2+4*N):(1+5*N)]
DICs.out    <- ic1t[(2+5*N):(1+6*N)]
TAs.out    <- ic1t[(2+6*N):(1+7*N)]
CH4s.out    <- ic1t[(2+7*N):(1+8*N)]
ROM.O2s.out    <- ic1t[(2+8*N):(1+9*N)]
ROM.NO3dnfs.out    <- ic1t[(2+9*N):(1+10*N)]
ROM.NO3dnras.out    <- ic1t[(2+10*N):(1+11*N)]
ROM.SO4s.out    <- ic1t[(2+11*N):(1+12*N)]
ROM.CH4s.out    <- ic1t[(2+12*N):(1+13*N)]
Rtso2s.out    <- ic1t[(2+13*N):(1+14*N)]
Rnh4o2s.out    <- ic1t[(2+14*N):(1+15*N)]
Rch4o2s.out    <- ic1t[(2+15*N):(1+16*N)]
Rch4so4s.out    <- ic1t[(2+16*N):(1+17*N)]
Rno3tss.out    <- ic1t[(2+17*N):(1+18*N)]
Rgas.out    <- ic1t[(2+18*N):(1+19*N)]
RirrO2.out    <- ic1t[(2+19*N):(1+20*N)]
RirrSO4.out    <- ic1t[(2+20*N):(1+21*N)]
RirrNO3.out    <- ic1t[(2+21*N):(1+22*N)]
RirrNH4.out    <- ic1t[(2+22*N):(1+23*N)]
RirrTS.out    <- ic1t[(2+23*N):(1+24*N)]
RirrDIC.out    <- ic1t[(2+24*N):(1+25*N)]
RirrTA.out    <- ic1t[(2+25*N):(1+26*N)]
RirrCH4.out    <- ic1t[(2+26*N):(1+27*N)]
Rch4no3.out    <- ic1t[(2+27*N):(1+28*N)]

paste("initial calculation to stst ended: ", Sys.time())
state <- c(O2s.out, SO4s.out, NO3s.out, NH4s.out, TSs.out, DICs.out, TAs.out, CH4s.out)
# state <- c(pmax(O2s.out,0), pmax(SO4s.out,0), pmax(NO3s.out,0), pmax(NH4s.out,0), pmax(TSs.out,0), pmax(DICs.out,0), pmax(TAs.out,0), pmax(CH4s.out,0))

## Integrate the rates (unit: mmol m-2 d-1) Use the final rates to integrate
# kk = list(outO2, outSO4, outNO3, outNH4, outTS, outDIC, outTA, outCH4)
ROM.O2s.out    <-         ic1t[(2+8*N):(1+9*N)]
ROM.NO3dnfs.out    <- ic1t[(2+9*N):(1+10*N)]
ROM.NO3dnras.out <- ic1t[(2+10*N):(1+11*N)]
ROM.SO4s.out    <-       ic1t[(2+11*N):(1+12*N)]
ROM.CH4s.out    <-       ic1t[(2+12*N):(1+13*N)]
```

```
Rtso2s.out    <-          ic1t[(2+13*N):(1+14*N)]
Rnh4o2s.out    <-         ic1t[(2+14*N):(1+15*N)]
Rch4o2s.out    <-         ic1t[(2+15*N):(1+16*N)]
Rch4so4s.out    <-       ic1t[(2+16*N):(1+17*N)]
Rno3tss.out    <-         ic1t[(2+17*N):(1+18*N)]
Rch4no3.out    <-         ic1t[(2+27*N):(1+28*N)]

library(dplyr)
secperday = 3600*24

# 0-100cm
Integrating_rate <- function(r.x) {
   int_rate = data.frame(as.matrix(r.x))
   flux    =    sum(int_rate[1:400,])*(L/N)*secperday*1000
   return(flux)}

# Make the data.frame
df_flux = data.frame(Integrating_rate(ROM.O2s.out),
                     Integrating_rate(ROM.NO3dnfs.out),
                     Integrating_rate(ROM.NO3dnras.out),
                     Integrating_rate(ROM.SO4s.out),
                     Integrating_rate(ROM.CH4s.out),
                     Integrating_rate(Rch4o2s.out),
                     Integrating_rate(Rch4so4s.out),
                     Integrating_rate(Rnh4o2s.out),
                     Integrating_rate(Rtso2s.out),
                     Integrating_rate(Rno3tss.out),
                     Integrating_rate(Rch4no3.out))
df_flux2 = data.frame(t(df_flux))
df_flux2$cat          =          c("OM.O2_flux",          "OM.DNF_flux",
"OM.DNRA_flux","OM.SO4_flux","OM.CH4_flux",
                "CH4_O2_flux","CH4_SO4_flux",
                "NH4_O2_flux","TS_O2_flux","TS_NO3_flux","CH4_NO3_flux")
df_flux2$t.df_flux.
df_flux2
# Save results
df_flux2_non_seep_0_100cm = df_flux2
# Set the levels
df_flux2 = arrange(df_flux2, desc(t.df_flux.))
level <- df_flux2[,2]
df_flux2$cat <- factor(df_flux2$cat,level = rev(level))
# Plot bar
p <- ggplot(df_flux2)+
   geom_bar(aes(x = cat, y = t.df_flux., fill = t.df_flux.),
```

```
                 stat = 'identity', position = 'stack', show.legend=TRUE)+
  ylab("Flux mmol m-2 d-1")+
  xlab("")+
  coord_flip()+
  scale_y_log10()
p

# 0-50cm
Integrating_rate <- function(r.x) {
  int_rate = data.frame(as.matrix(r.x))
  flux   =   sum(int_rate[1:200,])*(L/N)*secperday*1000
  return(flux)}

# Make the data.frame
df_flux = data.frame(Integrating_rate(ROM.O2s.out),
                                    Integrating_rate(ROM.NO3dnfs.out),
                                    Integrating_rate(ROM.NO3dnras.out),
                                    Integrating_rate(ROM.SO4s.out),
                                    Integrating_rate(ROM.CH4s.out),
                                    Integrating_rate(Rch4o2s.out),
                                    Integrating_rate(Rch4so4s.out),
                                    Integrating_rate(Rnh4o2s.out),
                                    Integrating_rate(Rtso2s.out),
                                    Integrating_rate(Rno3tss.out),
                                    Integrating_rate(Rch4no3.out))
df_flux2 = data.frame(t(df_flux))
df_flux2$cat                    =                    c("OM.O2_flux",                    "OM.DNF_flux",
"OM.DNRA_flux","OM.SO4_flux","OM.CH4_flux",
                              "CH4_O2_flux","CH4_SO4_flux",
                              "NH4_O2_flux","TS_O2_flux","TS_NO3_flux","CH4_NO3_flux")
df_flux2$t.df_flux.
df_flux2
# Save results
df_flux2_non_seep_0_50cm = df_flux2
# Set the levels
df_flux2 = arrange(df_flux2, desc(t.df_flux.))
level <- df_flux2[,2]
df_flux2$cat <- factor(df_flux2$cat,level = rev(level))
# Plot bar
p <- ggplot(df_flux2)+
  geom_bar(aes(x = cat, y = t.df_flux., fill = t.df_flux.),
                 stat = 'identity', position = 'stack', show.legend=TRUE)+
  ylab("Flux mmol m-2 d-1")+
  xlab("")+
```

```
  coord_flip()+
  scale_y_log10()
p

# 50-100cm
Integrating_rate <- function(r.x) {
  int_rate = data.frame(as.matrix(r.x))
  flux  =  sum(int_rate[201:400,])*(L/N)*secperday*1000
  return(flux)}

# Make the data.frame
df_flux = data.frame(Integrating_rate(ROM.O2s.out),
                     Integrating_rate(ROM.NO3dnfs.out),
                     Integrating_rate(ROM.NO3dnras.out),
                     Integrating_rate(ROM.SO4s.out),
                     Integrating_rate(ROM.CH4s.out),
                     Integrating_rate(Rch4o2s.out),
                     Integrating_rate(Rch4so4s.out),
                     Integrating_rate(Rnh4o2s.out),
                     Integrating_rate(Rtso2s.out),
                     Integrating_rate(Rno3tss.out),
                     Integrating_rate(Rch4no3.out))
df_flux2 = data.frame(t(df_flux))
df_flux2$cat = c("OM.O2_flux", "OM.DNF_flux",
"OM.DNRA_flux","OM.SO4_flux","OM.CH4_flux",
                 "CH4_O2_flux","CH4_SO4_flux",
                 "NH4_O2_flux","TS_O2_flux","TS_NO3_flux","CH4_NO3_flux")
df_flux2$t.df_flux.
df_flux2
# Save results
df_flux2_non_seep_50_100cm = df_flux2
# Set the levels
df_flux2 = arrange(df_flux2, desc(t.df_flux.))
level <- df_flux2[,2]
df_flux2$cat <- factor(df_flux2$cat,level = rev(level))
# Plot bar
p <- ggplot(df_flux2)+
  geom_bar(aes(x = cat, y = t.df_flux., fill = t.df_flux.),
           stat = 'identity', position = 'stack', show.legend=TRUE)+
  ylab("Flux mmol m-2 d-1")+
  xlab("")+
  coord_flip()+
  scale_y_log10()
p
```

```
# ========================== # then transient with seepage (Newborn Seep) #
=========================== #
paste("transient calculation started: ", Sys.time())
u <- ucompaction + useepage
iseepage <- 1 # to pick up the right lower boundary conditions
solv_tol <- 1e-7 #1e-3 #1e-4 /-7

# transient simulations
tstart <- 0 # start time
tend <- (5+5)*secperyr # end time (sec). 5 years are to just reach the surface, then another 5
yrs
dtout <- 1*secperyr # timestep for output
times <- seq(tstart, tend, by=dtout)

# bioirrigation --------------------------------------------
alpha0 <- 200/secperyr # 140/yr [1/s]
alphaml_depth = 0.3# mixed layer depth [m]
alpha_efold <- 0.1# 0.005 # decay of irrigation coefficient with depth [m]
alpha1 <- setup.prop.1D(func = p.exp,
                        grid = grid,
                        y.0 = alpha0, y.inf = 0, x.L = alphaml_depth, x.att = alpha_efold)
#y.0 = 10 (Owings et al., 2021)
alpha <- alpha1$mid
plot_profile(x = alpha, main = "alpha", ylab = "depth (m)")


# bioturbation --------------------------------------------
Db0 = 0.0024/secperyr # [m2/s]
Dbml_depth = 0.1 # mixed layer depth [m]
Db1 <- setup.prop.1D(func = p.exp,
                     grid = grid,
                     y.0 = Db0, y.inf = 0, x.L = Dbml_depth, x.att = 0.1) #y.0 = 10 (Owings
et al., 2021)
plot_profile(x = Db1$mid, main = "Db1", ylab = "depth (m)")
Db <- Db1$mid
plot_profile(x = Db, main = "Db", ylab = "depth (m)")

RC1 <- setup.prop.1D(func = p.exp,
                     grid = grid,
                     y.0 = RC0, y.inf = 0, x.L = 0.3, x.att = betaOM) #y.0 = 10 (Owings et
al., 2021)
RC <- RC1$mid
```

```
plot_profile(x = RC, main = "rddb", ylab = "depth (m)")

parms <- c(iseepage = iseepage,
           alpha = alpha,
           Db = Db,
           u = u,
           RC = RC)
trans0 <- ode.1D(y = state, func = ED.model, parms = parms, rtol = solv_tol, atol=solv_tol,
                 names = chem.spec, nspec = length(chem.spec), times = times,
method="lsode", maxsteps = 50000)
# radau
ic2 <- trans0[length(times),]   # final time results

# C(x,t)
outtime <- trans0[,1]
outO2 <- trans0[,2:(N+1)]
outSO4 <- trans0[,(N+1+1):(1+2*N)]
outNO3   <- trans0[,(2+2*N):(1+3*N)]
outNH4   <- trans0[,(2+3*N):(1+4*N)]
outTS   <- trans0[,(2+4*N):(1+5*N)]
outDIC <- trans0[,(2+5*N):(1+6*N)]
outTA   <- trans0[,(2+6*N):(1+7*N)]
outCH4   <- trans0[,(2+7*N):(1+8*N)]
outROM.O2 <- trans0[,(2+8*N):(1+9*N)]
outROM.NO3dnf <- trans0[,(2+9*N):(1+10*N)]
outROM.NO3dnra <- trans0[,(2+10*N):(1+11*N)]
outROM.SO4 <- trans0[,(2+11*N):(1+12*N)]
outROM.CH4 <- trans0[,(2+12*N):(1+13*N)]
outRtso2 <- trans0[,(2+13*N):(1+14*N)]
outRnh4o2 <- trans0[,(2+14*N):(1+15*N)]
outRch4o2 <- trans0[,(2+15*N):(1+16*N)]
outRch4so4 <- trans0[,(2+16*N):(1+17*N)]
outRno3ts <- trans0[,(2+17*N):(1+18*N)]

outRgas <- trans0[,(2+18*N):(1+19*N)]
outRirrO2 <- trans0[,(2+19*N):(1+20*N)]
outRirrSO4 <- trans0[,(2+20*N):(1+21*N)]
outRirrNO3 <- trans0[,(2+21*N):(1+22*N)]
outRirrNH4 <- trans0[,(2+22*N):(1+23*N)]
outRirrTS <- trans0[,(2+23*N):(1+24*N)]
outRirrDIC <- trans0[,(2+24*N):(1+25*N)]
outRirrTA <- trans0[,(2+25*N):(1+26*N)]
outRirrCH4 <- trans0[,(2+26*N):(1+27*N)]
outRch4no3 <- trans0[,(2+27*N):(1+28*N)]
```

```
paste("transient calculation ended: ", Sys.time())

# C(x,final t)
O2.out    <- ic2[2:(N+1)]
SO4.out <- ic2[(N+1+1):(1+2*N)]
NO3.out    <- ic2[(2+2*N):(1+3*N)]
NH4.out <- ic2[(2+3*N):(1+4*N)]
TS.out    <- ic2[(2+4*N):(1+5*N)]
DIC.out    <- ic2[(2+5*N):(1+6*N)]
TA.out    <- ic2[(2+6*N):(1+7*N)]
CH4.out    <- ic2[(2+7*N):(1+8*N)]
ROM.O2.out    <- ic2[(2+8*N):(1+9*N)]
ROM.NO3dnf.out    <- ic2[(2+9*N):(1+10*N)]
ROM.NO3dnra.out    <- ic2[(2+10*N):(1+11*N)]
ROM.SO4.out    <- ic2[(2+11*N):(1+12*N)]
ROM.CH4.out    <- ic2[(2+12*N):(1+13*N)]
Rtso2.out    <- ic2[(2+13*N):(1+14*N)]
Rnh4o2.out    <- ic2[(2+14*N):(1+15*N)]
Rch4o2.out    <- ic2[(2+15*N):(1+16*N)]
Rch4so4.out    <- ic2[(2+16*N):(1+17*N)]
Rno3ts.out    <- ic2[(2+17*N):(1+18*N)]

Rgas.out    <- ic2[(2+18*N):(1+19*N)]
RirrO2.out    <- ic2[(2+19*N):(1+20*N)]
RirrSO4.out    <- ic2[(2+20*N):(1+21*N)]
RirrNO3.out    <- ic2[(2+21*N):(1+22*N)]
RirrNH4.out    <- ic2[(2+22*N):(1+23*N)]
RirrTS.out    <- ic2[(2+23*N):(1+24*N)]
RirrDIC.out    <- ic2[(2+24*N):(1+25*N)]
RirrTA.out    <- ic2[(2+25*N):(1+26*N)]
RirrCH4.out    <- ic2[(2+26*N):(1+27*N)]
Rch4no3.out    <- ic2[(2+27*N):(1+28*N)]

## Integrate the rates (unit: mmol m-2 d-1)
# kk = list(outO2, outSO4, outNO3, outNH4, outTS, outDIC, outTA, outCH4)
outROM.O2           <- trans0[,(2+8*N):(1+9*N)]
outROM.NO3dnf    <- trans0[,(2+9*N):(1+10*N)]
outROM.NO3dnra <- trans0[,(2+10*N):(1+11*N)]
outROM.SO4         <- trans0[,(2+11*N):(1+12*N)]
outROM.CH4         <- trans0[,(2+12*N):(1+13*N)]
outRtso2             <- trans0[,(2+13*N):(1+14*N)]
outRnh4o2.         <- trans0[,(2+14*N):(1+15*N)]
outRch4o2.         <- trans0[,(2+15*N):(1+16*N)]
```

```
outRch4so4.       <- trans0[,(2+16*N):(1+17*N)]
outRno3ts         <- trans0[,(2+17*N):(1+18*N)]
outRch4no3        <- trans0[,(2+27*N):(1+28*N)]

library(dplyr)
secperday = 3600*24

## --------------------------------- Stage I ---------------------------------##
# 0-100cm
Integrating_rate <- function(r.x) {
   int_rate = data.frame(as.matrix(t(r.x)))
   flux    =    (sum(int_rate$X1[1:400])*(L/N)*secperday*1000+
                 sum(int_rate$X2[1:400])*(L/N)*secperday*1000+
                 sum(int_rate$X3[1:400])*(L/N)*secperday*1000)/3
   return(flux)}

# Make the data.frame
df_flux = data.frame(Integrating_rate(outROM.O2),
                     Integrating_rate(outROM.NO3dnf),
                     Integrating_rate(outROM.NO3dnra),
                     Integrating_rate(outROM.SO4),
                     Integrating_rate(outROM.CH4),
                     Integrating_rate(outRch4o2),
                     Integrating_rate(outRch4so4),
                     Integrating_rate(outRnh4o2),
                     Integrating_rate(outRtso2),
                     Integrating_rate(outRno3ts),
                     Integrating_rate(outRch4no3))
df_flux2 = data.frame(t(df_flux))
df_flux2$cat            =            c("OM.O2_flux",            "OM.DNF_flux",
"OM.DNRA_flux","OM.SO4_flux","OM.CH4_flux",
               "CH4_O2_flux","CH4_SO4_flux",
               "NH4_O2_flux","TS_O2_flux","TS_NO3_flux","CH4_NO3_flux")
df_flux2$t.df_flux.
df_flux2
# Save results
df_flux2_stageI_0_100cm = df_flux2
# Set the levels
df_flux2 = arrange(df_flux2, desc(t.df_flux.))
level <- df_flux2[,2]
df_flux2$cat <- factor(df_flux2$cat,level = rev(level))
# Plot bar
p <- ggplot(df_flux2)+
   geom_bar(aes(x = cat, y = t.df_flux., fill = t.df_flux.),
```

```
                stat = 'identity', position = 'stack', show.legend=TRUE)+
   ylab("Flux mmol m-2 d-1")+
   xlab("")+
   coord_flip()+
   scale_y_log10()
p

# 0-50cm
Integrating_rate <- function(r.x) {
   int_rate = data.frame(as.matrix(t(r.x)))
   flux    =    (sum(int_rate$X1[1:200])*(L/N)*secperday*1000+
                    sum(int_rate$X2[1:200])*(L/N)*secperday*1000+
                    sum(int_rate$X3[1:200])*(L/N)*secperday*1000)/3
   return(flux)}

# Make the data.frame
df_flux = data.frame(Integrating_rate(outROM.O2),
                              Integrating_rate(outROM.NO3dnf),
                              Integrating_rate(outROM.NO3dnra),
                              Integrating_rate(outROM.SO4),
                              Integrating_rate(outROM.CH4),
                              Integrating_rate(outRch4o2),
                              Integrating_rate(outRch4so4),
                              Integrating_rate(outRnh4o2),
                              Integrating_rate(outRtso2),
                              Integrating_rate(outRno3ts),
                              Integrating_rate(outRch4no3))
df_flux2 = data.frame(t(df_flux))
df_flux2$cat                    =                    c("OM.O2_flux",                    "OM.DNF_flux",
"OM.DNRA_flux","OM.SO4_flux","OM.CH4_flux",
                         "CH4_O2_flux","CH4_SO4_flux",
                         "NH4_O2_flux","TS_O2_flux","TS_NO3_flux","CH4_NO3_flux")
df_flux2$t.df_flux.
df_flux2
# Save results
df_flux2_stageI_0_50cm = df_flux2
# Set the levels
df_flux2 = arrange(df_flux2, desc(t.df_flux.))
level <- df_flux2[,2]
df_flux2$cat <- factor(df_flux2$cat,level = rev(level))
# Plot bar
p <- ggplot(df_flux2)+
   geom_bar(aes(x = cat, y = t.df_flux., fill = t.df_flux.),
                stat = 'identity', position = 'stack', show.legend=TRUE)+
```

```
  ylab("Flux mmol m-2 d-1")+
  xlab("")+
  coord_flip()+
  scale_y_log10()
p

# 50-100cm
Integrating_rate <- function(r.x) {
  int_rate = data.frame(as.matrix(t(r.x)))
  flux   =   (sum(int_rate$X1[201:400])*(L/N)*secperday*1000+
                   sum(int_rate$X2[201:400])*(L/N)*secperday*1000+
                   sum(int_rate$X3[201:400])*(L/N)*secperday*1000)/3
  return(flux)}

# Make the data.frame
df_flux = data.frame(Integrating_rate(outROM.O2),
                           Integrating_rate(outROM.NO3dnf),
                           Integrating_rate(outROM.NO3dnra),
                           Integrating_rate(outROM.SO4),
                           Integrating_rate(outROM.CH4),
                           Integrating_rate(outRch4o2),
                           Integrating_rate(outRch4so4),
                           Integrating_rate(outRnh4o2),
                           Integrating_rate(outRtso2),
                           Integrating_rate(outRno3ts),
                           Integrating_rate(outRch4no3))
df_flux2 = data.frame(t(df_flux))
df_flux2$cat             =             c("OM.O2_flux",             "OM.DNF_flux",
"OM.DNRA_flux","OM.SO4_flux","OM.CH4_flux",
                  "CH4_O2_flux","CH4_SO4_flux",
                  "NH4_O2_flux","TS_O2_flux","TS_NO3_flux","CH4_NO3_flux")
df_flux2$t.df_flux.
df_flux2
# Save results
df_flux2_stageI_50_100cm = df_flux2
# Set the levels
df_flux2 = arrange(df_flux2, desc(t.df_flux.))
level <- df_flux2[,2]
df_flux2$cat <- factor(df_flux2$cat,level = rev(level))
# Plot bar
p <- ggplot(df_flux2)+
  geom_bar(aes(x = cat, y = t.df_flux., fill = t.df_flux.),
             stat = 'identity', position = 'stack', show.legend=TRUE)+
  ylab("Flux mmol m-2 d-1")+
```

```
  xlab("")+
  coord_flip()+
  scale_y_log10()
p

## ------------------------------- Stage II-------------------------------##
# 0-100cm
Integrating_rate <- function(r.x) {
  int_rate = data.frame(as.matrix(t(r.x)))
  flux   =   (
    sum(int_rate$X4[1:400])*(L/N)*secperday*1000+
      sum(int_rate$X5[1:400])*(L/N)*secperday*1000+
      sum(int_rate$X6[1:400])*(L/N)*secperday*1000)/3
  return(flux)}

# Make the data.frame
df_flux = data.frame(Integrating_rate(outROM.O2),
                     Integrating_rate(outROM.NO3dnf),
                     Integrating_rate(outROM.NO3dnra),
                     Integrating_rate(outROM.SO4),
                     Integrating_rate(outROM.CH4),
                     Integrating_rate(outRch4o2),
                     Integrating_rate(outRch4so4),
                     Integrating_rate(outRnh4o2),
                     Integrating_rate(outRtso2),
                     Integrating_rate(outRno3ts),
                     Integrating_rate(outRch4no3))
df_flux2 = data.frame(t(df_flux))
df_flux2$cat          =          c("OM.O2_flux",          "OM.DNF_flux",
"OM.DNRA_flux","OM.SO4_flux","OM.CH4_flux",
                 "CH4_O2_flux","CH4_SO4_flux",
                 "NH4_O2_flux","TS_O2_flux","TS_NO3_flux","CH4_NO3_flux")
df_flux2$t.df_flux.
df_flux2
# Save results
df_flux2_stageII_0_100cm = df_flux2
# Set the levels
df_flux2 = arrange(df_flux2, desc(t.df_flux.))
level <- df_flux2[,2]
df_flux2$cat <- factor(df_flux2$cat,level = rev(level))
# Plot bar
p <- ggplot(df_flux2)+
  geom_bar(aes(x = cat, y = t.df_flux., fill = t.df_flux.),
           stat = 'identity', position = 'stack', show.legend=TRUE)+
```

```
  ylab("Flux mmol m-2 d-1")+
  xlab("")+
  coord_flip()+
  scale_y_log10()
p

# 0-50cm
Integrating_rate <- function(r.x) {
  int_rate = data.frame(as.matrix(t(r.x)))
  flux   =   (
    sum(int_rate$X4[1:200])*(L/N)*secperday*1000+
      sum(int_rate$X5[1:200])*(L/N)*secperday*1000+
      sum(int_rate$X6[1:200])*(L/N)*secperday*1000)/3
  return(flux)}

# Make the data.frame
df_flux = data.frame(Integrating_rate(outROM.O2),
                     Integrating_rate(outROM.NO3dnf),
                     Integrating_rate(outROM.NO3dnra),
                     Integrating_rate(outROM.SO4),
                     Integrating_rate(outROM.CH4),
                     Integrating_rate(outRch4o2),
                     Integrating_rate(outRch4so4),
                     Integrating_rate(outRnh4o2),
                     Integrating_rate(outRtso2),
                     Integrating_rate(outRno3ts),
                     Integrating_rate(outRch4no3))
df_flux2 = data.frame(t(df_flux))
df_flux2$cat          =          c("OM.O2_flux",          "OM.DNF_flux",
"OM.DNRA_flux","OM.SO4_flux","OM.CH4_flux",
                  "CH4_O2_flux","CH4_SO4_flux",
                  "NH4_O2_flux","TS_O2_flux","TS_NO3_flux","CH4_NO3_flux")
df_flux2$t.df_flux.
df_flux2
# Save results
df_flux2_stageII_0_50cm = df_flux2
# Set the levels
df_flux2 = arrange(df_flux2, desc(t.df_flux.))
level <- df_flux2[,2]
df_flux2$cat <- factor(df_flux2$cat,level = rev(level))
# Plot bar
p <- ggplot(df_flux2)+
  geom_bar(aes(x = cat, y = t.df_flux., fill = t.df_flux.),
           stat = 'identity', position = 'stack', show.legend=TRUE)+
```

```
  ylab("Flux mmol m-2 d-1")+
  xlab("")+
  coord_flip()+
  scale_y_log10()
p

# 50-100cm
Integrating_rate <- function(r.x) {
  int_rate = data.frame(as.matrix(t(r.x)))
  flux   =   (
    sum(int_rate$X4[201:400])*(L/N)*secperday*1000+
      sum(int_rate$X5[201:400])*(L/N)*secperday*1000+
      sum(int_rate$X6[201:400])*(L/N)*secperday*1000)/3
  return(flux)}

# Make the data.frame
df_flux = data.frame(Integrating_rate(outROM.O2),
                     Integrating_rate(outROM.NO3dnf),
                     Integrating_rate(outROM.NO3dnra),
                     Integrating_rate(outROM.SO4),
                     Integrating_rate(outROM.CH4),
                     Integrating_rate(outRch4o2),
                     Integrating_rate(outRch4so4),
                     Integrating_rate(outRnh4o2),
                     Integrating_rate(outRtso2),
                     Integrating_rate(outRno3ts),
                     Integrating_rate(outRch4no3))
df_flux2 = data.frame(t(df_flux))
df_flux2$cat = c("OM.O2_flux", "OM.DNF_flux",
"OM.DNRA_flux","OM.SO4_flux","OM.CH4_flux",
                 "CH4_O2_flux","CH4_SO4_flux",
                 "NH4_O2_flux","TS_O2_flux","TS_NO3_flux","CH4_NO3_flux")
df_flux2$t.df_flux.
df_flux2
# Save results
df_flux2_stageII_50_100cm = df_flux2
# Set the levels
df_flux2 = arrange(df_flux2, desc(t.df_flux.))
level <- df_flux2[,2]
df_flux2$cat <- factor(df_flux2$cat,level = rev(level))
# Plot bar
p <- ggplot(df_flux2)+
  geom_bar(aes(x = cat, y = t.df_flux., fill = t.df_flux.),
           stat = 'identity', position = 'stack', show.legend=TRUE)+
```

```
  ylab("Flux mmol m-2 d-1")+
  xlab("")+
  coord_flip()+
  scale_y_log10()
p


# ============================ # mature seep # ============================ #
u <- ucompaction + (-0.25/secperyr)
iseepage <- 1 # to pick up the right lower boundary conditions
solv_tol <- 1e-7 #1e-4

# transient simulations
tstart <- 0 # start time
tend <- 20*secperyr # end time (sec). 5 years are to just reach the surface, then another 5 yrs
dtout <- 1*secperyr # timestep for output
times2 <- seq(tstart, tend, by=dtout)

# bioirrigation ---------------------------------------------
alpha0 <- 200/secperyr # 140/yr [1/s]
alphaml_depth = 0.03 # mixed layer depth [m]
alpha_efold <- 0.01 # decay of irrigation coefficient with depth [m]
alpha1 <- setup.prop.1D(func = p.exp,
                        grid = grid,
                        y.0 = alpha0, y.inf = 0, x.L = alphaml_depth, x.att = alpha_efold)
#y.0 = 10 (Owings et al., 2021)
alpha <- alpha1$mid
plot_profile(x = alpha, main = "malpha", ylab = "depth (m)")

# bioturbation ---------------------------------------------
Db0 = 0.0024/secperyr # [m2/s]
Dbml_depth = 0.03 # mixed layer depth [m]
Db1 <- setup.prop.1D(func = p.exp,
                     grid = grid,
                     y.0 = Db0, y.inf = 0, x.L = Dbml_depth, x.att = 0.01) #y.0 = 10 (Owings et al., 2021)
Db <- Db1$mid
plot_profile(x = Db, main = "mDb", ylab = "depth (m)")

RC1 <- setup.prop.1D(func = p.exp,
                     grid = grid,
                     y.0 = RC0, y.inf = 0, x.L = 0.03, x.att = betaOM) #y.0 = 10 (Owings et al., 2021)
```

```r
RC <- RC1$mid

parms <- c(iseepage = iseepage,
           alpha = alpha,
           Db = Db,
           u = u,
           RC = RC)
mmstate <- c(O2.out, SO4.out, NO3.out, NH4.out, TS.out, DIC.out, TA.out, CH4.out)
paste("steady state mature seep calculation started: ", Sys.time())
std <- steady.1D(y = mmstate, func = ED.model, parms = parms,
                 names = chem.spec, nspec = length(chem.spec), method = "stode")
ic3 <- std   # steady state results
mmO2.out   <- ic3$y[, "O2"]
mmSO4.out <- ic3$y[, "SO4"]
mmNO3.out <- ic3$y[, "NO3"]
mmNH4.out <- ic3$y[, "NH4"]
mmTS.out   <- ic3$y[, "TS"]
mmDIC.out <- ic3$y[, "DIC"]
mmTA.out   <- ic3$y[, "TA"]
mmCH4.out <- ic3$y[, "CH4"]

mmROM.O2.out   <- ic3$ROM.O2
mmROM.NO3dnf.out <- ic3$ROM.NO3dnf
mmROM.NO3dnra.out <- ic3$ROM.NO3dnra
mmROM.SO4.out <- ic3$ROM.SO4
mmROM.CH4.out <- ic3$ROM.CH4
mmRtso2.out <- ic3$Rtso2
mmRnh4o2.out   <- ic3$Rnh4o2
mmRch4o2.out <- ic3$Rch4o2
mmRch4so4.out   <- ic3$Rch4so4
mmRno3ts.out   <- ic3$Rno3ts

mmRgas.out   <- ic3$Rgas
mmRirrO2.out   <- ic3$RirrO2
mmRirrSO4.out   <- ic3$RirrSO4
mmRirrNO3.out   <- ic3$RirrNO3
mmRirrNH4.out   <- ic3$RirrNH4
mmRirrTS.out   <- ic3$RirrTS
mmRirrDIC.out   <- ic3$RirrDIC
mmRirrTA.out   <- ic3$RirrTA
mmRirrCH4.out   <- ic3$RirrCH4
mmRch4no3.out   <- ic3$Rch4no3

## Integrate the rates (unit: mmol m-2 d-1) Use the final rates to integrate
```

```
library(dplyr)
secperday = 3600*24

# 0-100cm
Integrating_rate <- function(r.x) {
  int_rate = data.frame(as.matrix(r.x))
  flux   =   sum(int_rate[1:400,])*(L/N)*secperday*1000
  return(flux)}

# Make the data.frame
df_flux = data.frame(Integrating_rate(mmROM.O2.out),
                     Integrating_rate(mmROM.NO3dnf.out),
                     Integrating_rate(mmROM.NO3dnra.out),
                     Integrating_rate(mmROM.SO4.out),
                     Integrating_rate(mmROM.CH4.out),
                     Integrating_rate(mmRch4o2.out),
                     Integrating_rate(mmRch4so4.out),
                     Integrating_rate(mmRnh4o2.out),
                     Integrating_rate(mmRtso2.out),
                     Integrating_rate(mmRno3ts.out),
                     Integrating_rate(mmRch4no3.out))
df_flux2 = data.frame(t(df_flux))
df_flux2$cat            =            c("OM.O2_flux",            "OM.DNF_flux",
"OM.DNRA_flux","OM.SO4_flux","OM.CH4_flux",
                  "CH4_O2_flux","CH4_SO4_flux",
                  "NH4_O2_flux","TS_O2_flux","TS_NO3_flux","CH4_NO3_flux")
df_flux2$t.df_flux.
df_flux2
# Save results
df_flux2_mature_seep_0_100cm = df_flux2
# Set the levels
df_flux2 = arrange(df_flux2, desc(t.df_flux.))
level <- df_flux2[,2]
df_flux2$cat <- factor(df_flux2$cat,level = rev(level))
# Plot bar
p <- ggplot(df_flux2)+
  geom_bar(aes(x = cat, y = t.df_flux., fill = t.df_flux.),
           stat = 'identity', position = 'stack', show.legend=TRUE)+
  ylab("Flux mmol m-2 d-1")+
  xlab("")+
  coord_flip()+
  scale_y_log10()
p
```

```
# 0-50cm
Integrating_rate <- function(r.x) {
  int_rate = data.frame(as.matrix(r.x))
  flux  =  sum(int_rate[1:200,])*(L/N)*secperday*1000
  return(flux)}

# Make the data.frame
df_flux = data.frame(Integrating_rate(mmROM.O2.out),
                     Integrating_rate(mmROM.NO3dnf.out),
                     Integrating_rate(mmROM.NO3dnra.out),
                     Integrating_rate(mmROM.SO4.out),
                     Integrating_rate(mmROM.CH4.out),
                     Integrating_rate(mmRch4o2.out),
                     Integrating_rate(mmRch4so4.out),
                     Integrating_rate(mmRnh4o2.out),
                     Integrating_rate(mmRtso2.out),
                     Integrating_rate(mmRno3ts.out),
                     Integrating_rate(mmRch4no3.out))
df_flux2 = data.frame(t(df_flux))
df_flux2$cat = c("OM.O2_flux", "OM.DNF_flux",
"OM.DNRA_flux","OM.SO4_flux","OM.CH4_flux",
                 "CH4_O2_flux","CH4_SO4_flux",
                 "NH4_O2_flux","TS_O2_flux","TS_NO3_flux","CH4_NO3_flux")
df_flux2$t.df_flux.
df_flux2
# Save results
df_flux2_mature_seep_0_50cm = df_flux2
# Set the levels
df_flux2 = arrange(df_flux2, desc(t.df_flux.))
level <- df_flux2[,2]
df_flux2$cat <- factor(df_flux2$cat,level = rev(level))
# Plot bar
p <- ggplot(df_flux2)+
  geom_bar(aes(x = cat, y = t.df_flux., fill = t.df_flux.),
           stat = 'identity', position = 'stack', show.legend=TRUE)+
  ylab("Flux mmol m-2 d-1")+
  xlab("")+
  coord_flip()+
  scale_y_log10()
p

# 50-100cm
Integrating_rate <- function(r.x) {
  int_rate = data.frame(as.matrix(r.x))
```

```
  flux    =   sum(int_rate[201:400,])*(L/N)*secperday*1000
  return(flux)}

# Make the data.frame
df_flux = data.frame(Integrating_rate(mmROM.O2.out),
                     Integrating_rate(mmROM.NO3dnf.out),
                     Integrating_rate(mmROM.NO3dnra.out),
                     Integrating_rate(mmROM.SO4.out),
                     Integrating_rate(mmROM.CH4.out),
                     Integrating_rate(mmRch4o2.out),
                     Integrating_rate(mmRch4so4.out),
                     Integrating_rate(mmRnh4o2.out),
                     Integrating_rate(mmRtso2.out),
                     Integrating_rate(mmRno3ts.out),
                     Integrating_rate(mmRch4no3.out))
df_flux2 = data.frame(t(df_flux))
df_flux2$cat            =            c("OM.O2_flux",            "OM.DNF_flux",
"OM.DNRA_flux","OM.SO4_flux","OM.CH4_flux",
                "CH4_O2_flux","CH4_SO4_flux",
                "NH4_O2_flux","TS_O2_flux","TS_NO3_flux","CH4_NO3_flux")
df_flux2$t.df_flux.
df_flux2
# Save results
df_flux2_mature_seep_50_100cm = df_flux2
# Set the levels
df_flux2 = arrange(df_flux2, desc(t.df_flux.))
level <- df_flux2[,2]
df_flux2$cat <- factor(df_flux2$cat,level = rev(level))
# Plot bar
p <- ggplot(df_flux2)+
  geom_bar(aes(x = cat, y = t.df_flux., fill = t.df_flux.),
           stat = 'identity', position = 'stack', show.legend=TRUE)+
  ylab("Flux mmol m-2 d-1")+
  xlab("")+
  coord_flip()+
  scale_y_log10()
p
```
