# Supplementary material for: Rapid de novo assembly of animal-microbe biofilter to mitigate seabed methane leakage
Source: Natl Sci Rev. 2026 May 14;13(12):nwag266. doi: 10.1093/nsr/nwag266 (PMC13296563; doi:10.1093/nsr/nwag266)
Supplement: nwag266_Supplemental_Files [file nwag266_supplemental_files.zip › Supplementary Data.pdf]

# Supplementary Data for

# Rapid *de novo* assembly of animal-microbe biofilter to mitigate seabed methane leakage

**The supplementary data file includes:**

Supplementary Text 1: Materials and Methods

Supplementary Figs. 1 – 11

Supplementary Tables. 1 – 5

Supplementary references

# Supplementary Text 1: Materials and Methods

**3D seismic survey**

High-resolution 3D seismic survey was conducted by China National Offshore Oil Corporation in the Songnan Low uplift of Qiongdongnan Basin, South China Sea (SCS), to identify the seismic response characteristics associated with subseafloor geological structure, fluid migration, and gas hydrate accumulation. The data were acquired using 12 parallel streamers with 100 m spacings, with inline (NW-SE) and crossline (SW-NE) spacings of 12.5 m and 12.5 m, respectively, and 1.0 ms sampling interval. The data were processed as previously described [1].

**Multibeam echosounder survey**

Annual hydroacoustic surveys were conducted to track the activity of methane discharge from seafloor in the study region. Acoustic backscatter data from mid-water reflectors were collected using a shipborne Kongsberg EM302 multibeam echosounder, operating at a nominal frequency of 30 kHz with a swath width of 120°. The acoustic backscatter data were processed using CARIS 8.1 software to identify and extract gas flares.

**Macrofaunal analyses**

Macrofaunal identification and quantification were performed using a sediment sieving-based method as previously described [2], complemented by the image-based analysis. Sediments from box or push cores were sieved through 0.5-mm mesh, the retained material was then fixed in 4% formaldehyde. Faunas were separated from debris in the laboratory and stored in 70% ethanol for later counting and identification. Only few copepods can be retrieved by the sieving method despite their relatively high abundances recorded by underwater camera. The abundance of these faunal species therefore was estimated by analyses on images/videos that clearly visualized the individuals of copepods.

**Geochemical analyses**

Methane concentrations were measured using a gas chromatograph (Shimadzu) equipped with a Barrier Discharge Ionization Detector. DIC concentration and its carbon isotopic ratio ($\delta^{13}C_{DIC}$) were determined using Gas Bench II coupled with a Stable Isotope Ratio Mass Spectrometer (Delta 253plus, Thermo Scientific). Hydrogen sulfide was measured by methylene blue spectrophotometry (DR5000, Hach), while sulfate was quantified by ion chromatography (Dionex ICS-5000+, Thermo Scientific). $NO_3^-$, $NO_2^-$ and $NH_4^+$ were measured by using a continuous flow Auto Analyzer (AA3, SEAL). TOC, TN, and $\delta^{13}$C-TOC were determined after removing the inorganic carbon through vaporization with concentrated HCl, using an elemental analyzer (Vario EL III) coupled to an isotope ratio mass spectrometer (Isoprime, Elementar) at the Instrumental Analysis Center of Shanghai Jiao Tong University.

**Time-dependent reaction-transport model**

To model the reaction rates and fluxes of dissolved chemical species in sediments, the evolution of porewater profiles over time is simulated using a 1D time-dependent

reaction transport model:

$$\frac{\partial \phi C}{\partial t} = -\frac{\partial}{\partial x}\left(\phi D \frac{\partial C}{\partial x} - \phi u C\right) + \alpha\phi(C_0 - C) + \sum R$$

where $C$ is the solute concentration (M $L^{-3}_{fluid}$), $t$ is time, $\phi$ is porosity, $D$ is the tortuosity-corrected diffusion coefficient[2], $u$ is the advection velocity, $a$ is a nonlocal transport coefficient, $C_0$ is the concentration in the overlying water, and $\Sigma R$ is the net rate of production or consumption of chemical $C$ (M $L^{-3}_{total}$ $T^{-1}$). The model takes into consideration dissolved oxygen, sulfate, methane, nitrate, ammonium, total sulfide, and dissolved inorganic carbon. Their concentrations are fixed at the upper boundary representing the overlying water ($O_2$ = 250 μM, $SO_4^{2-}$ = 29 mM, $NO_3^-$ = 40 μM, $NH_4^+$ = 0 μM, TS = 0 μM, DIC = 2.2 mM, $CH_4$ = 0 mM). The model accounts for reactions involved in the mineralization of organic matter ($R_{aer}$, $R_{DNF}$, $R_{DNRA}$, $R_{SR}$, $R_{MOG}$; M $L^{-3}_{total}$ $T^{-1}$) and secondary reaction ($R_{no3ts}$, $R_{sox}$, $R_{nitri}$, $R_{ch4o2}$, $R_{aom}$, $R_{no3ch4}$; M $L^{-3}_{fluid}$ $T^{-1}$) listed in Supplementary Table 5. The rate parameters are primarily based on Wang and Van Cappellen (1996) [3]. The organic matter mineralization rate $R_C$ is imposed as a function of depth, $R_C = R_C^0\, e^{(-\max(0, x - x_m)/x_{att})}$, with $R_C^0$ = 0.6 mol $m^{-3}$ $yr^{-1}$. The irrigation coefficient was given the same functional depth dependence i.e., $\alpha = \alpha_0\, e^{(-\max(0, x - x_{irr})/x_{attirr})}$, with $\alpha_0$= 200 $yr^{-1}$ was set [4].

Model simulations were carried out for a multi-year period capturing the evolution of the porewater profiles, using the R package "ReacTran" v1.4.3. First, steady state profiles were simulated during non-seep conditions, with $u = u_{compaction} = v_\infty\, \phi_\infty/\phi$, where $v_\infty$ is the sedimentation rate solids at depth (set to 2 mm/yr). Mixing was set to be relatively shallow with $x_{irr}$ = 0.03 m and $x_{attirr}$ = 0.01 m, and mineralization rates decayed below the shallow mixing depth ($x_m$ = 0.03 m; $x_{att}$ = 0.01 m). These porewater profiles were then used as initial conditions for a 10-year simulation (Newborn Seep stage) with seepage, deeper solute mixing ($x_{irr}$ = 0.3 m and $x_{attirr}$ = 0.1 m) and mineralization extending deeper reflecting the effect of deep bioturbation ($x_m$ = 0.3 m), to reflect the deeper mixed layer (Fig. 4). Subsequently, simulations were run toward steady state with continued seepage, but reduced mixing $x_{irr}$ = 0.03 m and $x_{attirr}$ = 0.01 m) and shallower mineralization ($x_m$ = 0.03 m), representing the conditions found in nearby mature seeps. In the absence of seepage, a no concentration gradient condition was imposed at the lower domain boundary at a sediment depth of 2 m. During seepage, a flux was imposed as $F = \phi u C_{seep}$, where $u = u_{compaction} + u_{seepage}$, with $u_{seepage}$ = -0.25 m/yr. The seepage concentrations $C_{seep}$ were set to $O_2$ = 0 μM, $SO_4^{2-}$ = 0 mM, $NO_3^-$ = 0 μM, $NH_4^+$ = 20.2 μM, TS = 860 μM, DIC = 2.3 mM, $CH_4$ = 50 mM. In addition, during seepage periods, it was assumed that a small amount of methane can partition from the rising gas phase into the porewater, adding to the dissolved methane concentration depending on the extent of undersaturation of the dissolved methane: $R_g = k_g$*($[CH_4]_{seep}$ – $[CH_4]$), with $k_g$ arbitrarily set to 0.044 $yr^{-1}$.

## Monod biomass-explicit model

Methane oxidation rates were also calculated using the Monod biomass-explicit model, assuming the constant coupling between cell growth rate and specific substrate oxidation rate (i.e., there is no temporary uncoupling or 'unproductive' oxidation) [5]:

$$V = u \cdot {Y_{mol}}^{-1}$$

where $V$ is the specific rate of substrate oxidation (mol substrate oxidized per cell dry weight and time). $u$ is the net cell growth rate, estimated based on the temporal changes in abundances of specific methanotrophic lineages (details see the method section "*In situ* cell growth"). $Y_{mol}$ is the molar growth yield, was set to 0.6 g cell dry weight per mol $CH_4$ oxidized according to the experiment data of typical methane seep sediments [5]. The derived specific rate of substrate oxidation $V$ was further converted to flux based on the mean abundances of methanotrophic lineages ($10^7$–$10^8$ cell $g^{-1}$) and depth integration.

**Isotopic labeling experiments**

Potential rates of denitrification and dissimilatory nitrate reduction to ammonium (DNRA) were measured with $^{15}N$ tracer slurry incubations as previously described [6, 7]. Briefly, the fauna-free sediments were 1:7 diluted with artificial seawater, purged with helium for 30 min and then distributed into 12-mL Exetainers that were filled without headspace. The vials were preincubated at *in situ* temperature for 24 h and then spiked with $^{15}NO_3^-$ to a final concentration of ~100 μM. Half vials were immediately treated with 200 μL 50% $ZnCl_2$ solution to terminate the reaction and sampled as time zero, while the remaining vials were sampled after 24 hours. The rates of denitrification and DNRA were calculated from $^{30}N_2$ and $^{15}NH_4^+$ generation within the vials, respectively, which were measured by membrane inlet mass spectrometry and by micro-diffusion method [8]. The coupled rates of nitrate reduction to methane and sulfide oxidation were calculated by comparing the treatments of $NO_3^-$+$CH_4$ and $NO_3^-$+$S^{2-}$ with control ($NO_3^-$), respectively.

**Nucleic acids extraction and 16S rRNA gene sequencing**

Genomic DNA was extracted from 0.5-2.0 g sediments and DNA-free water (blank control) using a modified SDS-based extraction method [9]. For 16S rRNA gene sequencing, the hypervariable V4 region of 16S rRNA genes was amplified using the primer pair 515F (5'-GTGYCAGCMGCCGCGGTAA-3') / 806R (5'-GGACTACNVGGGTWTCTAAT-3') [10]. The consistency of dominant microbial groups was further verified by applying the primer pairs of 338F (5′-ACTCCTACGGGAGGCAGCAG-3′) / 806R (5′-GGACTACHVGGGTWTCTAAT-3′) and Arch344F (5′-ACGGGGYGCAGCAGGCGCGA-3′) / Arch915R (5′-GTGCTCCCCCGCCAATTCCT-3′) to selected samples. Sequencing was performed using the Illumina Miseq platform (Illumina, USA) as previously described [10, 11]. The raw reads of V4 region of 16S rRNA gene were processed and analyzed using the QIIME 2 platform v2020.11 [12]. The primers and adapters were first trimmed out using Cutadapt v3.1 [13]. Raw sequences were then processed using DADA2 [14], including quality filtering, denoising, paired-end sequence merging, chimera filtering and producing amplicon sequence variants (ASVs) and ASV Table. Taxonomy was assigned using q2-featureclassifier (a scikit-learn naive Bayes machine-learning classifier) [15] with Silva database release 138 [16]. Multiple sequence alignment and phylogenetic tree construction were performed using the QIIME 2 plugin q2phylogeny (align-to-tree-mafft-iqtree). Unassigned sequences, singletons and sequences affiliated

with eukaryotes were discarded.

**Metatranscriptomic analysis**

For metatranscriptomic sequencing, RNA was extracted from ~5 g sediments using the RNeasy PowerSoil Total RNA Kit (QIAGEN, Germany). After removing ribosomal RNA and DNA by the Ribo-off rRNA depletion kit (Vazyme, China), libraries were prepared using the TruePrep RNA Library Prep Kit and sequenced on the Illumina NovaSeq-PE150 platform at the Personalbio (Shanghai, China). Metatranscriptomic reads were first quality filtered using Trimmomatic v 0.38 [17] and mRNA sequences were obtained by removing rRNA sequences with SortMeRNA v 4.3.6 [18] against both the SILVA [16] and the default databases. Sam files were generated by mapping mRNA sequences to MAGs using hisat2 v 2.2.1 [19]. Gene transcription was calculated by counting the number of unambiguously mapped reads for each gene using featureCount [20]. To compare transcription levels between genes, read counts were converted to TPM. A metabolic network at the transcriptional level was constructed based on the pairwise Spearman correlations between genes. Only correlations with $p<0.01$ were shown in the network. The taxonomic information of each gene was further annotated with the NR database using diamond blastp according to the best-hit target and visualized using the "circlify" package in Python (www.python.org).

**Quantitative PCR**

Abundances of bacterial and archaeal 16S rRNA genes were quantified on a StepOne Plus (Applied Biosystems, USA) by SYBR-Green I-based quantitative PCR (qPCR). The primer pairs for Bacteria and Archaea were bac341f (5'-CCTACGGGWGGCWGCA-3') / 519r (5'-TTACCGCGGCKGCTG-3') and Uni519f (5′-CAGCMGCCGCGGTAA-3′) / Arc908R (5′-CCCGCCAATTCCTTTAAGTT-3′) [21, 22], respectively. The reaction mixture (20 µl) included 10 µl of ChamQ SYBR Color qPCR Master Mix (Vazyme, China), 1.6 µl (Bacteria) / 1.6 µl (Archaea) of each primer (10 µM), and 1 µl of template DNA. The thermal cycling program was: initial denaturation at 95 °C for 15 min, 40 cycles at 95 °C for 30 s, 56 °C (Bacteria) / 60 °C (Archaea) for 30 s, and 72 °C for 30 s. Plasmids of 16S rRNA genes from *Marinobacter* sp. and *Bathyarchaeota* were used as bacterial and archaeal standards. Microbial cell abundance was subsequently estimated using the average 16S gene copies per cell (Bacteria: $4.12 \pm 2.75$ copies $cell^{-1}$; Archaea: $1.61 \pm 0.88$ copies $cell^{-1}$) based on the rrn database (https://rrndb.umms.med.umich.edu/).

**Statistics**

All statistical analyses were performed in R (www.Rproject.org). Microbial alpha-diversity indices and PCoA coordinates using Bray-Curtis distances were calculated using the "phyloseq" package [23]. Using the "vegan" package [24], we performed: 1) Anosim analysis to examine the community (dis)similarity between different groups of samples, 2) PERMANOVA analysis to examine the powers of different categorical and geochemical factors in explaining community variations, 3) two-sided Welch's t test with Bonferroni-Holm correction to compared means between two independent groups without assuming equal population variances. The succession rates of microbial communities were calculated as previously described, by calculating the temporal

variations of the community distances measured by both Bray-Curtis and Sorensen metrics with time [25]. The potential contribution of methane derived carbon to the faunal diet was calculated by the "MixSIAR" pakage, using the default uninformative prior and Markov chain Monte Carlo settings [26].

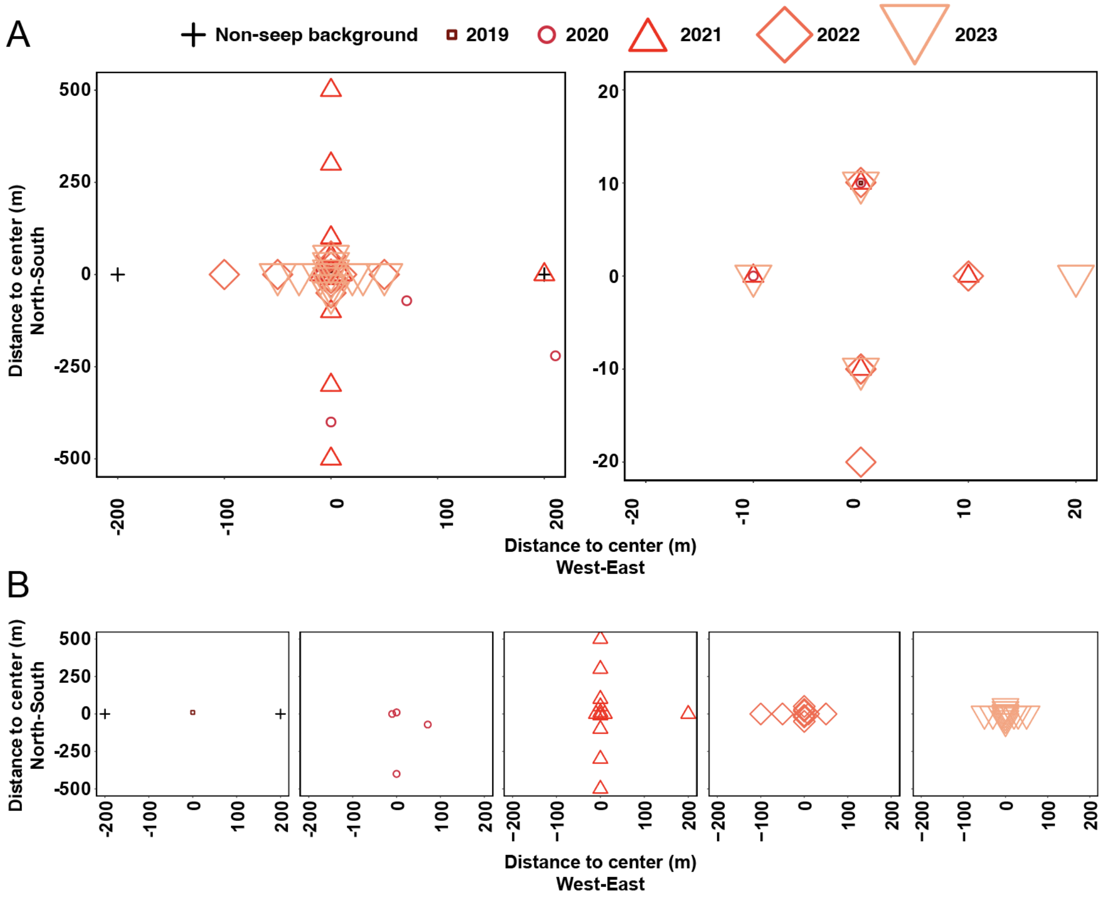


**Supplementary Fig. 1.** Information of the sampling sites around the Newborn Seep, with coordinate (0, 0) denotes the discharge center. **(A)** All sampling sites viewed at different spatial scales. **(B)** Yearly sampling sites from 2019-2023. The non-seep background sites 19-NS-BG1 and 19-NS-BG2 were taken ~200 m away from the discharge center of Newborn Seep in 2019, before the spreading of leakage on seafloor.

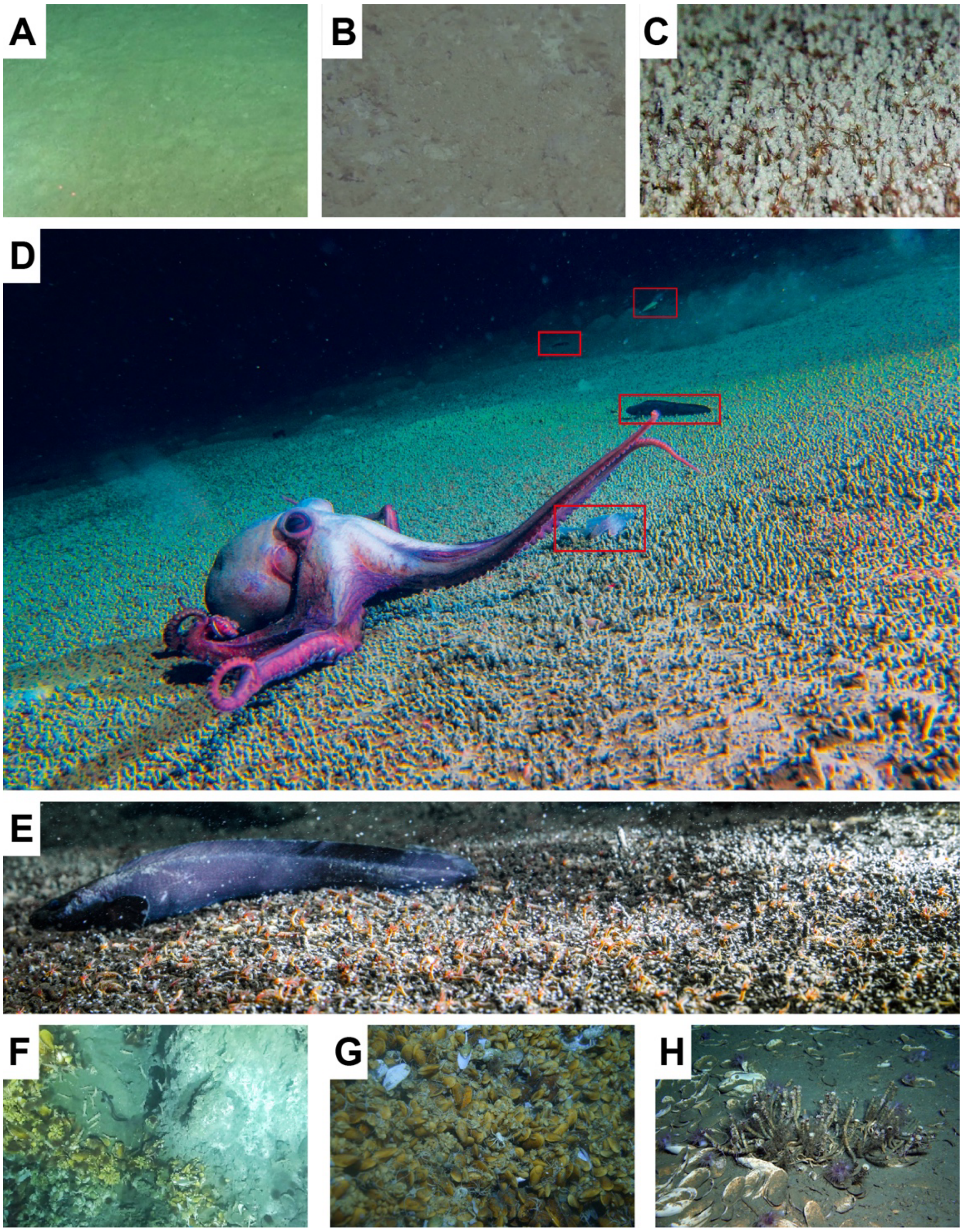


**Supplementary Fig. 2.** In-situ ROV images showing active methane venting and associated seep fauna at multiple sites in the Qiongdongnan Basin (QDN) and Haima region. **(A-B)** the nearly 'bare' seafloor observed before (2018) and after the methane leakage in 2019, respectively; **(C)** a dense assemblage of polychaetes blanketing the soft sediments of the Newborn Seep observed in 2021; **(D-E)** higher trophic animals attracted by the newly formed biomass-rich ecosystem; **(F)** carbonate outcrops, white microbial mats, and mussel aggregations indicative of active seepage at the mature seep site S18; **(G-H)** typical mature seep fauna including the mussels, tubeworms, and clams at the mature "Haima" cold seeps.

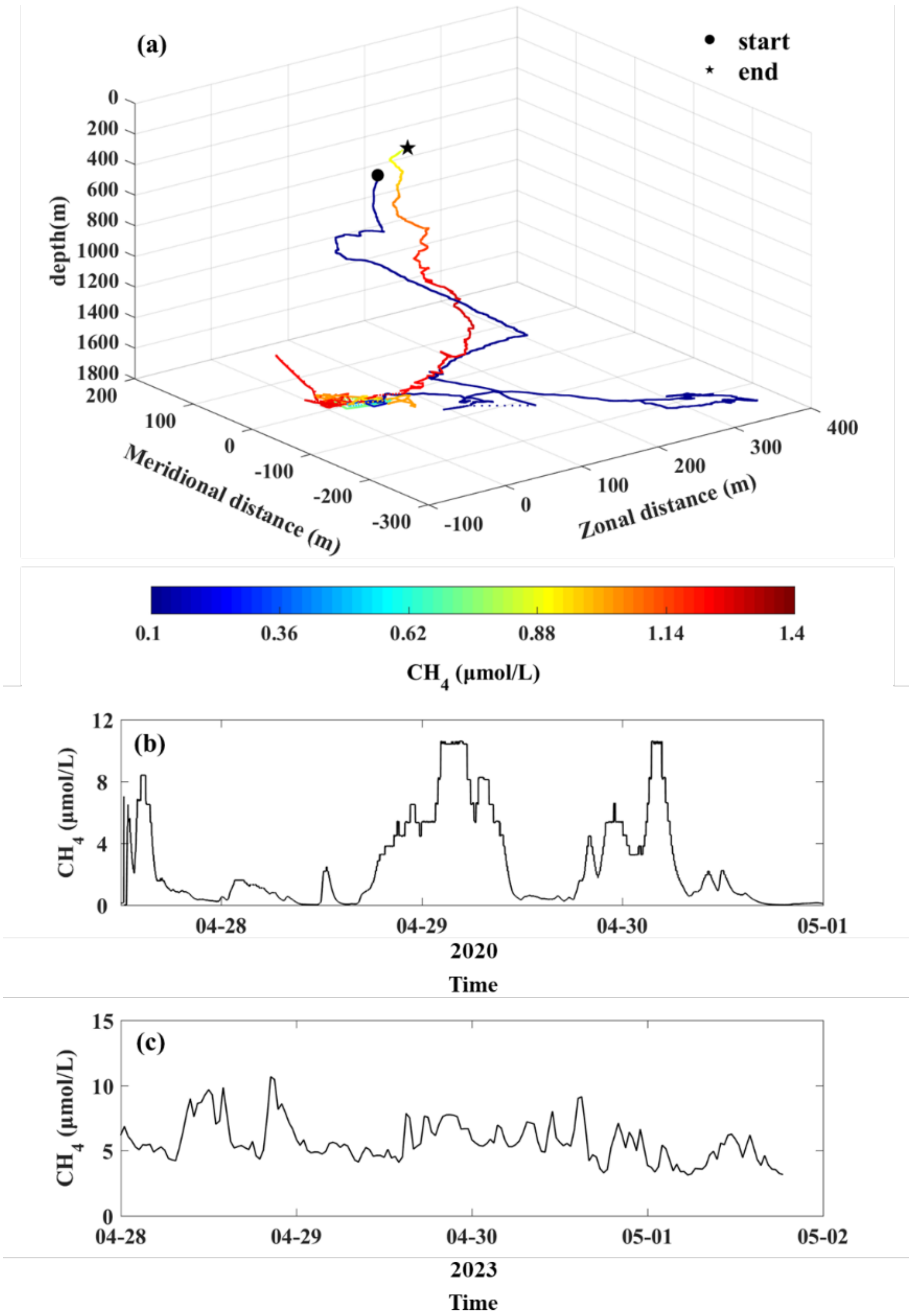


**Supplementary Figure 3.** Temporal variation of seawater methane concentrations at the Newborn Seep site: **(a)** Three-dimensional plot of the sampling path, with meridional distance (m), zonal distance (m), and depth (m) on the axes. The color scale indicates measured $CH_4$ concentrations (μmol/L). The black symbols (circle, star) denote the start and end points of the survey. **(b)** Time series of methane concentrations ($CH_4$, μmol/L) recorded in water column above the discharge center, from April 27 to May 1, 2020. **(c)** Time series of methane concentrations ($CH_4$, μmol/L) recorded in water column above the discharge center, during a separate campaign in 2023 (April 28 to May 2).

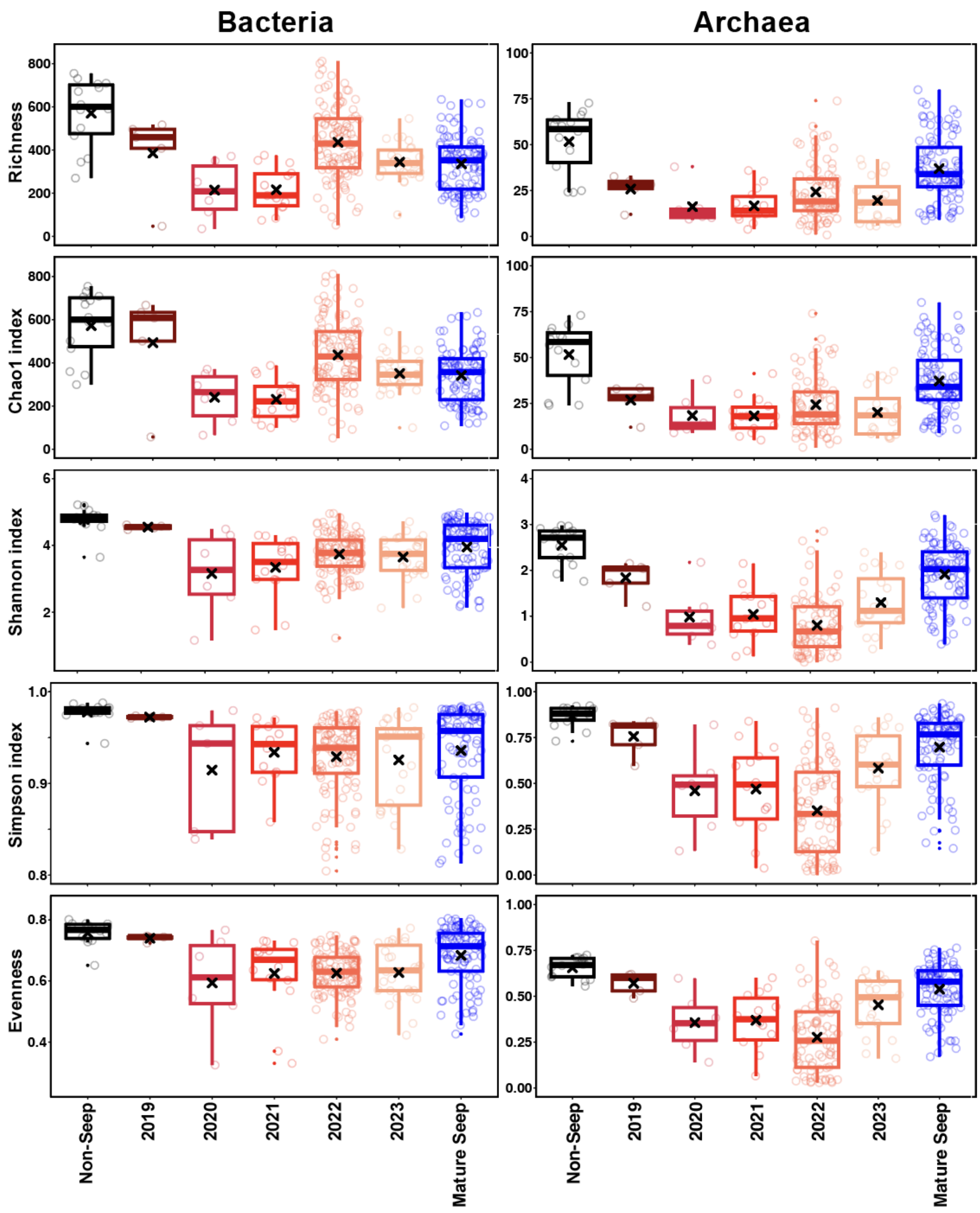


**Supplementary Figure 4. Multi-year variations of the bacterial and archaeal diversity indices following methane leakage.** The 'non-seep seafloor' group include data from the adjacent non-seep background sites, while the 'mature seep' group include data from the 'Haima' and 'Site F' cold seeps in the South China Sea (see Supplementary Table 1).

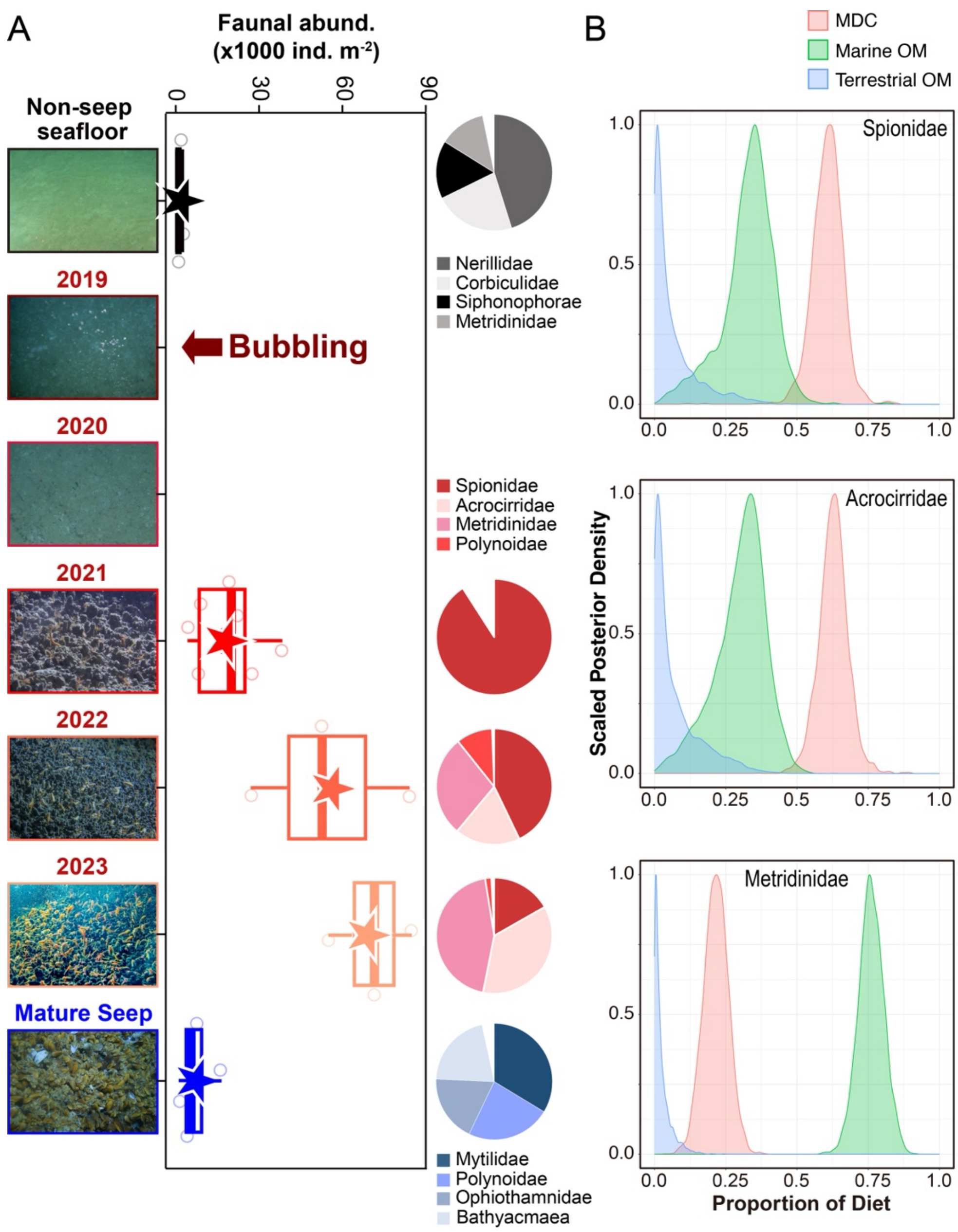


**Supplementary Fig. 5. Rapid proliferation of faunal populations after methane leakage. (A)** Time-series changes in benthic faunal abundance ($\times 10^3$ individuals $m^{-2}$) from a non-seep seafloor reference (top) through multiple post-discharge years (2019-2023) to a mature seep environment (bottom). The boxplots display medians, quartiles, and data ranges. The dots within boxplots denote the mean values. The pie charts illustrate the relative abundances of major faunal groups. Active bubbling from the seafloor was first observed in 2019, in line with the onset of methane discharge detected by multibeam echosounder. **(B)** Bayesian stable isotope mixing model results showing the proportional contributions of three dietary carbon sources—methane-derived carbon (MDC), marine organic matter (Marine OM), and terrestrial organic

matter (Terrestrial OM)—to major fauna groups (Spionidae, Acrocirridae, Metridinidae) residing at the Newborn Seep. The x-axis and y-axis denote the proportional contribution of each carbon source to the faunal diet and the scaled posterior density, respectively.

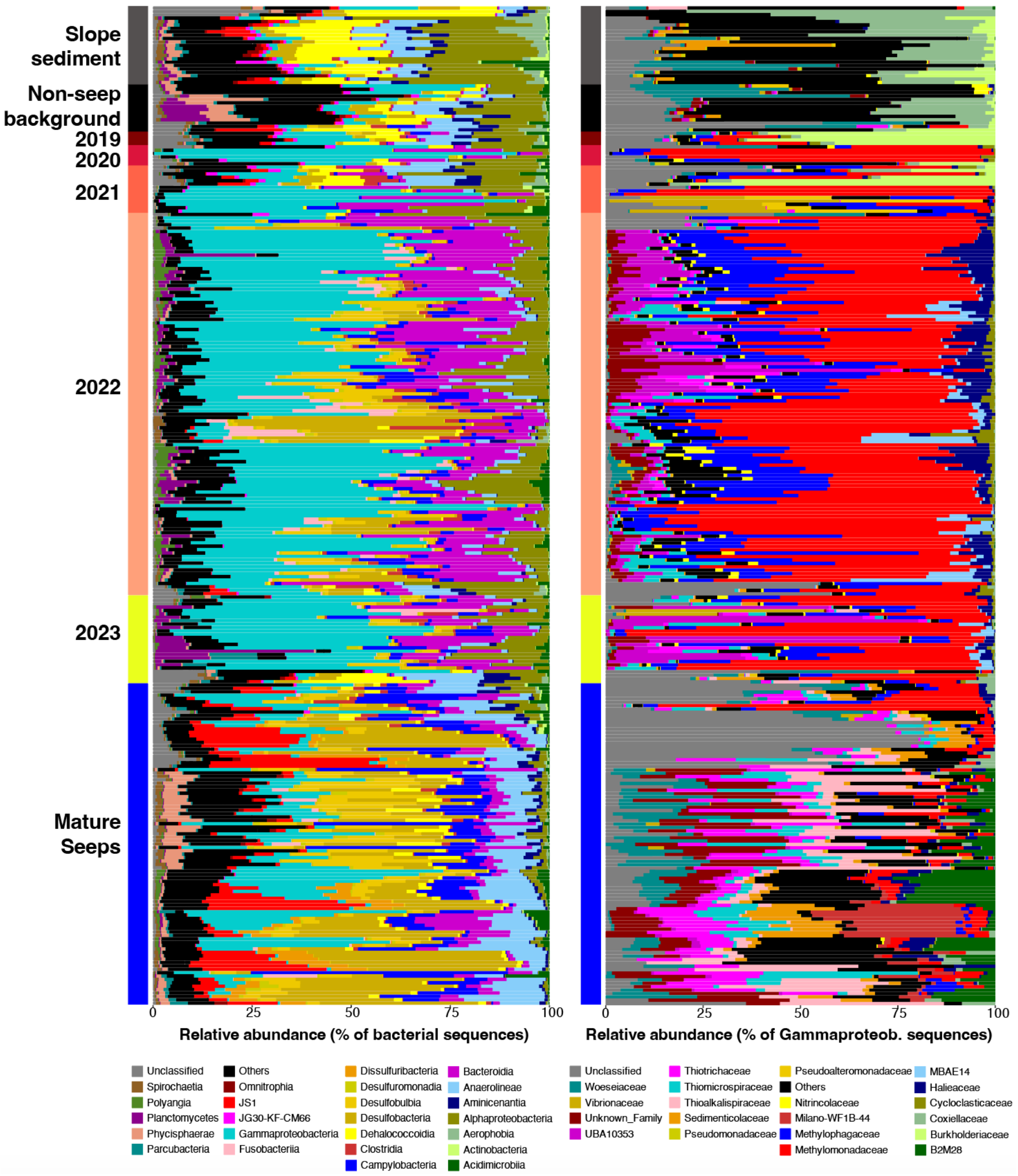


**Supplementary Fig. 6.** Class-level bacterial community compositions (left) and family-level community compositions of *Gammaproteobacteria* (right) in sediments across the continental slope, non-seep background, Newborn Seep (2019-2023), and mature seep sites.

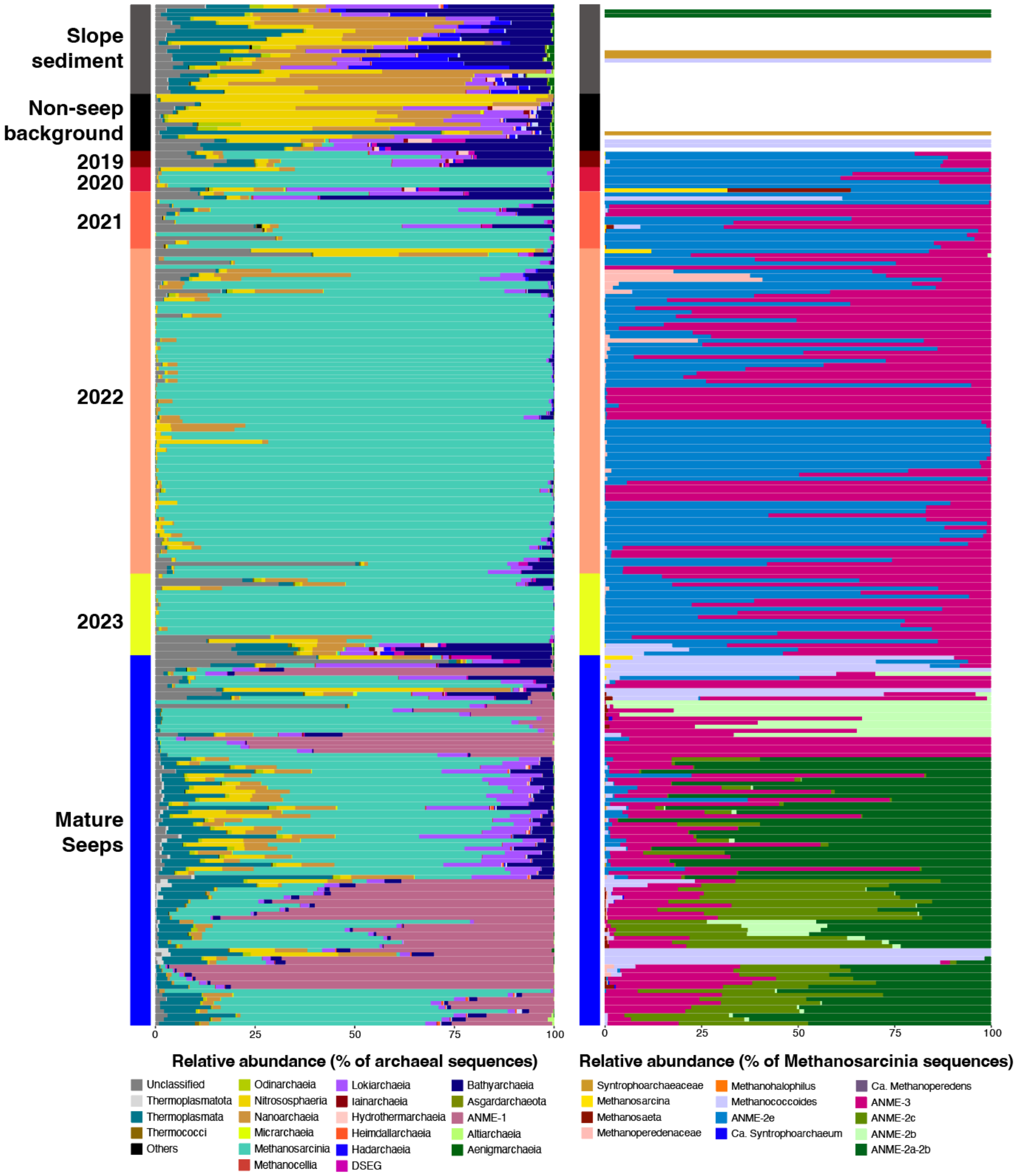


**Supplementary Fig. 7.** Class-level archaeal community compositions (left) and family-/genus-level community compositions of *Methanosarcinia* (right) in sediments across the continental slope, non-seep background, Newborn Seep (2019-2023), and mature seep sites.

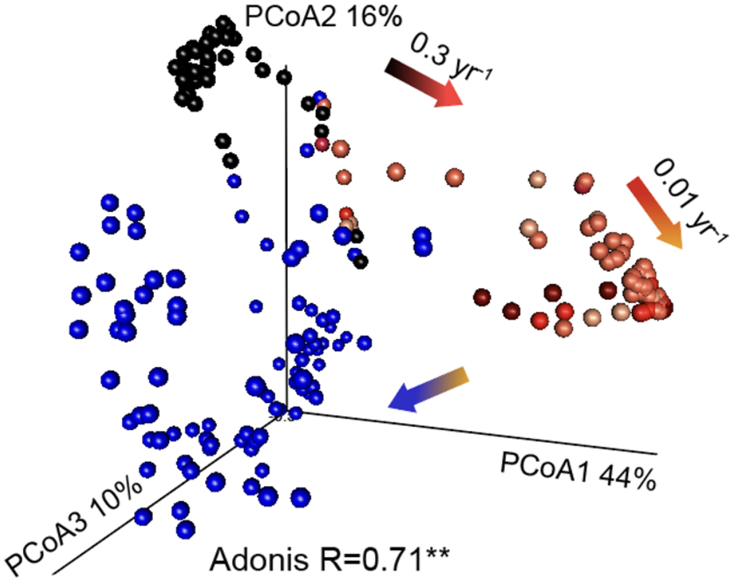


**Supplementary Fig. 8.** Principal coordinates analysis (PCoA) plots based on archaeal 16S rRNA genes, demonstrating distinct archaeal community clustering by seep stage and year. The Adonis R and p-values indicate the proportion of variation explained by seep stage (**: $p$<0.01).

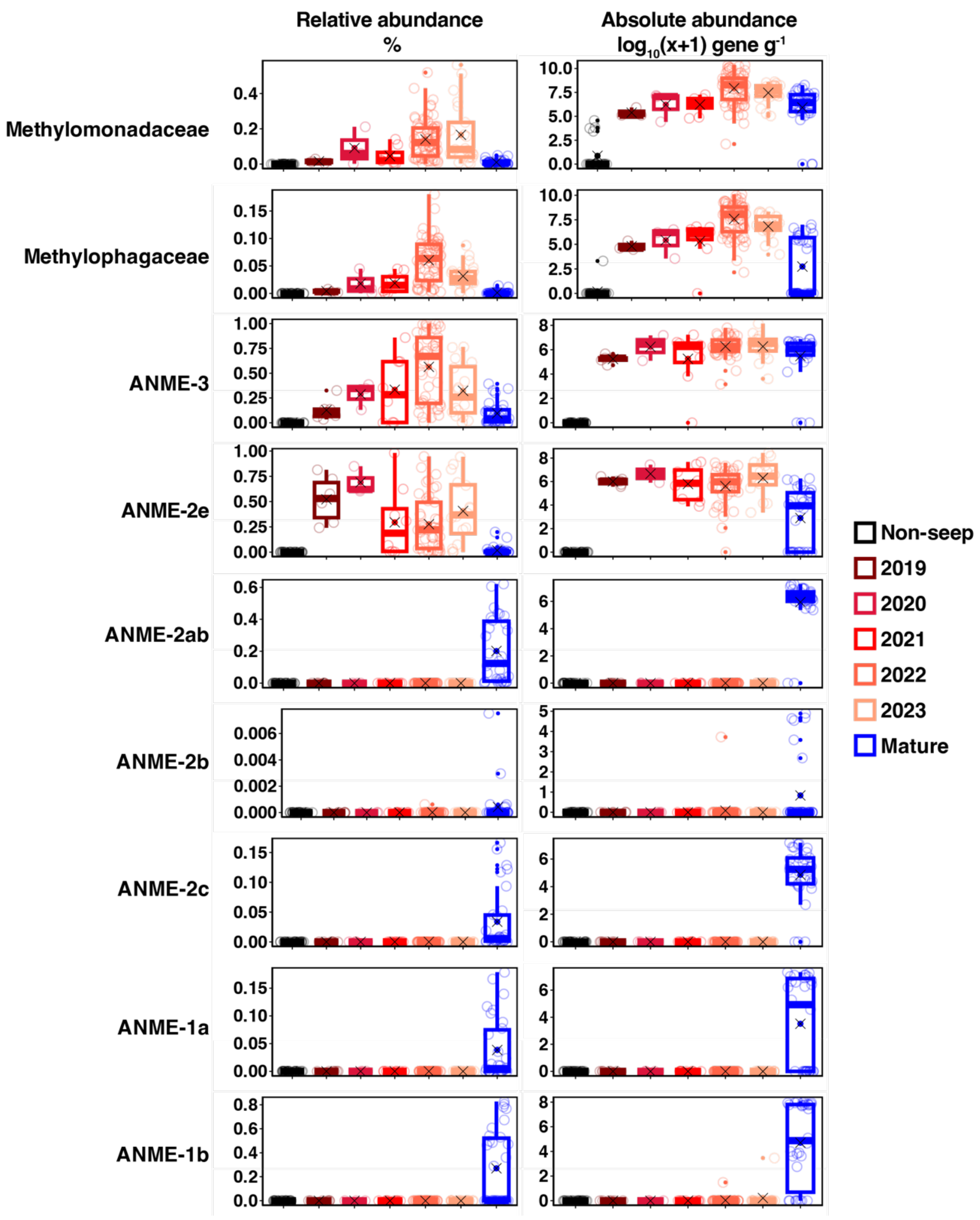


**Supplementary Fig. 9.** Changes of relative and absolute abundances over time show the succession of dominant aerobic and anaerobic methanotrophic lineages across the non-seep, Newborn Seep, and mature seep stages.

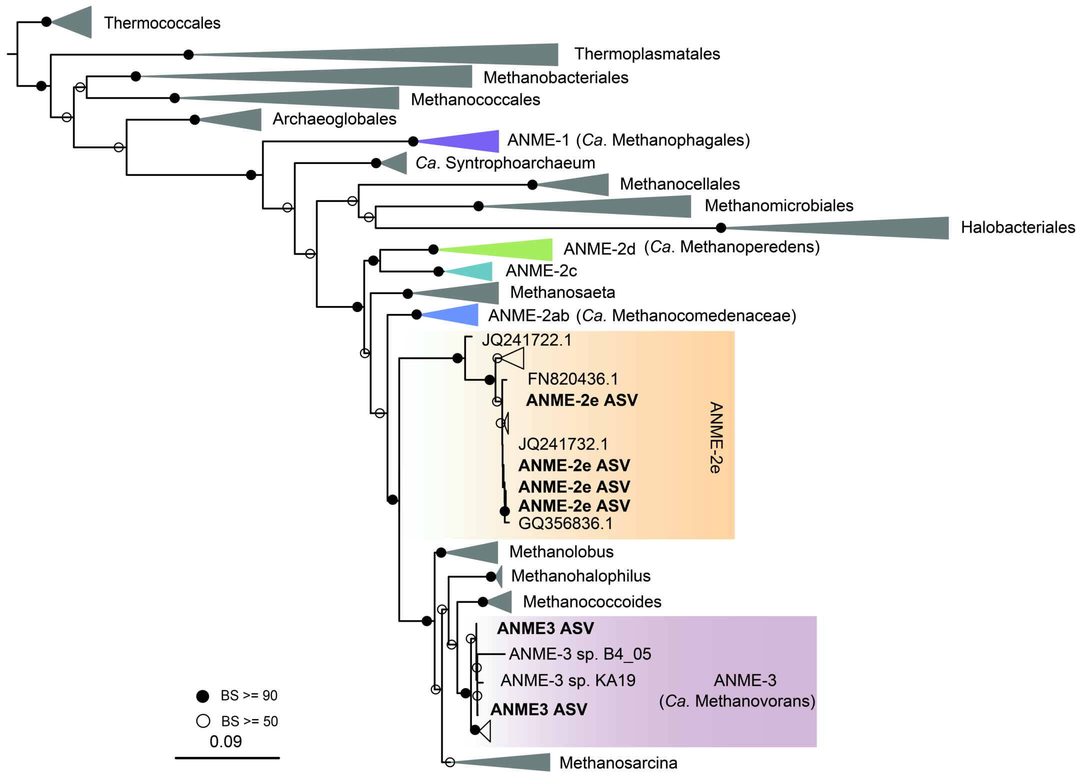


**Supplementary Fig. 10.** The phylogenetic tree of 16S rRNA gene sequences extracted from ANME MAGs and ANME ASVs. The tree was constructed in IQ-TREE using the SYM+I+I+R5 model with 1,000 ultrafast bootstraps. Black and white dots indicate ≥90% and ≥50% bootstrap values, respectively. 16S rRNA genes sequences from this study are shown in bold letters.

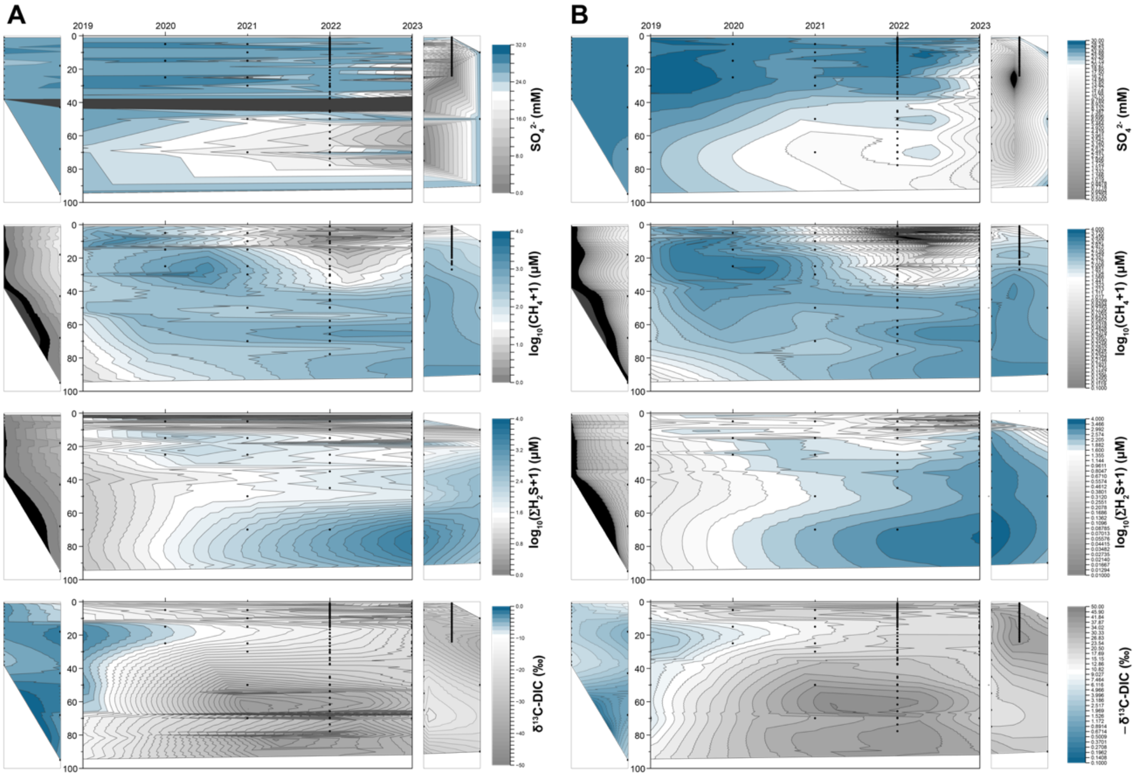


**Supplementary Fig. 11.** Contour plots of methane concentration ($CH_4$), $\delta^{13}C$ of dissolved inorganic carbon ($\delta^{13}C$-DIC), sulfate concentration ($SO_4^{2-}$), and total concentrations of hydrogen sulfide ($\sum H_2S$) in 0-100 cm of the Newborn Seep sediments using the (**A**) linear model without the incorporation of optimal-smooth function and (**B**) probability model. The results suggest consistent overall trends with those shown in Fig. 4A using the linear model with the incorporation of optimal-smooth function.

**Supplementary Table 1.** Sample information. Nomenclature of sample names: sampling year_sampling site_sediment depth cm.

| SampleID[1] | Water_m | Distance to center_m | Geochemistry | 16S sequencing | Metatranscriptomic Sequencing | Sample type | Source |
|---|---|---|---|---|---|---|---|
| 19 C12_11.5 | 1897 | >100,000 | √ | √ | | Gravity/piston core | This study |
| 19 C12_21.5 | 1897 | >100,000 | √ | √ | | Gravity/piston core | This study |
| 19 C12_31.5 | 1897 | >100,000 | √ | √ | | Gravity/piston core | This study |
| 19 C12_41.5 | 1897 | >100,000 | √ | √ | | Gravity/piston core | This study |
| 19 C12_61.5 | 1897 | >100,000 | √ | √ | | Gravity/piston core | This study |
| 19 C12_81.5 | 1897 | >100,000 | √ | √ | | Gravity/piston core | This study |
| 19 C12_101.5 | 1897 | >100,000 | √ | √ | | Gravity/piston core | This study |
| 19 C12_141.5 | 1897 | >100,000 | √ | √ | | Gravity/piston core | This study |
| 19 C12_181.5 | 1897 | >100,000 | √ | √ | | Gravity/piston core | This study |
| 19 C12_221.5 | 1897 | >100,000 | √ | √ | | Gravity/piston core | This study |
| 19 C12_261.5 | 1897 | >100,000 | √ | √ | | Gravity/piston core | This study |
| 19 C12_281.5 | 1897 | >100,000 | √ | √ | | Gravity/piston core | This study |
| 19 C12_301.5 | 1897 | >100,000 | √ | √ | | Gravity/piston core | This study |
| 19 C12_341.5 | 1897 | >100,000 | √ | √ | | Gravity/piston core | This study |
| 19 C12_381.5 | 1897 | >100,000 | √ | √ | | Gravity/piston core | This study |
| 19 C12_421.5 | 1897 | >100,000 | √ | √ | | Gravity/piston core | This study |
| 19 C12_461.5 | 1897 | >100,000 | √ | √ | | Gravity/piston core | This study |
| 19 C12_501.5 | 1897 | >100,000 | √ | √ | | Gravity/piston core | This study |
| 19 C12_531.5 | 1897 | >100,000 | √ | √ | | Gravity/piston core | This study |
| 19 C12_561.5 | 1897 | >100,000 | √ | √ | | Gravity/piston core | This study |
| 19 C73_6.5 | 894 | >100,000 | √ | √ | | Gravity/piston core | This study |

| | | | | | | | |
|---|---|---|---|---|---|---|---|
| 19 C73_21.5 | 894 | >100,000 | √ | √ | | Gravity/piston core | This study |
| 19 C73_31.5 | 894 | >100,000 | √ | √ | | Gravity/piston core | This study |
| 19 C73_41.5 | 894 | >100,000 | √ | √ | | Gravity/piston core | This study |
| 19 C73_61.5 | 894 | >100,000 | √ | √ | | Gravity/piston core | This study |
| 19 C73_81.5 | 894 | >100,000 | √ | √ | | Gravity/piston core | This study |
| 19 C73_96.5 | 894 | >100,000 | √ | √ | | Gravity/piston core | This study |
| 19 C73_141.5 | 894 | >100,000 | √ | √ | | Gravity/piston core | This study |
| 19 C73_181.5 | 894 | >100,000 | √ | √ | | Gravity/piston core | This study |
| 19 C73_221.5 | 894 | >100,000 | √ | √ | | Gravity/piston core | This study |
| 19 C73_261.5 | 894 | >100,000 | √ | √ | | Gravity/piston core | This study |
| 19 C73_296.5 | 894 | >100,000 | √ | √ | | Gravity/piston core | This study |
| 19 C73_341.5 | 894 | >100,000 | √ | √ | | Gravity/piston core | This study |
| 19 C73_381.5 | 894 | >100,000 | √ | √ | | Gravity/piston core | This study |
| 19 C73_421.5 | 894 | >100,000 | √ | √ | | Gravity/piston core | This study |
| 19 C73_461.5 | 894 | >100,000 | √ | √ | | Gravity/piston core | This study |
| 19 C73_496.5 | 894 | >100,000 | √ | √ | | Gravity/piston core | This study |
| 19 C73_541.5 | 894 | >100,000 | √ | √ | | Gravity/piston core | This study |
| 19 C82_6.5 | 3220 | >100,000 | √ | √ | | Gravity/piston core | This study |
| 19 C82_11.5 | 3220 | >100,000 | √ | √ | | Gravity/piston core | This study |
| 19 C82_21.5 | 3220 | >100,000 | √ | √ | | Gravity/piston core | This study |
| 19 C82_31.5 | 3220 | >100,000 | √ | √ | | Gravity/piston core | This study |
| 19 C82_41.5 | 3220 | >100,000 | √ | √ | | Gravity/piston core | This study |
| 19 C82_61.5 | 3220 | >100,000 | √ | √ | | Gravity/piston core | This study |
| 19 C82_76.5 | 3220 | >100,000 | √ | √ | | Gravity/piston core | This study |
| 19 C82_101.5 | 3220 | >100,000 | √ | √ | | Gravity/piston core | This study |

| | | | | | | |
|---|---|---|---|---|---|---|
| 19 C82_121.5 | 3220 | >100,000 | √ | √ | Gravity/piston core | This study |
| 19 C82_141.5 | 3220 | >100,000 | √ | √ | Gravity/piston core | This study |
| 19 C82_161.5 | 3220 | >100,000 | √ | √ | Gravity/piston core | This study |
| 19 C82_201.5 | 3220 | >100,000 | √ | √ | Gravity/piston core | This study |
| 19 C82_241.5 | 3220 | >100,000 | √ | √ | Gravity/piston core | This study |
| 19 C82_276.5 | 3220 | >100,000 | √ | √ | Gravity/piston core | This study |
| 19 C82_321.5 | 3220 | >100,000 | √ | √ | Gravity/piston core | This study |
| 19 C82_361.5 | 3220 | >100,000 | √ | √ | Gravity/piston core | This study |
| 19 C82_401.5 | 3220 | >100,000 | √ | √ | Gravity/piston core | This study |
| 19 C82_441.5 | 3220 | >100,000 | √ | √ | Gravity/piston core | This study |
| 19 C82_476.5 | 3220 | >100,000 | √ | √ | Gravity/piston core | This study |
| 19 NS BG1_5 | 1766 | >200 | | √ | Gravity/piston core | This study |
| 19 NS BG1_17.5 | 1766 | >200 | √ | √ | Gravity/piston core | This study |
| 19 NS BG1_42.5 | 1766 | >200 | √ | | Gravity/piston core | This study |
| 19 NS BG1_67.5 | 1766 | >200 | √ | √ | Gravity/piston core | This study |
| 19 NS BG1_95 | 1766 | >200 | √ | √ | Gravity/piston core | This study |
| 19 NS BG2_1 | 1766 | 200 | √ | √ | Push core | This study |
| 19 NS BG2_3 | 1766 | 200 | √ | √ | Push core | This study |
| 19 NS BG2_5 | 1766 | 200 | √ | √ | Push core | This study |
| 19 NS BG2_7 | 1766 | 200 | √ | √ | Push core | This study |
| 19 NS BG2_9 | 1766 | 200 | √ | √ | Push core | This study |
| 19 NS BG2_12 | 1766 | 200 | √ | √ | Push core | This study |
| 19 NS BG2_16 | 1766 | 200 | √ | √ | Push core | This study |
| 19 NS BG2_20 | 1766 | 200 | √ | √ | Push core | This study |
| 19 NS BG2_24 | 1766 | 200 | √ | √ | Push core | This study |

| | | | | | | |
|---|---|---|---|---|---|---|
| 19 NS BG2_28 | 1766 | 200 | √ | √ | Push core | This study |
| 19 NS BG2_36 | 1766 | 200 | √ | √ | Push core | This study |
| 19 N10 10 | 1766 | 10 | √ | √ | Push core | This study |
| 19 N10 30 | 1766 | 10 | √ | √ | Push core | This study |
| 19 N10 50 | 1766 | 10 | √ | | Push core | This study |
| 19 N10 70 | 1766 | 10 | √ | √ | Push core | This study |
| 19 N10 90 | 1766 | 10 | √ | √ | Push core | This study |
| 19 N10 10-2 | 1766 | 10 | | √ | Push core | This study |
| 19 N10 30-2 | 1766 | 10 | | √ | Push core | This study |
| 19 N10 70-2 | 1766 | 10 | | √ | Push core | This study |
| 19 N10 90-2 | 1766 | 10 | | √ | Push core | This study |
| 20 N10 1 | 1766 | 10 | √ | √ | Push core | This study |
| 20 N10 5 | 1766 | 10 | √ | √ | Push core | This study |
| 20 N10 15 | 1766 | 10 | √ | √ | Push core | This study |
| 20 N10 25 | 1766 | 10 | √ | √ | Push core | This study |
| 20 W10 5 | 1766 | 10 | √ | √ | Push core | This study |
| 20 W10 15 | 1766 | 10 | √ | √ | Push core | This study |
| 21 S10 5 | 1766 | 10 | √ | √ | Push core | This study |
| 21 S10 15 | 1766 | 10 | √ | √ | Push core | This study |
| 21 E10 10 | 1766 | 10 | √ | √ | Push core | This study |
| 21 E10 30 | 1766 | 10 | √ | √ | Push core | This study |
| 21 E10 50 | 1766 | 10 | √ | √ | Push core | This study |
| 21 E10 70 | 1766 | 10 | √ | √ | Push core | This study |
| 21 N10 5 | 1766 | 10 | √ | √ | Push core | This study |
| 21 N10 15 | 1766 | 10 | √ | √ | Push core | This study |

| | | | | | | | |
|---|---|---|---|---|---|---|---|
| 21 N10 25 | 1766 | 10 | √ | √ | | Push core | This study |
| 21 W10 1 | 1766 | 10 | | √ | | Push core | This study |
| 21 W10 4.5 | 1766 | 10 | | √ | | Push core | This study |
| 21 W10 7.5 | 1766 | 10 | | √ | | Push core | This study |
| 21 W10 10.5 | 1766 | 10 | | √ | | Push core | This study |
| 21 W10 16 | 1766 | 10 | | √ | | Push core | This study |
| 22 E10 5 | 1766 | 10 | √ | √ | | Push core | This study |
| 22 E10 15 | 1766 | 10 | √ | √ | | Push core | This study |
| 22 E10 25 | 1766 | 10 | √ | √ | | Push core | This study |
| 22 E10 35 | 1766 | 10 | √ | √ | | Push core | This study |
| 22 E10 45 | 1766 | 10 | √ | √ | | Push core | This study |
| 22 S10_0.25 | 1766 | 10 | √ | √ | √ | Push core | This study |
| 22 S10_0.75 | 1766 | 10 | √ | √ | | Push core | This study |
| 22 S10_1.25 | 1766 | 10 | √ | √ | | Push core | This study |
| 22 S10_1.75 | 1766 | 10 | √ | √ | | Push core | This study |
| 22 S10_2.25 | 1766 | 10 | √ | √ | | Push core | This study |
| 22 S10_2.75 | 1766 | 10 | √ | √ | | Push core | This study |
| 22 S10_3.25 | 1766 | 10 | √ | √ | | Push core | This study |
| 22 S10_3.75 | 1766 | 10 | √ | √ | | Push core | This study |
| 22 S10_4.25 | 1766 | 10 | √ | √ | | Push core | This study |
| 22 S10_4.75 | 1766 | 10 | √ | √ | | Push core | This study |
| 22 S10_5.25 | 1766 | 10 | √ | √ | | Push core | This study |
| 22 S10_5.75 | 1766 | 10 | √ | √ | | Push core | This study |
| 22 S10_6.25 | 1766 | 10 | √ | √ | | Push core | This study |
| 22 S10_6.75 | 1766 | 10 | √ | √ | | Push core | This study |

| | | | | | | | |
|---|---|---|---|---|---|---|---|
| 22 S10_7.25 | 1766 | 10 | √ | √ | | Push core | This study |
| 22 S10_7.75 | 1766 | 10 | √ | √ | | Push core | This study |
| 22 S10_8.25 | 1766 | 10 | √ | √ | | Push core | This study |
| 22 S10_8.75 | 1766 | 10 | √ | | | Push core | This study |
| 22 S10_9.25 | 1766 | 10 | √ | √ | | Push core | This study |
| 22 S10_9.75 | 1766 | 10 | √ | √ | | Push core | This study |
| 22 S10_10.5 | 1766 | 10 | √ | | √ | Push core | This study |
| 22 S10_11.5 | 1766 | 10 | √ | | | Push core | This study |
| 22 S10_12.5 | 1766 | 10 | √ | √ | | Push core | This study |
| 22 S10_13.5 | 1766 | 10 | √ | | | Push core | This study |
| 22 S10_14.5 | 1766 | 10 | √ | | | Push core | This study |
| 22 S10_16 | 1766 | 10 | √ | √ | | Push core | This study |
| 22 S10_18 | 1766 | 10 | √ | | | Push core | This study |
| 22 S10_20 | 1766 | 10 | √ | √ | | Push core | This study |
| 22 S10_22 | 1766 | 10 | √ | | | Push core | This study |
| 22 S10_24 | 1766 | 10 | √ | | | Push core | This study |
| 22 S10_26 | 1766 | 10 | √ | | | Push core | This study |
| 22 S10_28 | 1766 | 10 | √ | √ | √ | Push core | This study |
| 22 S10_30 | 1766 | 10 | √ | | | Push core | This study |
| 22 S10_33 | 1766 | 10 | √ | √ | √ | Push core | This study |
| 22 S10_36.5 | 1766 | 10 | √ | | | Push core | This study |
| 22 N10_0.25 | 1766 | 20 | √ | √ | √ | Push core | This study |
| 22 N10_0.75 | 1766 | 20 | √ | √ | | Push core | This study |
| 22 N10_1.25 | 1766 | 20 | √ | √ | | Push core | This study |
| 22 N10_1.75 | 1766 | 20 | √ | √ | | Push core | This study |

| | | | | | | | |
|---|---|---|---|---|---|---|---|
| 22 N10_2.25 | 1766 | 20 | √ | √ | | Push core | This study |
| 22 N10_2.75 | 1766 | 20 | √ | √ | | Push core | This study |
| 22 N10_3.25 | 1766 | 20 | √ | √ | √ | Push core | This study |
| 22 N10_3.75 | 1766 | 20 | √ | √ | | Push core | This study |
| 22 N10_4.25 | 1766 | 20 | √ | | | Push core | This study |
| 22 N10_4.75 | 1766 | 20 | √ | √ | | Push core | This study |
| 22 N10_5.25 | 1766 | 20 | √ | | | Push core | This study |
| 22 N10_5.75 | 1766 | 20 | √ | √ | | Push core | This study |
| 22 N10_6.25 | 1766 | 20 | √ | √ | | Push core | This study |
| 22 N10_6.75 | 1766 | 20 | √ | | | Push core | This study |
| 22 N10_7.25 | 1766 | 20 | √ | √ | | Push core | This study |
| 22 N10_7.75 | 1766 | 20 | √ | √ | | Push core | This study |
| 22 N10_8.25 | 1766 | 20 | √ | | | Push core | This study |
| 22 N10_8.75 | 1766 | 20 | √ | | | Push core | This study |
| 22 N10_9.25 | 1766 | 20 | √ | √ | | Push core | This study |
| 22 N10_9.75 | 1766 | 20 | √ | √ | | Push core | This study |
| 22 N10_10.5 | 1766 | 20 | √ | √ | | Push core | This study |
| 22 N10_11.5 | 1766 | 20 | √ | √ | | Push core | This study |
| 23 N10_12.5 | 1766 | 20 | √ | | | Push core | This study |
| 22 N10_13.5 | 1766 | 20 | √ | | | Push core | This study |
| 22 N10_14.5 | 1766 | 20 | √ | | | Push core | This study |
| 22 N10_16 | 1766 | 20 | √ | √ | | Push core | This study |
| 22 N10_18 | 1766 | 20 | √ | √ | | Push core | This study |
| 22 N10_20 | 1766 | 20 | √ | | | Push core | This study |
| 22 N10_22 | 1766 | 20 | √ | | | Push core | This study |

| | | | | | | | |
|---|---|---|---|---|---|---|---|
| 22 N10_24 | 1766 | 20 | √ | √ | | Push core | This study |
| 22 N10_26 | 1766 | 20 | √ | | | Push core | This study |
| 22 N10_28 | 1766 | 20 | √ | √ | | Push core | This study |
| 22 N10_30 | 1766 | 20 | √ | | | Push core | This study |
| 22 N10_33 | 1766 | 20 | √ | √ | | Push core | This study |
| 22 N10_37 | 1766 | 20 | √ | | | Push core | This study |
| 22 N10_41 | 1766 | 20 | √ | √ | √ | Push core | This study |
| 22 N10_45 | 1766 | 20 | √ | √ | | Push core | This study |
| 22 N10_49 | 1766 | 20 | √ | √ | | Push core | This study |
| 22 N10_53 | 1766 | 20 | √ | √ | | Push core | This study |
| 22 N10_57 | 1766 | 20 | √ | √ | | Push core | This study |
| 22 N10_61 | 1766 | 20 | √ | √ | | Push core | This study |
| 22 N10_65 | 1766 | 20 | √ | √ | | Push core | This study |
| 22 N10_69 | 1766 | 20 | √ | √ | √ | Push core | This study |
| 22 N10_73 | 1766 | 20 | √ | | | Push core | This study |
| 22 N10_77 | 1766 | 20 | √ | √ | | Push core | This study |
| 22 N10_84.5 | 1766 | 20 | √ | √ | | Push core | This study |
| 22 S20_0.25 | 1766 | 20 | √ | √ | √ | Push core | This study |
| 22 S20_0.75 | 1766 | 20 | √ | √ | | Push core | This study |
| 22 S20_1.25 | 1766 | 20 | √ | √ | | Push core | This study |
| 22 S20_1.75 | 1766 | 20 | √ | √ | | Push core | This study |
| 22 S20_2.25 | 1766 | 20 | √ | √ | | Push core | This study |
| 22 S20_2.75 | 1766 | 20 | √ | √ | | Push core | This study |
| 22 S20_3.25 | 1766 | 20 | √ | √ | | Push core | This study |
| 22 S20_3.75 | 1766 | 20 | √ | √ | | Push core | This study |

| | | | | | | | |
|---|---|---|---|---|---|---|---|
| 22 S20_4.25 | 1766 | 20 | √ | √ | | Push core | This study |
| 22 S20_4.75 | 1766 | 20 | √ | | | Push core | This study |
| 22 S20_5.25 | 1766 | 20 | √ | √ | | Push core | This study |
| 22 S20_5.75 | 1766 | 20 | √ | | | Push core | This study |
| 22 S20_6.25 | 1766 | 20 | √ | √ | | Push core | This study |
| 22 S20_6.75 | 1766 | 20 | √ | √ | | Push core | This study |
| 22 S20_7.25 | 1766 | 20 | √ | √ | | Push core | This study |
| 22 S20_7.75 | 1766 | 20 | √ | | | Push core | This study |
| 22 S20_8.25 | 1766 | 20 | √ | √ | | Push core | This study |
| 22 S20_8.75 | 1766 | 20 | √ | | | Push core | This study |
| 22 S20_9.25 | 1766 | 20 | √ | √ | | Push core | This study |
| 22 S20_9.75 | 1766 | 20 | √ | √ | √ | Push core | This study |
| 22 S20_10.5 | 1766 | 20 | √ | | | Push core | This study |
| 22 S20_11.5 | 1766 | 20 | √ | √ | | Push core | This study |
| 22 S20_12.5 | 1766 | 20 | √ | | | Push core | This study |
| 22 S20_13.5 | 1766 | 20 | √ | √ | | Push core | This study |
| 22 S20_14.5 | 1766 | 20 | √ | √ | | Push core | This study |
| 22 S20_16 | 1766 | 20 | √ | √ | √ | Push core | This study |
| 22 S20_18 | 1766 | 20 | √ | | | Push core | This study |
| 22 S20_20 | 1766 | 20 | √ | √ | | Push core | This study |
| 22 S20_22 | 1766 | 20 | √ | | | Push core | This study |
| 22 S20_24 | 1766 | 20 | √ | | | Push core | This study |
| 22 S20_26 | 1766 | 20 | √ | √ | | Push core | This study |
| 22 S20_28 | 1766 | 20 | √ | | | Push core | This study |
| 22 S20_30 | 1766 | 20 | √ | | | Push core | This study |

| | | | | | | | |
|---|---|---|---|---|---|---|---|
| 22 S20_33 | 1766 | 20 | √ | √ | | Push core | This study |
| 22 S20_37 | 1766 | 20 | √ | √ | | Push core | This study |
| 22 S20_40 | 1766 | 20 | v | √ | √ | Push core | This study |
| 22 N30_0.25 | 1766 | 30 | √ | √ | √ | Push core | This study |
| 22 N30_0.75 | 1766 | 30 | √ | | | Push core | This study |
| 22 N30_1.25 | 1766 | 30 | √ | √ | | Push core | This study |
| 22 N30_1.75 | 1766 | 30 | √ | | | Push core | This study |
| 22 N30_2.25 | 1766 | 30 | √ | | | Push core | This study |
| 22 N30_2.75 | 1766 | 30 | √ | √ | √ | Push core | This study |
| 22 N30_3.25 | 1766 | 30 | √ | √ | | Push core | This study |
| 22 N30_3.75 | 1766 | 30 | √ | | | Push core | This study |
| 22 N30_4.25 | 1766 | 30 | √ | | | Push core | This study |
| 22 N30_4.75 | 1766 | 30 | √ | √ | | Push core | This study |
| 22 N30_5.25 | 1766 | 30 | √ | | | Push core | This study |
| 22 N30_5.75 | 1766 | 30 | √ | | | Push core | This study |
| 22 N30_6.25 | 1766 | 30 | √ | √ | | Push core | This study |
| 22 N30_6.75 | 1766 | 30 | √ | | | Push core | This study |
| 22 N30_7.25 | 1766 | 30 | √ | √ | | Push core | This study |
| 22 N30_7.75 | 1766 | 30 | √ | | | Push core | This study |
| 22 N30_8.25 | 1766 | 30 | √ | √ | | Push core | This study |
| 22 N30_8.75 | 1766 | 30 | √ | | | Push core | This study |
| 22 N30_9.25 | 1766 | 30 | √ | √ | | Push core | This study |
| 22 N30_9.75 | 1766 | 30 | √ | | | Push core | This study |
| 22 N30_10.5 | 1766 | 30 | √ | | | Push core | This study |
| 22 N30_11.5 | 1766 | 30 | √ | √ | | Push core | This study |

| | | | | | | | |
|---|---|---|---|---|---|---|---|
| 22 N30_12.5 | 1766 | 30 | √ | | | Push core | This study |
| 22 N30_13.5 | 1766 | 30 | √ | | | Push core | This study |
| 22 N30_14.5 | 1766 | 30 | √ | | | Push core | This study |
| 22 N30_16 | 1766 | 30 | √ | √ | √ | Push core | This study |
| 22 N30_18 | 1766 | 30 | √ | | | Push core | This study |
| 22 N30_20 | 1766 | 30 | √ | | | Push core | This study |
| 22 N30_22 | 1766 | 30 | √ | | | Push core | This study |
| 22 N30_24 | 1766 | 30 | √ | | | Push core | This study |
| 22 N30_26 | 1766 | 30 | √ | √ | | Push core | This study |
| 22 N30_28 | 1766 | 30 | √ | √ | | Push core | This study |
| 22 N30_30 | 1766 | 30 | √ | √ | √ | Push core | This study |
| 22 W10_10 | 1766 | 10 | √ | √ | | Gravity/piston core | This study |
| 22 W10_30 | 1766 | 10 | √ | √ | | Gravity/piston core | This study |
| 22 W10_50 | 1766 | 10 | √ | √ | | Gravity/piston core | This study |
| 22 W10_70 | 1766 | 10 | √ | √ | | Gravity/piston core | This study |
| 22 W10_110 | 1766 | 10 | √ | √ | | Gravity/piston core | This study |
| 22 W10_150 | 1766 | 10 | √ | √ | | Gravity/piston core | This study |
| 23 S10_5 | 1766 | 10 | √ | √ | | Push core | This study |
| 23 S10_15 | 1766 | 10 | √ | √ | | Push core | This study |
| 23 S10_25 | 1766 | 10 | √ | √ | | Push core | This study |
| 23 N10_1 | 1766 | 10 | √ | √ | | Push core | This study |
| 23 N10_3 | 1766 | 10 | √ | √ | | Push core | This study |
| 23 N10_5 | 1766 | 10 | √ | | | Push core | This study |
| 23 N10_7 | 1766 | 10 | √ | √ | | Push core | This study |
| 23 N10_9 | 1766 | 10 | √ | √ | | Push core | This study |

| | | | | | | | |
|---|---|---|---|---|---|---|---|
| 23 N10_12 | 1766 | 10 | √ | √ | | Push core | This study |
| 23 N10_16 | 1766 | 10 | √ | √ | | Push core | This study |
| 23 N10_20 | 1766 | 10 | √ | √ | | Push core | This study |
| 23 N10_24 | 1766 | 10 | √ | √ | | Push core | This study |
| 23 N10_28 | 1766 | 10 | √ | √ | | Push core | This study |
| 23 N10_30 | 1766 | 10 | √ | | | Push core | This study |
| 23 E20_3 | 1766 | 20 | √ | √ | | Push core | This study |
| 23 E20_5 | 1766 | 20 | √ | √ | | Push core | This study |
| 23 E20_7 | 1766 | 20 | √ | √ | | Push core | This study |
| 23 E20_9 | 1766 | 20 | √ | √ | | Push core | This study |
| 23 E20_12 | 1766 | 20 | √ | √ | | Push core | This study |
| 23 E20_14 | 1766 | 20 | √ | √ | | Push core | This study |
| 23 E20_16 | 1766 | 20 | √ | √ | | Push core | This study |
| 23 E20_20 | 1766 | 20 | √ | √ | | Push core | This study |
| 23 E20_24 | 1766 | 20 | √ | √ | | Push core | This study |
| 23 E20_28 | 1766 | 20 | √ | | | Push core | This study |
| 23 E20_32 | 1766 | 20 | √ | √ | | Push core | This study |
| 23 W10 10 | 1766 | 10 | √ | √ | | Gravity/piston core | This study |
| 23 W10 30 | 1766 | 10 | √ | √ | | Gravity/piston core | This study |
| 23 W10 50 | 1766 | 10 | √ | √ | | Gravity/piston core | This study |
| 23 W10 70 | 1766 | 10 | √ | √ | | Gravity/piston core | This study |
| 23 W10 110 | 1766 | 10 | √ | √ | | Gravity/piston core | This study |
| 23 W10 150 | 1766 | 10 | √ | √ | | Gravity/piston core | This study |
| 20 ES120 5 | 1766 | 120 | √ | √ | | Push core | This study |
| 20 ES120 15 | 1766 | 120 | √ | √ | | Push core | This study |

| | | | | | | | |
|---|---|---|---|---|---|---|---|
| 20 ES120 25 | 1766 | 120 | √ | √ | | Push core | This study |
| 20 ES300_5 | 1766 | 300 | √ | √ | | Push core | This study |
| 20 ES300_15 | 1766 | 300 | √ | √ | | Push core | This study |
| 20 ES300_25 | 1766 | 300 | √ | √ | | Push core | This study |
| 20 S400_5 | 1766 | 400 | √ | √ | | Push core | This study |
| 20 S400_15 | 1766 | 400 | √ | √ | | Push core | This study |
| 20 S400_25 | 1766 | 400 | √ | √ | | Push core | This study |
| 21 N50_5 | 1766 | 50 | √ | √ | | Push core | This study |
| 21 N50_15 | 1766 | 50 | √ | √ | | Push core | This study |
| 21 E200_5 | 1766 | 200 | √ | √ | | Push core | This study |
| 21 E200_15 | 1766 | 200 | √ | √ | | Push core | This study |
| 21 E200_25 | 1766 | 200 | √ | √ | | Push core | This study |
| 21 E200_35 | 1766 | 200 | √ | √ | | Push core | This study |
| 21 N100_5 | 1766 | 100 | √ | √ | | Push core | This study |
| 21 N100_15 | 1766 | 100 | √ | √ | | Push core | This study |
| 21 N100_25 | 1766 | 100 | √ | √ | | Push core | This study |
| 21 N100_35 | 1766 | 100 | √ | √ | | Push core | This study |
| 21 N300_5 | 1766 | 300 | √ | √ | | Push core | This study |
| 21 N300_15 | 1766 | 300 | √ | | | Push core | This study |
| 21 N300_25 | 1766 | 300 | √ | √ | | Push core | This study |
| 21 N300_35 | 1766 | 300 | √ | √ | | Push core | This study |
| 21 N500_5 | 1766 | 500 | √ | √ | | Push core | This study |
| 21 N500_15 | 1766 | 500 | √ | √ | | Push core | This study |
| 21 N500_25 | 1766 | 500 | √ | √ | | Push core | This study |
| 21 S100_5 | 1766 | 100 | √ | √ | | Push core | This study |

| | | | | | | | |
|---|---|---|---|---|---|---|---|
| 21 | S100_15 | 1766 | 100 | √ | √ | Push core | This study |
| 21 | S100_25 | 1766 | 100 | √ | | Push core | This study |
| 21 | S100_35 | 1766 | 100 | √ | √ | Push core | This study |
| 21 | S300_5 | 1766 | 300 | √ | √ | Push core | This study |
| 21 | S300_15 | 1766 | 300 | √ | √ | Push core | This study |
| 21 | S300_25 | 1766 | 300 | √ | √ | Push core | This study |
| 21 | S300_35 | 1766 | 300 | √ | √ | Push core | This study |
| 21 | S500_5 | 1766 | 500 | √ | √ | Push core | This study |
| 21 | S500_15 | 1766 | 500 | √ | | Push core | This study |
| 21 | S500_25 | 1766 | 500 | √ | √ | Push core | This study |
| 22 | E50_5 | 1766 | 50 | √ | √ | Push core | This study |
| 22 | E50_15 | 1766 | 50 | √ | √ | Push core | This study |
| 22 | E50_25 | 1766 | 50 | √ | √ | Push core | This study |
| 22 | E50_35 | 1766 | 50 | √ | √ | Push core | This study |
| 22 | E50_45 | 1766 | 50 | √ | √ | Push core | This study |
| 22 | E50_55 | 1766 | 50 | √ | √ | Push core | This study |
| 22 | N50_5 | 1766 | 50 | √ | √ | Push core | This study |
| 22 | N50_15 | 1766 | 50 | √ | √ | Push core | This study |
| 22 | N50_25 | 1766 | 50 | √ | √ | Push core | This study |
| 22 | N50_35 | 1766 | 50 | √ | √ | Push core | This study |
| 22 | N50_45 | 1766 | 50 | √ | √ | Push core | This study |
| 22 | S50_5 | 1766 | 50 | √ | √ | Push core | This study |
| 22 | S50_15 | 1766 | 50 | √ | √ | Push core | This study |
| 22 | S50_25 | 1766 | 50 | √ | √ | Push core | This study |
| 22 | S50_35 | 1766 | 50 | √ | √ | Push core | This study |

| | | | | | | |
|---|---|---|---|---|---|---|
| 22 W100_5 | 1766 | 100 | √ | √ | Push core | This study |
| 22 W100_15 | 1766 | 100 | √ | √ | Push core | This study |
| 22 W100_25 | 1766 | 100 | √ | √ | Push core | This study |
| 22 W100_35 | 1766 | 100 | √ | √ | Push core | This study |
| 22 W100_45 | 1766 | 100 | √ | √ | Push core | This study |
| 22 W50_5 | 1766 | 50 | √ | √ | Push core | This study |
| 22 W50_15 | 1766 | 50 | √ | √ | Push core | This study |
| 22 W50_25 | 1766 | 50 | √ | √ | Push core | This study |
| 22 W50_35 | 1766 | 50 | √ | √ | Push core | This study |
| 22 W50_45 | 1766 | 50 | √ | √ | Push core | This study |
| 23 E30_5 | 1766 | 30 | √ | √ | Push core | This study |
| 23 E30_15 | 1766 | 30 | √ | √ | Push core | This study |
| 23 E30_25 | 1766 | 30 | √ | √ | Push core | This study |
| 23 E30_35 | 1766 | 30 | √ | √ | Push core | This study |
| 23 E30_5 | 1766 | 30 | √ | | Push core | This study |
| 23 E50_15 | 1766 | 50 | √ | √ | Push core | This study |
| 23 E50_25 | 1766 | 50 | √ | √ | Push core | This study |
| 23 E50_35 | 1766 | 50 | √ | √ | Push core | This study |
| 23 N30_5 | 1766 | 30 | √ | √ | Push core | This study |
| 23 N30_15 | 1766 | 30 | √ | √ | Push core | This study |
| 23 N30_25 | 1766 | 30 | √ | √ | Push core | This study |
| 23 N50_5 | 1766 | 50 | √ | √ | Push core | This study |
| 23 N50_15 | 1766 | 50 | √ | √ | Push core | This study |
| 23 N50_25 | 1766 | 50 | √ | √ | Push core | This study |
| 23 N50_35 | 1766 | 50 | √ | √ | Push core | This study |

| | | | | | | | |
|---|---|---|---|---|---|---|---|
| 23 S30_5 | 1766 | 30 | √ | √ | | Push core | This study |
| 23 S30_15 | 1766 | 30 | √ | √ | | Push core | This study |
| 23 S30_25 | 1766 | 30 | √ | √ | | Push core | This study |
| 23 S50_5 | 1766 | 50 | √ | √ | | Push core | This study |
| 23 S50_15 | 1766 | 50 | √ | √ | | Push core | This study |
| 23 S50_25 | 1766 | 50 | √ | √ | | Push core | This study |
| 23 S50_35 | 1766 | 50 | √ | √ | | Push core | This study |
| 23 W30_5 | 1766 | 30 | √ | √ | | Push core | This study |
| 23 W30_15 | 1766 | 30 | √ | √ | | Push core | This study |
| 23 W50_5 | 1766 | 50 | √ | √ | | Push core | This study |
| 23 W50_15 | 1766 | 50 | √ | √ | | Push core | This study |
| 23 W50_25 | 1766 | 50 | √ | √ | | Push core | This study |
| 23 W50_35 | 1766 | 50 | √ | √ | | Push core | This study |
| 21 S18_HK_5 | 1720 | >10,000 | √ | √ | | Push core | This study |
| 21 S18_HK_15 | 1720 | >10,000 | √ | √ | | Push core | This study |
| 21 S18_HK_22.5 | 1720 | >10,000 | √ | √ | | Push core | This study |
| 21 S18_HS_0 | 1720 | >10,000 | √ | √ | | Push core | This study |
| 21 S18_HS_15 | 1720 | >10,000 | √ | √ | | Push core | This study |
| 21 S18_PC22_10 | 1720 | >10,000 | √ | √ | | Gravity/piston core | This study |
| 21 S18_PC22_50 | 1720 | >10,000 | √ | √ | | Gravity/piston core | This study |
| 21 S18_PC22_90 | 1720 | >10,000 | √ | √ | | Gravity/piston core | This study |
| 21 S18_PC22_130 | 1720 | >10,000 | √ | √ | | Gravity/piston core | This study |
| 21 HM_HK_5 | 1380 | >10,000 | √ | √ | | Push core | This study |
| 21 HM_HK_15 | 1380 | >10,000 | √ | √ | | Push core | This study |
| 21 HM_HK_25 | 1380 | >10,000 | √ | √ | | Push core | This study |

| | | | | | | |
|---|---|---|---|---|---|---|
| 21 HM_YB_5 | 1380 | >10,000 | √ | √ | Push core | This study |
| 21 HM_YB_15 | 1380 | >10,000 | √ | √ | Push core | This study |
| 21 HM_YB_25 | 1380 | >10,000 | √ | √ | Push core | This study |
| 21 HM_YB_35 | 1380 | >10,000 | √ | √ | Push core | This study |
| 21 HM_YB_45 | 1380 | >10,000 | √ | √ | Push core | This study |
| 21 HM_YB_55 | 1380 | >10,000 | √ | √ | Push core | This study |
| 21 HM_YB_65 | 1380 | >10,000 | √ | √ | Push core | This study |
| 21 HM_YB_75 | 1380 | >10,000 | √ | √ | Push core | This study |
| 21 HM_YBHK_5 | 1380 | >10,000 | √ | √ | Push core | This study |
| 21 HM_YBHK_15 | 1380 | >10,000 | √ | √ | Push core | This study |
| 21 HM_YBHK_25 | 1380 | >10,000 | √ | √ | Push core | This study |
| 21 HM_YBHK_35 | 1380 | >10,000 | √ | √ | Push core | This study |
| 21 HM_YBHK_45 | 1380 | >10,000 | √ | √ | Push core | This study |
| 21 HM_YBHK_52.5 | 1380 | >10,000 | √ | √ | Push core | This study |
| 21 HM1_1 | 1380 | >10,000 | √ | √ | Push core | This study |
| 21 HM1_3 | 1380 | >10,000 | √ | √ | Push core | This study |
| 21 HM1_5 | 1380 | >10,000 | √ | √ | Push core | This study |
| 21 HM1_7 | 1380 | >10,000 | √ | √ | Push core | This study |
| 21 HM1_9 | 1380 | >10,000 | √ | √ | Push core | This study |
| 21 HM1_11 | 1380 | >10,000 | √ | √ | Push core | This study |
| 21 HM1_13 | 1380 | >10,000 | √ | √ | Push core | This study |

| | | | | | | |
|---|---|---|---|---|---|---|
| 21 HM1_15 | 1380 | >10,000 | √ | √ | Push core | This study |
| 21 HM1_17 | 1380 | >10,000 | √ | √ | Push core | This study |
| 21 HM1_19 | 1380 | >10,000 | √ | √ | Push core | This study |
| 21 HM1_21 | 1380 | >10,000 | √ | √ | Push core | This study |
| 21 HM1_23 | 1380 | >10,000 | √ | √ | Push core | This study |
| 19 HM1_3 | 1380 | >10,000 | | | Push core | |
| 19 HM1_11 | 1380 | >10,000 | | | Push core | |
| 19 HM1_19 | 1380 | >10,000 | | | Push core | [27] |
| 19 HM1_27a | 1380 | >10,000 | | | Push core | |
| 19 HM1_27b | 1380 | >10,000 | | | Push core | |
| 21 HM2_1 | 1380 | >10,000 | √ | √ | Push core | This study |
| 21 HM2_3 | 1380 | >10,000 | √ | √ | Push core | This study |
| 21 HM2_7 | 1380 | >10,000 | √ | √ | Push core | This study |
| 21 HM2_11 | 1380 | >10,000 | √ | √ | Push core | This study |
| 21 HM2_13 | 1380 | >10,000 | √ | √ | Push core | This study |
| 21 HM2_15 | 1380 | >10,000 | √ | √ | Push core | This study |
| 21 HM2_17 | 1380 | >10,000 | √ | √ | Push core | This study |
| 21 HM2_19 | 1380 | >10,000 | √ | √ | Push core | This study |
| 21 HM3_1 | 1380 | >10,000 | √ | √ | Push core | This study |
| 21 HM3_3 | 1380 | >10,000 | √ | √ | Push core | This study |
| 21 HM3_5 | 1380 | >10,000 | √ | √ | Push core | This study |
| 21 HM3_7 | 1380 | >10,000 | √ | √ | Push core | This study |
| 21 HM3_9 | 1380 | >10,000 | √ | √ | Push core | This study |
| 21 HM3_11 | 1380 | >10,000 | √ | √ | Push core | This study |
| 21 HM3_13 | 1380 | >10,000 | √ | √ | Push core | This study |

| | | | | | | |
|---|---|---|---|---|---|---|
| 21 HM3_15 | 1380 | >10,000 | √ | √ | Push core | This study |
| 21 HM3_17 | 1380 | >10,000 | √ | √ | Push core | This study |
| 21 HM3_19 | 1380 | >10,000 | √ | √ | Push core | This study |
| 19 HM3_11 | 1380 | >10,000 | | | Push core | |
| 19 HM3_19 | 1380 | >10,000 | | | Push core | |
| 19 HM3_27 | 1380 | >10,000 | | | Push core | |
| 19 HM3_35 | 1380 | >10,000 | | | Push core | |
| 19 HM3_75 | 1380 | >10,000 | | | Push core | |
| 19 HM5_1 | 1380 | >10,000 | | | Push core | [27] |
| 19 HM5_5 | 1380 | >10,000 | | | Push core | |
| 19 HM5_13 | 1380 | >10,000 | | | Push core | |
| 19 HM5_21 | 1380 | >10,000 | | | Push core | |
| 19 HM5_29 | 1380 | >10,000 | | | Push core | |
| 21 SF1_1 | 1150 | >1000,000 | √ | √ | Push core | This study |
| 21 SF1_3 | 1150 | >1000,000 | √ | √ | Push core | This study |
| 21 SF1_5 | 1150 | >1000,000 | √ | √ | Push core | This study |
| 21 SF1_7 | 1150 | >1000,000 | √ | √ | Push core | This study |
| 21 SF1_9 | 1150 | >1000,000 | √ | √ | Push core | This study |
| 21 SF1_11 | 1150 | >1000,000 | √ | √ | Push core | This study |
| 21 SF1_13 | 1150 | >1000,000 | √ | √ | Push core | This study |
| 21 SF1_15 | 1150 | >1000,000 | √ | √ | Push core | This study |
| 21 SF1_17 | 1150 | >1000,000 | √ | √ | Push core | This study |
| 21 SF1_19 | 1150 | >1000,000 | √ | √ | Push core | This study |
| 19 SFb_1 | 1150 | >1000,000 | | | Push core | [28] |
| 19 SFb_7 | 1150 | >1000,000 | | | Push core | |

| | | | | | | | |
|---|---|---|---|---|---|---|---|
| 19 SFb_9 | 1150 | >1000,000 | | | | Push core | |
| 19 SFb_11 | 1150 | >1000,000 | | | | Push core | |
| 19 SFb_17 | 1150 | >1000,000 | | | | Push core | |
| 21 SF2_1 | 1150 | >1000,000 | √ | √ | | Push core | This study |
| 21 SF2_3 | 1150 | >1000,000 | √ | √ | | Push core | This study |
| 21 SF2_5 | 1150 | >1000,000 | √ | √ | | Push core | This study |
| 21 SF2_7 | 1150 | >1000,000 | √ | √ | | Push core | This study |
| 21 SF2_9 | 1150 | >1000,000 | √ | √ | | Push core | This study |
| 21 SF2_11 | 1150 | >1000,000 | √ | √ | | Push core | This study |
| 21 SF2_13 | 1150 | >1000,000 | √ | √ | | Push core | This study |
| 21 SF2_15 | 1150 | >1000,000 | √ | √ | | Push core | This study |
| 21 SF2_17 | 1150 | >1000,000 | √ | √ | | Push core | This study |
| 21 SF2_19 | 1150 | >1000,000 | √ | √ | | Push core | This study |
| 21 SF3_1 | 1150 | >1000,000 | √ | √ | | Push core | This study |
| 21 SF3_3 | 1150 | >1000,000 | √ | √ | | Push core | This study |
| 21 SF3_5 | 1150 | >1000,000 | √ | √ | | Push core | This study |
| 21 SF3_7 | 1150 | >1000,000 | √ | √ | | Push core | This study |
| 21 SF3_9 | 1150 | >1000,000 | √ | √ | | Push core | This study |
| 21 SF3_11 | 1150 | >1000,000 | √ | √ | | Push core | This study |
| 21 SF3_13 | 1150 | >1000,000 | √ | √ | | Push core | This study |
| 21 SF3_15 | 1150 | >1000,000 | √ | √ | | Push core | This study |
| 21 SF3_17 | 1150 | >1000,000 | √ | √ | | Push core | This study |
| 21 SF3_19 | 1150 | >1000,000 | √ | √ | | Push core | This study |
| 21 SF4_1 | 1150 | >1000,000 | √ | √ | | Push core | This study |
| 21 SF4_3 | 1150 | >1000,000 | √ | √ | | Push core | This study |

| | | | | | | | |
|---|---|---|---|---|---|---|---|
| 21 SF4_5 | 1150 | >1000,000 | √ | √ | | Push core | This study |
| 21 SF4_7 | 1150 | >1000,000 | √ | √ | | Push core | This study |
| 21 SF4_9 | 1150 | >1000,000 | √ | √ | | Push core | This study |
| 21 SF4_11 | 1150 | >1000,000 | √ | √ | | Push core | This study |
| 21 SF4_13 | 1150 | >1000,000 | √ | √ | | Push core | This study |
| 21 SF4_15 | 1150 | >1000,000 | √ | √ | | Push core | This study |
| 21 SF4_17 | 1150 | >1000,000 | √ | √ | | Push core | This study |
| 21 SF4_19 | 1150 | >1000,000 | √ | √ | | Push core | This study |

**Supplementary Table 2.** Integrated reaction rates (0-100 cm) derived from reaction-transport modeling (model-1) and Monod biomass-explicit model (Model-2).

| **Reactions** | **Non-seep** | **Newborn Seep-Stage I** | **Newborn Seep-Stage II** | **Mature Seep** |
|---|---|---|---|---|
| | **mmol m$^{-2}$ d$^{-1}$** | **mmol m$^{-2}$ d$^{-1}$** | **mmol m$^{-2}$ d$^{-1}$** | **mmol m$^{-2}$ d$^{-1}$** |
| $CH_4 + 2O_2 \rightarrow HCO_3^- + H^+ + H_2O$ | 0.013-0.019 | 1.687-1.841 | 2.466-2.675 | 0.262-0.287 |
| $CH_4 + SO_4^{2-} \rightarrow HCO_3^- + HS^- + H_2O$ | 0.006-0.010 | 5.504-6.606 | 35.524-45.487 | 52.624-53.064 |
| $CH_4 + 8/5NO_3^- + 8/5H^+ \rightarrow CO_2 + 4/5N_2 + 14/5H_2O$ | 0.000-0.000 | 0.000-0.001 | 0.011-0.016 | 0.003-0.003 |
| $NH_4^+ + 2O_2 \rightarrow NO_3^- + H_2O + 2H^+$ | 0.135-0.139 | 0.800-0.847 | 1.291-1.370 | 0.914-1.072 |
| $H_2S + 2O_2 \rightarrow SO_4^{2-} + 2H^+$ | 0.000-0.000 | 0.154-0.181 | 0.574-0.661 | 1.089-1.131 |
| $NO_3^- + HS^- + H^+ + H_2O \rightarrow NH_4^+ + SO_4^{2-}$ | 0.041-0.044 | 0.831-0.889 | 1.584-1.714 | 1.748-1.952 |
| $(CH_2O)_x (NH_3)_y (H_3PO_4)_z + xO_{2(aq)} \rightarrow xHCO_3^- + yNH_4^+ + zHPO_4^{2-} + (x-y+2z)H^+$ | 0.134-0.139 | 0.447-0.461 | 0.59-0.568 | 0.043-0.05 |
| $(CH_2O)_x (NH_3)_y (H_3PO_4)_z + 4x/5NO_3^- + 24x/5H^+ \rightarrow 2x/5N_2 + xHCO_3^- + yNH_4^+ + zHPO_4^{2-} + H_2O$ | 0.018-0.018 | 0.029-0.030 | 0.019-0.020 | 0.002-0.003 |
| $(CH_2O)_x (NH_3)_y (H_3PO_4)_z + x/2NO_3^- + (y+2z)H^+ \; x/2H_2O \rightarrow (x/2+y)NH_4^+ + xHCO_3^- + zHPO_4^{2-}$ | 0.043-0.043 | 0.068-0.069 | 0.044-0.047 | 0.005-0.006 |
| $(CH_2O)_x (NH_3)_y (H_3PO_4)_z + x/2SO_4^{2-} \rightarrow x/2HS^- + xHCO_3^- + yNH_4^+ + zHPO_4^{2-} + (x/2-y-2z)H^+$ | 0.044-0.048 | 0.123-0.135 | 0.029-0.040 | 0.027-0.030 |
| $(CH2O)_x (NH3)_y (H3PO4)_z + x/2H2O \rightarrow x/2CH_4 + x/2HCO_3^- + yNH_4^+ + zHPO_4^{2-} +(x/2-y+2z) H^+$ | 0.002-0.003 | 0.007-0.008 | 0.008-0.014 | 0.161-0.165 |
| **Total Methane Oxidation-Model-1** | 0.019-0.029 | 7.345-8.447 | 38.198-47.953 (16.4-71.7)* | 52.886-53.351 |
| **Total Methane Oxidation-Model-2** | - | 7.0-44.3 | 31.5-62.2 | 51.450 |

* The value in the bracket was based on $^{14}C$ labelling measurement from two representative cores.

**Supplementary Table 3.** Depth-integrated rates of dissimilatory nitrate reduction to ammonium (DNRA), denitrification, and anammox measured using $^{15}N$-labelling technique in a representative core of the Newborn Seep site.

| Depth interval | Group | DNRA (mmol $m^{-2}$ $d^{-1}$) | | Denitrification (mmol $m^{-2}$ $d^{-1}$) | | Anammox (mmol $m^{-2}$ $d^{-1}$) | | Nitrate reduction (mmol $m^{-2}$ $d^{-1}$) | |
|---|---|---|---|---|---|---|---|---|---|
| | | **Mean** | **SD** | **Mean** | **SD** | **Mean** | **SD** | **Mean** | **SD** |
| **0-16 cm Integrated** | **Total** | 24.86 | 1.06 | 16.17 | 1.06 | 0.30 | 0.07 | 41.33 | 1.50 |
| **0-16 cm Integrated** | **$S^{2-}$ coupled*** | NA† | NA | 2.74 | 1.78 | NA | NA | 2.74 | 1.78 |
| **0-16 cm Integrated** | **$CH_4$ coupled*** | 1.66 | 1.57 | 2.77 | 2.51 | 0.12 | 0.12 | 4.55 | 2.97 |

* The $S^{2-}$ or $CH_4$ coupled rates were quantified through comparing the rates with and without the addition of $S^{2-}$ or $CH_4$, respectively.

† NA = not applicable, the measured rates in the treatment of $S^{2-}$ addition were consistently lower than those of the control group.

**Supplementary Table 4.** Information of the sample types and treatments.

| Type | Targeted parameter | Preservation temperature (◦C ) | Sampling and treatment | Sampled volume (ml)/weight (g) |
|---|---|---|---|---|
| Porewater | Anions ($NO^{3-}$, $NO^{2-}$, $SO_4^{2-}$) | 4 | Taken by Rhizon sampler with 0.15 µm pore size, stored in 5 ml glass vials and fixed with 50 µL of 30 % HCl. | 3-5 ml |
| Porewater | Cations ($NH_4^+$, $Fe^{2+}$, $Mn^{2+}$) | 4 | Taken by Rhizon sampler with 0.15 µm pore size, stored in 5 ml glass vials and fixed with 5 µL of NaOH (2 M). | 3-5 ml |
| Porewater | $HS^-$ | -20 | Taken by Rhizon sampler with 0.15 µm pore size, stored in 2 ml Eppendorf centrifuge tubes and fixed with 1 mL of 5 % zinc acetate. | 1 ml |
| Porewater | DIC, $\delta^{13}$C-DIC | 4 | Injected into the $N_2$-flushed, $CO_2$-free glass tubes (20 ml) sealed with rubber stoppers. | 2-3 ml |
| Sediment | DNA, RNA | -80 | Taken by cut-off 5 ml sterile syringe and stored in Eppendorf cryovials for DNA/RNA samples | >10 g |
| Sediment | TOC, TN, $\delta^{13}$C-TOC, $\delta^{15}$N-TN | -20/-80 | Taken by cut-off 5 ml sterile syringe and stored in 15 ml sterile falcon tubes. | >10 g |
| Sediment | $CH_4$, $\delta^{13}$C-CH4 | 4 | Taken by cut-off 5 ml sterile syringe, transferred to 20 mL crimp vials containing 5 mL saturated NaCl solution, crimped, thoroughly homogenized by shaking. | 2 ml |
| Sediment | $H_2$ | 4 | Taken by cut-off 5 ml sterile syringe, transferred to 20 mL crimp vials containing 5 mL saturated NaCl solution, crimped, thoroughly homogenized by shaking. | 2 ml |

**Supplementary Table 5.** Key biogeochemical reactions implemented in the reaction-transport model.

| Organic matter remineralization | Rate Laws and Constants | constants |
|---|---|---|
| $(CH_2O)_x (NH_3)_y (H_3PO_4)_z + xO_{2(aq)} \rightarrow xHCO_3^- + yNH_4^+ + zHPO_4^{2-} + (x-y+2z)H^+$ * | $R_{aer} = R_c \frac{[O_2]}{(K_{m,o2}+[O_2])}$ | $K_{m,o2}$ = 20 uM |
| $(CH_2O)_x (NH_3)_y (H_3PO_4)_z + \frac{4x}{5}NO_3^- + \frac{24x}{5} H^+ \rightarrow \frac{2x}{5}N_2 + xHCO_3^- + yNH_4^+ + zHPO_4^{2-} + \frac{2x}{5}H_2O$ | $R_{DNF} = (R_c - R_{aer}) \frac{[NO_3^-]}{(K_{mDNF,no3}+[NO_3^-])} * f_{DNF}$ † | $K_{mDNF,no3}$ = 2 uM |
| $(CH_2O)_x (NH_3)_y (H_3PO_4)_z + \frac{x}{2}NO_3^- + (y+2z)H^+ + \frac{x}{2} H_2O \rightarrow (\frac{x}{2} + y)NH_4^+ + xHCO_3^- + zHPO_4^{2-}$ | $R_{DNRA} = (R_c - R_{aer}) \frac{[NO_3^-]}{(K_{mDNRA,no3}+[NO_3^-])} *(1-f_{DNF})$ | $K_{mDNRA,no3}$ = 2 uM (set to $K_{mDNF,no3}$) |
| $(CH_2O)_x (NH_3)_y (H_3PO_4)_z + \frac{x}{2}SO_4^{2-} \rightarrow \frac{x}{2}HS^- + xHCO_3^- + yNH_4^+ + zHPO_4^{2-} + (\frac{x}{2}-y-2z)H^+$ | $R_{SR} = (R_c-R_{aer}-R_{DNF}) \frac{[SO_4^{2-}]}{(K_{m,so4}+[SO_4^{2-}])}$ | $K_{m,so4}$ = 1.6 mM |
| $(CH_2O)_x (NH_3)_y (H_3PO_4)_z + \frac{x}{2} H_2O \rightarrow \frac{x}{2}CH_4 + \frac{x}{2}HCO_3^- + yNH_4^+ + zHPO_4^{2-} + (\frac{x}{2} -y +2z) H^+$ | $R_{MOG} = R_c-R_{aer}-R_{DNF}-R_{SR}$ | - |
| $NO_3^- + HS^- + H^+ + H_2O \rightarrow NH_4^+ + SO_4^{2-}$ | $R_{no3ts} = k_{no3ts} [TS][NO_3^-]$ | $k_{no3ts} = 5\times10^6$ /M/yr |
| $H_2S + 2O_2 \rightarrow SO_4^{2-} + 2H^+$ | $R_{sox} = k_{sox} [TS][O_2]$ | $k_{sox} = 1.6\times10^5$/M/yr |
| $NH_4^+ + 2O_2 \rightarrow NO_3^- + H_2O + 2H^+$ | $R_{nitri} = k_{nitri} [NH_4^+][O_2]$ | $k_{nitri} = 5\times10^6$ /M/yr |
| $CH_4 + 2O_2 \rightarrow HCO_3^- + H^+ + H_2O$ | $R_{ch4o2} = k_{ch4o2} [CH_4][O_2]$ | $k_{ch4o2} = 10^{10}$ /M/yr |
| $CH_4 + SO_4^{2-} \rightarrow HCO_3- + HS- + H_2O$ | $R_{ch4so4} = k_{ch4so4} [CH_4][SO_4^{2-}]$ | $k_{aom} = 10^7$/M/yr |
| $CH_4 + 8/5NO^{3-} + 8/5H^+ \rightarrow CO_2 + 4/5N_2 + 14/5H_2O$ | $R_{ch4no3} = k_{ch4no3} [CH_4][NO^{3-}]$ | $k_{ch4no3} = 5\times10^6$/M/yr |

* x, y, z are 1, 16/106 and 1/106, respectively, representing the organic matter stoichiometry.

† $f_{DNF}$ was set to 0.3.

## Supplementary references:


1. Geng, M., et al., *Focused Fluid Flow, Shallow Gas Hydrate, and Cold Seep in the Qiongdongnan Basin, Northwestern South China Sea.* Geofluids, 2021. **2021**(1): p. 5594980.
2. Deng, L., et al., *Macrofaunal control of microbial community structure in continental margin sediments.* Proceedings of the National Academy of Sciences, 2020. **117**(27): p. 15911-15922.
3. Wang, Y. and P. Van Cappellen, *A multicomponent reactive transport model of early diagenesis: Application to redox cycling in coastal marine sediments.* Geochimica et Cosmochimica Acta, 1996. **60**(16): p. 2993-3014.
4. Dale, A.W., et al., *Pathways and regulation of carbon, sulfur and energy transfer in marine sediments overlying methane gas hydrates on the Opouawe Bank (New Zealand).* Geochimica et Cosmochimica Acta, 2010. **74**(20): p. 5763-5784.
5. Nauhaus, K., et al., *In vitro cell growth of marine archaeal-bacterial consortia during anaerobic oxidation of methane with sulfate.* Environmental microbiology, 2007. **9**(1): p. 187-196.
6. An, S.M. and W.S. Gardner, *Dissimilatory nitrate reduction to ammonium (DNRA) as a nitrogen link, versus denitrification as a sink in a shallow estuary (Laguna Madre/Baffin Bay, Texas).* Marine Ecology Progress Series, 2002. **237**: p. 41-50.
7. Thamdrup, B. and T. Dalsgaard, *Production of N-2 through anaerobic ammonium oxidation coupled to nitrate reduction in marine sediments.* Applied and Environmental Microbiology, 2002. **68**(3): p. 1312-1318.
8. Zhang, S., Y. Fang, and D. Xi, *Adaptation of micro-diffusion method for the analysis of 15N natural abundance of ammonium in samples with small volume.* Rapid Communications in Mass Spectrometry, 2015. **29**(14): p. 1297-1306.
9. Natarajan, V., et al., *A Modified SDS-Based DNA Extraction Method for High Quality Environmental DNA from Seafloor Environments.* Frontiers in microbiology, 2016. **07**.
10. Niu, M., et al., *Methane supply drives prokaryotic community assembly and networks at cold seeps of the South China Sea.* Mol Ecol, 2022.
11. Caporaso, J.G., et al., *Global patterns of 16S rRNA diversity at a depth of millions of sequences per sample.* Proceedings of the National Academy of Sciences, 2011. **108**(supplement_1): p. 4516-4522.
12. Bolyen, E., et al., *Reproducible, interactive, scalable and extensible microbiome data science using QIIME 2.* Nature biotechnology, 2019. **37**(8): p. 852-857.
13. Martin, M., *Cutadapt removes adapter sequences from high-throughput sequencing reads.* EMBnet. journal, 2011. **17**(1): p. 10-12.
14. Callahan, B.J., et al., *DADA2: High-resolution sample inference from Illumina amplicon data.* Nature methods, 2016. **13**(7): p. 581-583.
15. Bokulich, N.A., et al., *Optimizing taxonomic classification of marker-gene amplicon sequences with QIIME 2's q2-feature-classifier plugin.* Microbiome, 2018. **6**(1):

p. 1-17.
16. Quast, C., et al., *The SILVA ribosomal RNA gene database project: improved data processing and web-based tools.* Nucleic acids research, 2012. **41**(D1): p. D590-D596.
17. Bolger, A.M., M. Lohse, and B. Usadel, *Trimmomatic: a flexible trimmer for Illumina sequence data.* Bioinformatics, 2014. **30**(15): p. 2114-2120.
18. Kopylova, E., L. Noé, and H. Touzet, *SortMeRNA: fast and accurate filtering of ribosomal RNAs in metatranscriptomic data.* Bioinformatics, 2012. **28**(24): p. 3211-3217.
19. Kim, D., et al., *Graph-based genome alignment and genotyping with HISAT2 and HISAT-genotype.* Nature biotechnology, 2019. **37**(8): p. 907-915.
20. Liao, Y., G.K. Smyth, and W. Shi, *featureCounts: an efficient general purpose program for assigning sequence reads to genomic features.* Bioinformatics, 2014. **30**(7): p. 923-930.
21. Jorgensen, S.L., et al., *Correlating microbial community profiles with geochemical data in highly stratified sediments from the Arctic Mid-Ocean Ridge.* Proc Natl Acad Sci U S A, 2012. **109**(42): p. E2846-55.
22. Ovreås, L., et al., *Distribution of bacterioplankton in meromictic Lake Saelenvannet, as determined by denaturing gradient gel electrophoresis of PCR-amplified gene fragments coding for 16S rRNA.* Applied and environmental microbiology, 1997. **63**(9): p. 3367-3373.
23. McMurdie, P.J. and S. Holmes, *phyloseq: an R package for reproducible interactive analysis and graphics of microbiome census data.* PLoS One, 2013. **8**(4): p. e61217.
24. Oksanen, J., et al., *Package 'vegan'.* Community ecology package, version, 2013. **2**(9): p. 1-295.
25. Guo, X., et al., *Climate warming leads to divergent succession of grassland microbial communities.* Nature Climate Change, 2018. **8**(9): p. 813-818.
26. Stock, B., et al., *Package 'MixSIAR'.* Bayesian Mixing Model s in R, Version, 2018. **3**(10).
27. Lu, R., et al., *Asgard archaea in the haima cold seep: Spatial distribution and genomic insights.* Deep Sea Research Part I: Oceanographic Research Papers, 2021. **170**: p. 103489.
28. Li, W.L., et al., *Microbial ecology of sulfur cycling near the sulfate–methane transition of deep-sea cold seep sediments.* Environmental Microbiology, 2021. **23**(11): p. 6844-6858.
